# Systematic scoping review of the concept of ‘genetic identity’ and its relevance for germline modification

**DOI:** 10.1371/journal.pone.0228263

**Published:** 2020-01-24

**Authors:** Floor M. Goekoop, Carla G. van El, Guy A. M. Widdershoven, Nadza Dzinalija, Martina C. Cornel, Natalie Evans

**Affiliations:** 1 Amsterdam UMC, Vrije Universiteit Amsterdam, Department of Medical Humanities, De Boelelaan, Amsterdam, Netherlands; 2 Amsterdam UMC, Vrije Universiteit Amsterdam, Department of Clinical Genetics, Section Community Genetics, Amsterdam Public Health research institute, Amsterdam, the Netherlands; Universitat Luzern, SWITZERLAND

## Abstract

EU legislation prohibits clinical trials that modify germ line ‘genetic identity’. ‘Genetic identity’ however, is left undefined. This study aims to identify the use of the term ‘genetic identity’ in academic literature, and investigate its relevance for debates on genetic modification. A total of 616 articles that contained the term were identified. Content analysis revealed that the term was used in various and contradicting ways and a clear understanding of the term is lacking. This review demonstrates that the EU legislation is open to interpretation, because of the diversity of meaning with which ‘genetic identity’ is currently used. Because of the diversity of meaning with which ‘genetic identity’ is used and understood, further reflection is needed. This requires further medical, legal, ethical and social debate and a coordinated response at both a European and a global level.

## Introduction

The possibility of modifying a person’s genes is no longer confined to the realms of science fiction. Genetic modification has been a field of interest for decades, however recent developments have caused a revolution in the field of biology [1]. Various methods are in use of which CRISPR, a technique that emerged in 2012, has gained much prominence. CRISPR stands for Clustered Regularly Interspaced Short Palindromic Repeats (CRISPR), a type of DNA sequence that has been found in certain bacteria. Together with an endonuclease (CRISPR-associated protein, e.g. Cas9), it can target specific DNA sites, cause breaks and subsequently allow for repair or removal. Simply said, it can ‘cut and paste’ any DNA sequence of interest [2]. CRISPR is easy, rapid, relatively inexpensive, and provides extraordinary insights into animal and human developmental processes and gene functioning.

One of the potential applications is in human reproduction; CRISPR could potentially correct germline mutations and therefore prevent certain hereditary diseases [3]. Although exciting, these new possibilities give rise to questions regarding the extent to which we can intervene in a person’s genetic make-up. Some people argue that it is a ‘slippery slope’ from curing diseases to far more controversial practices, such as allowing parents to select specific traits in their future child, so-called ‘designer babies’ [4]. Technical challenges still stand in the way of safe human gene therapies, for which further research, including clinical trials in humans, would be needed [5].

An authoritative international and legally binding (for State Parties) instrument for the protection of human rights in the field of biomedicine is the Oviedo Convention. It states that the identity of all human beings should be protected and it speaks of the need for protection of the human being, both as an individual and as a member of the human species [6]. The Convention further states that any intervention aiming to introduce modifications in the genome of descendants cannot be undertaken [6]. However, only 29 states have signed and ratified the Convention, which means it is not legally binding for a large number of countries including European countries like Germany, the United Kingdom, and the Netherlands [7]. Currently in the EU, the Clinical Trials Directive prohibits clinical trials that ‘result in modifications to the subject's germ line genetic identity’ [8]. Member states are obliged to implement this provision in national law. The Clinical Trials Regulation (a binding legislative act, immediately applicable and enforceable in the whole EU) is set to replace the Directive in 2020 [9–10]. The Regulation maintains the provision provided by the Directive [11], and, although both Directive and Regulation contain a provision where definitions are given, ‘genetic identity’ is not defined.

Given the developments in both scientific practice and legislation, it is essential to understand the meaning of the term ‘genetic identity’ and the contexts in which it is currently used. Such an understanding is vital in order to clarify the European legislation and its normative consequences. This study aims to explore the use of the term ‘genetic identity’ within different academic discourses, and relate how it is used to potential interpretations of European legislation, particularly in relation to germline genetic modification. This review systematically identifies all uses of the term ‘genetic identity’ within the academic literature. Content analysis is used to code and categorize its use, and the emerging discourses are related to disciplinary perspective, year of publication, and geographical setting.

## Methods

The study is a systematic scoping review. A scoping review is appropriate to map similarities and differences in the key concepts that form the base of a specific research area, in contrast to a conventional systematic review that addresses a more specific research question [12]. The methodology, however, is similar to that of a systematic review.

### Search strategy

The search strategy followed the PRISMA extension for scoping reviews and the guidelines developed by Peters et al [12–13].

Searches were designed to retrieve articles in which the terms ‘genetic identity’ or ‘genetic identities’ were used in relation to humans. A detailed description of the search terms is provided in S1 Appendix. Searches were performed in databases covering a range of disciplines: PubMed, Web of Science, Scopus, Embase, CINAHL, ATLA Religion Database, PsycINFO, Social Science Research Network, International Bibliography of the Social Sciences (IBSS), The Philosopher’s Index, JSTOR and Hein online.

After duplicate removal, records were screened independently on title and abstract by two reviewers *([initials anonymised for review])*. In case of disagreements, consensus was reached by discussion. Records were excluded if they were written in a language other than English and if they concerned non-human ‘genetic identity’. Afterwards, full-text articles were assessed for eligibility. During the full-text screening, additional in- and exclusion criteria were formulated (Table 1). The remaining articles were included in the synthesis. A subsample (5%, n = 62) was assessed by the second reviewer *([initials anonymised for review])* and showed that no articles that met the inclusion criteria had been excluded.

**Table 1:** Inclusion and exclusion criteria.

Inclusion criteria	Exclusion criteria
English	Non-English
Human genetic identity	Non-human genetic identity
Articles	Books
Full text access	No full-text access
Defined or undefined use of the term	Term only in footnotes or bibliography
	Term only quoted from legislation
	Term not used / found in article
	Duplicates

### Analysis

Full texts of included articles were examined for the term ‘genetic identity’. Content analysis was used to categorize the definitions and context regarding the term ‘genetic identity’. An emergent (inductive) coding approach was used and coding units were defined: sampling units were articles, context units were paragraphs and the recording units, the areas of text coded, provided insight into the defined or undefined use of the term ‘genetic identity’ [14]. Two reviewers *([initials anonymised for review])* collaboratively developed a coding scheme (Table 2). The two reviewers independently coded the context units and subsequently discussed any differences in coding until agreement was reached on all records. The coding scheme, emergent categories and sub categories were also discussed in monthly meetings of an expert multidisciplinary work group comprised of clinical geneticists, sociologists, midwives, theologians, and ethicists from the departments of Medical Humanities and Clinical Genetics of *([institution anonymised for review])* and the Faculty of Religion and Theology of the *([institution anonymised for review])*.

**Table 2:** Coding scheme.

Code	Freq.	Description
A. ANCESTRY AND HERITAGE (325 times coded in 302 articles)		
1. Parentage	125	Genetic parentage and the right or need to know one’s own parentage
2. Genetic relatedness or similarity		
i. Genetically identical	65	Being genetically identical, as opposed to genetic diversity
ii. The sharing of biological characteristics	62	Genetic connectedness and the inheritability and transmittance of certain characteristics
3. Population genetics		
i. Ethnicity or race	48	(The study of) certain ethnic groups, the tracing of people
ii. Probabilities of genetic identity	25	Probability of two genes taken at random being of the same allelic type
B. PERSONAL IDENTITY (242 times coded in 174 articles)		
1 Personal and social identity	94	Relation between personal/social identity and genetic identity
2. Disease identity	42	Concerning genetic disorders or predispositions and the felt responsibility for these conditions
3. Religious and spiritual identity	14	Relation between religious / spiritual identity and genetic identity
4. Gender identity		Genetic identity as having a male or female identity
5. Critiques of genetic determinism or essentialism	31	Including genetic essentialism, genism, ‘true’ identity, determinism
6. External or environmental influences	36	External or environmental influences on identity, including epigenetics
7. Human identity	17	Concerning humanness / species membership as human
C. (THE BEGINNING OF) INDIVIDUALITY (219 times coded in 194 articles)		
1. Uniqueness and autonomy	56	Including the right to uniqueness and the right to a random genetic identity (as opposed to something predetermined), rights and duties and denial of rights
2. The beginning of life	61	Regarding the first determination of (unique) genetic identity of the “child-to-be” (and need for protection), start of personhood and the status of embryo
3. Modification, alteration and selection	102	Including cloning, gene editing, the non-identity problem (protection of future children will alter future children and therefore cannot protect them), wrongful life, parental liability, and the creation of genetic identity as a means to an end (eg. designer babies or siblings of children with leukemia)
D. PRIVACY AND PROPERTY (162 times coded in 147 articles)		
1. Identification and the protection of genetic information	122	Identification in forensics or in familial genetic testing, protection and misuse of sensible information (including discrimination)
2. Durability of genetic identity	6	Concerning DNA’s capacity to survive over long periods of time
3. Ownership of genetic identity and the commercialization of genetic information	34	Concerning gene ownership (often regarding genetic research/patents), ownership of (future) children, gene theft or commercial exploitation
E. REGIONS OF DNA (60 times coded in 49 articles)		
1. Order of one’s DNA	11	Arrangement of genes, base sequence, not further specified
2. Nuclear DNA	10	DNA contained within the nucleus
3. MtDNA	14	DNA contained within the mitochondria, inherited exclusively from mother
4. Y-chromosome DNA	4	DNA contained in the Y chromosome, past only from father to son
5. Germline DNA	7	The DNA in germ cells (egg and sperm cells)
6. (Complete) Genome	14	All genetic material of an organism
F. CELLS AND GENES (coded in 55 articles)		
1. Specific (parts of) human cells including specific (parts of) genes	55	The genetic identity of specific cells, cell lines, parts of cells (e.g. enzymes, membranes), the description of certain loci, deletions, insertions or other genetic defects

Each article’s authors, year, location, discipline and the recording units were summarized in a data extraction table (S2 Appendix, **available at: https://doi.org/10.5281/zenodo.2611190**). Location was documented as the place of work of the first author. If this information was not available, the location of the second author was used. Discipline was documented as highest educational degree of the first author. If this information was not available the current department of work was used. When authors had degrees from different disciplines or worked at multidisciplinary departments, the capacity in which it was written was assessed (article subject, journal discipline). Explicit definitions of ‘genetic identity’ were recorded.

Sub analyses were conducted subsequently to explore a possible connection between the different meanings of ‘genetic identity’ and the year of publication, location or discipline of the included articles. These variables were grouped in categories for analyses purposes (Table 3); location categories were based on the United Nations Geoscheme [15], and the discipline categories were based on the European Research Council (ERC) research domains [16]. Both categories were adapted to the found frequencies and the purpose of this review. The year of publication was sorted into decades (groups of 10 years).

**Table 3:** Discipline, location and time categories.

Categories	Including
Disciplines	
1. Law	Law, criminology, justice & security, legal studies
2. Social and behavioral sciences	Psychology, sociology, social work, anthropology, political science, geography, economics, politics, human ecology, journalism, social development
3. Humanities	Religious studies, philosophy, historical sciences, literature, ethics, bioethics, medical humanities, rhetoric
4. Life sciences	Biology, medicine, immunology, microbiology, bimolecular engineering, psychiatry, legal medicine, genetics, ecology health sciences, forensic genetics, biophysics, computational biology, orthodontics, medical education, cyber genetics, biochemistry, pharmacology
5. Physical sciences and mathematics	Mathematics, physics, technology, statistics
Location	
1. Northern America	United States of America, Canada, Hawaii
2. North and Western Europe	Ireland, the United Kingdom, Belgium, the Netherlands, Germany, France, Denmark, Sweden, Finland, Switzerland
3. South and Eastern Europe	Portugal, Spain, Italy, Vatican, Greece, Slovenia, Russia, Poland
4. Oceania	Australia, New Zealand
5. Asia	China, Korea, Japan, India, Taiwan, Israel, Iran
6. Latin America & Caribbean	Argentina, Chile, Brazil, Colombia, Cuba, Mexico
7. Africa	South Africa, Morocco, Tunisia, Kenya
Time	
1. 1967–1976	
2. 1977–1986	
3. 1987–1996	
4. 1997–2006	
5. 2007–2018	

## Results

### Search and screening

**Fig 1:**
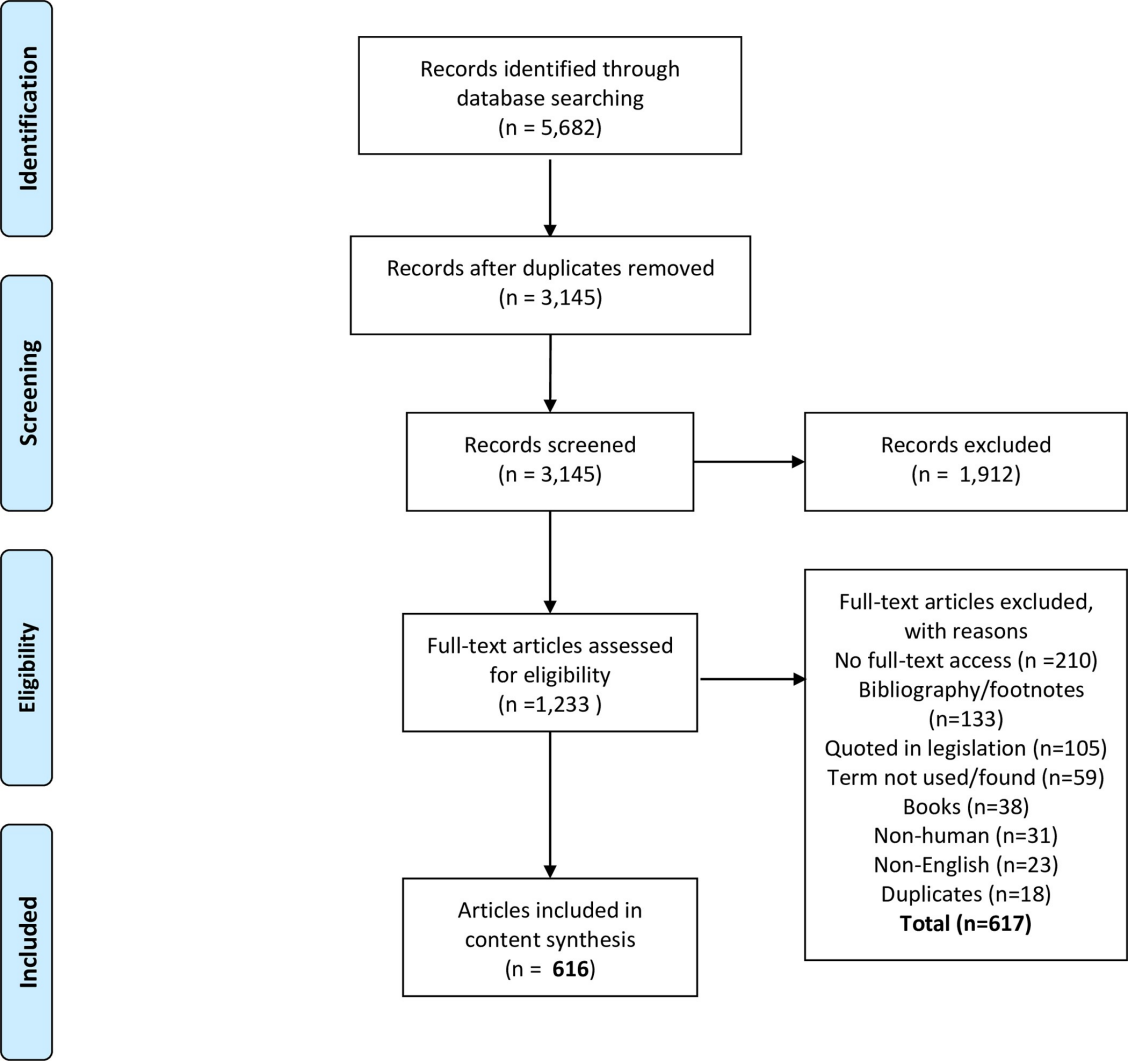
PRISMA flow diagram of article selection for inclusion.

A total of 5,682 records were retrieved. After removing duplicates, 3,145 records were left. Following title-abstract screening 1,233 English records that concerned humans were included. Full-text articles were assessed and a total of 616 articles that contained the term ‘genetic identity’ (or ‘genetic identities’) were finally included based on eligibility (Fig 1).

### Use and meaning of ‘genetic identity’

**Fig 2:**
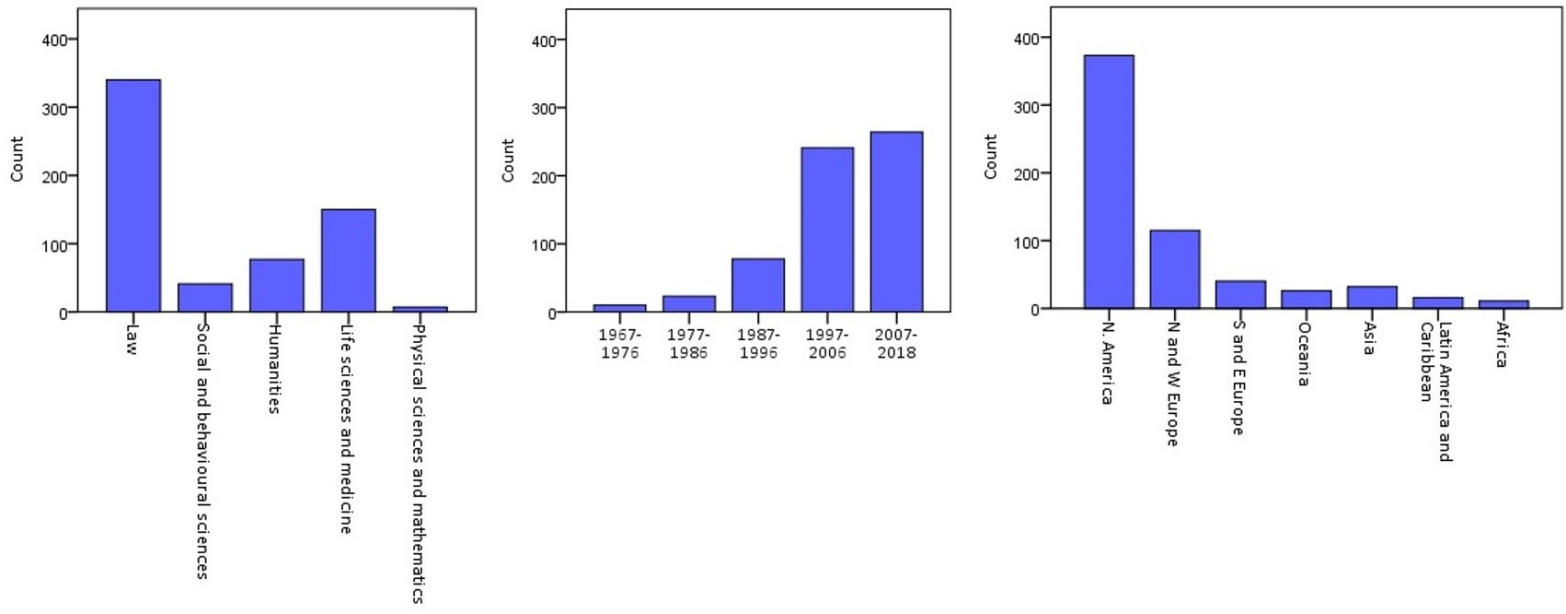
Number of articles using the term ‘genetic identity’ by discipline, location, and year.

The disciplinary perspective, location and year of publication of these articles is depicted in Fig 2. Most authors were located in North America and wrote from a legal perspective. The earliest article in the reviewed literature dated from 1967 [17], and frequencies of articles containing the term increased with time. In the period from 1997 to 2006, the period in which the Human Genome Project was completed [18], the term was encountered more than twice as often as in the decade before.

### Definitions

Few definitions for ‘genetic identity’ were found in literature. Only twenty-five authors defined the term or explained how it was to be understood in the context of their article. These definitions can be found in the ‘defined’ column of the data extraction table (available at: https://doi.org/10.5281/zenodo.2611190) and are discussed in the relevant meaning categories below.

#### Context and meaning

Content analysis revealed six categories that described the context in which the term ‘genetic identity’ was used and its, often implicit, meaning: A) ancestry or heritage, B) personal identity, C) (the beginning of) individuality, D) privacy or property, E) regions of DNA, and F) cells and genes. These categories and the codes from which they are comprised are depicted in Table 2

**Fig 3:**
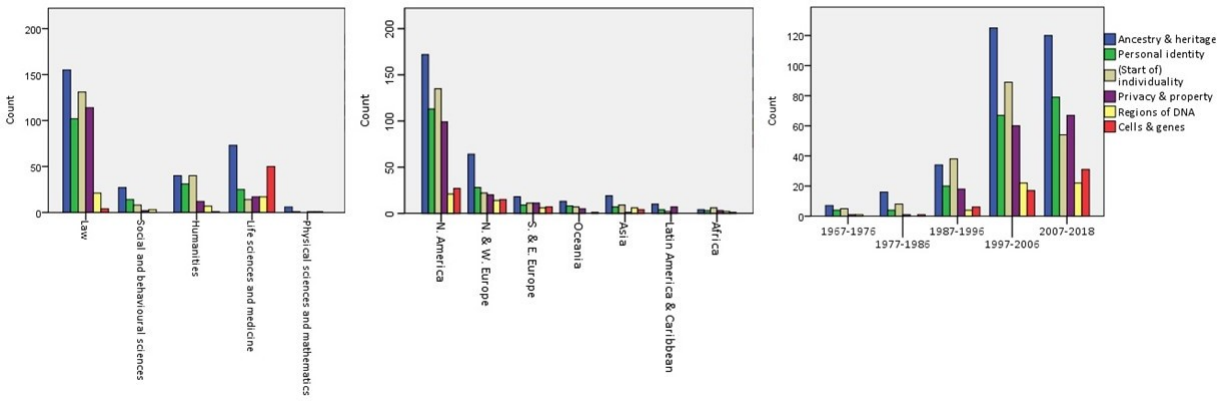
Number of articles for each ‘genetic identity’ meaning category by discipline, location, and year.

The relationship between these meaning categories and articles’ disciplinary perspective, location, and year of publication is depicted in Fig 3. Ancestry and heritage (category A) was most frequently coded, regardless of disciplinary perspective or location. As might be expected, the cells and genes category (category F) was most often used in articles from life sciences and medicine disciplines. Most categories did increase over time, however both ancestry and heritage (category A), and (the beginning of) individuality (category C) peaked in the period from 1997–2006, and declined in the past decade. The cloning of Dolly the sheep [19] and completion of the Human Genome Project [18], respectively, are two influential events that might explain the peak in interest in the (beginning of) individuality category, which includes discussion of cloning, and the peak in the ancestry and heritage category.

## Categories

In this section, the meaning categories that were revealed by content analysis will be discussed, including definitions and relevant quotes found in the reviewed literature.

### Ancestry and heritage (Category A)

‘Genetic identity’ was most frequently used in the context of ancestry and heritage. This category encompasses the subcategories of: parentage; genetic relatedness or similarity; and use in population genetics.

#### Parentage (A1)

‘Genetic identity’ was used in relation to the need to know one’s parentage and the establishment of legal parentage, most often in the legal literature. The importance of knowing one’s parentage was discussed in relation to adoption or anonymous sperm donation. In these cases the courts tend to focus on ‘genetic identity’ (in the sense of biological parentage) when establishing legal parentage, although situations concerning surrogacy and In Vitro Fertilization (IVF) prove even more complicated due to different actors taking on various roles of a parent. Indeed, ‘genetic identity’ was used exclusively to refer to genetic parenthood in the sub-category of parentage.

These cases demonstrate that genetic identity is a key element of the court’s understanding of identity. This is clear from Mikulic and from subsequent cases that deal with the establishment of paternity. The court’s focus was not on the existence of a social relationship between the plaintiffs and the biological father, but simply on the biological truth of the plaintiffs’ paternity and the importance of that to their personal development [20].

Who is the mother? Is it the woman who carried the child for nine months and gave birth, or is it the woman who provided the egg and therefore gave the child its genetic identity? [21]

The painful question of genetic identity and its complexity is evidenced by the nomenclature advanced by Snowden and Mitchell. They contend that there are actually seven distinct roles for the female: genetic mother, carrying mother, nurturing mother, genetic-carrying mother, genetic-nurturing mother, carrying-nurturing mother, and complete mother [22].

#### Genetic relatedness and similarity (A2i and A2ii)

In this subcategory, ‘genetic identity’ was used to mean ‘genetically identical’ in the case of identical twins (or clones), or the term was used in reference to the sharing of certain biological characteristics between people, families or species. These shared characteristics were, for example, associated with altruistic behaviour, while other articles emphasized the inheritability of these characters. Definitions of ‘genetic identity’ in this subcategory include something that ‘as opposed to genetic uniqueness, has to do with the extent to which our genes are shared with others’ [23]; ‘the binding together of people on the basis of a particular genetic characteristic they share’ [24]; something to be constructed ‘in terms of inheritable species-typical biological characteristics, and inheritable anomalous biological characteristics’ [25]; and involving ‘some ‘defining characteristic’ reappearing in each member of a sequence or family of occasions’ [26]. ‘Genetic identity’ is used and defined to mean ‘identical’, ‘similar’ or ‘related’ by descent in this subcategory.

Deliberately bringing about genetic identity can be ethically reprehensible if and only if, by being made genetically identical, the persons who are thus born are deprived of something that is their right: namely, the right to genetic uniqueness. As has already been indicated, the claim that there is such a right is of dubious validity. [27].

Religion enters into the center of the debate for Burhoe regarding how altruistic behavior is to be understood and accounted for. It is noted that whereas genetically related species exhibit a high degree of cooperation, the less genetic identity obtaining between individuals leads to a diminishment of cooperative activity [28].

#### Population genetics (A3i and A3ii)

Several authors used ‘genetic identity’ in relation to ethnicity or race. Definitions include ‘ancestry’ [29]; and ‘the ethnic-specific profile shaped upon Y-chromosome genes’ [30]. Other authors expressed concern with the prioritization of genetic constructs of ethnicity or race. A recurrent topic is that of Jewish identity, with a number of authors highlighting that ‘Jewishness’ is not based on ancestry alone. A number of articles used ‘genetic identity’ in regard to mathematic models of the probability of two individuals sharing specified DNA. These articles frequently defined their use of ‘genetic identity’, as ‘the fraction of the genome that is identical by descent’ [31]. ‘Genetic identity’ was used to describe or to study certain populations in this subcategory.

Current research dealing with population genomics and the origins of human populations raises several challenges to Native American identity based on a blend of scientific and legal attacks. This research places a heightened emphasis upon ‘genetic identity’ in accordance with contemporary scientific analysis, but in reality, this research constitutes a twenty-first century manifestation of a very old phenomenon in American social politics: the construction of race [32].

The legacy of race and ethnic nationalism bleeds into contemporary literature around the genetics of Jews. (…) disinterested sounding terms like ‘genetic identity’ presuppose specific notions of ancestry and racial purity. (…) Indeed, resonating with her discussion about the problems gender poses to ethnicity as a marker of ancient Jewishness, Baker draws attention to the significant presence of European women (at least 80%), incorporated into the community through conversion, marriage, and motherhood, in Ashkenazic Jewish genetics [33].

### Personal identity (Category B)

‘Genetic identity’ was often discussed in relation to personal and social identity. This category includes the subcategories of: personal and social identity; ‘disease’ identity; religious or spiritual identity; gender identity; critiques of genetic determinism or essentialism; external or environmental influences on identity; and human identity.

#### Personal and social identity (B1)

Articles in this subcategory discussed the impact of genetic knowledge, derived through genetic technologies, on one’s self-concept, social position and life. Definitions of ‘genetic identity’ in this subcategory include ‘that dimension of self-concept that develops from the individual’s perception of his or her inherited endowment’ [34]; and ‘one part of the lived experience of the genetic body’ [35]. A recurrent term in the reviewed literature that addressed these two identities is ‘biosociality’: a term that combines the biological body or biomedical knowledge with social relationships. Articles in this subcategory described the importance of social relationships in constructing ‘genetic identity’.

One way to engage with genetic knowledge as expressed in these two books is to consider the social and political context in which genetics are enacted as biosocial practices. Both studies illuminate that genetic identities are far from being centered on a stable bounded biological body, but rather concern a biosocial body surrounded by a plurality of genetic practices and discourses that are being reconfigured in social relations [36].

Biosociality recognises a central role for biomedical knowledge in constructing genetic identities and producing and reproducing social relationships. Accordingly, it is often imagined as a new form of social solidarity [37].

The more the study moves out from the individual subject to the family, the more important is the assumption that social identity–who we believe we are–coincides with genetic identity–who genetically we are [38].

#### ‘Disease’ identity (B2)

‘Genetic identity’ was also used in reference to genetic defects. Articles in this subcategory considered the significant consequences that (knowledge of) a genetic disease or predisposition can have on people’s sense of identity. These consequences were usually described in the context of genetic testing. Definitions of ‘genetic identity’ include ‘a shattered self-adequacy syndrome caused by the knowledge that one possesses a defective gene’ [39]; and ‘a function of defects to be corrected’ [40].

Individuals have to decide not only whether to incorporate their condition into their sense of themselves, but also how—with what moral valence (i.e., as positive or negative). They wrestle to gauge whether to view this genetic identity as negative or neutral—and to what degree to do so. A few saw themselves as “mutants,” “evolutionary errors,” “mistakes,” or “freaks of nature.” They felt that they had a ‘bad gene’ or ‘flaw,’ and struggled to understand it, stumbling at times in seeking appropriate terms. The fact that a mutation can be viewed as tainted can impede construction or embrace of a genetic identity [41].

The subcategory frequently overlaps with the population genetics subcategory described above, since ‘genetic identity’ was often discussed in regard to certain ethnic groups, due to a high prevalence of deleterious mutations in some populations, for example mutations in BRCA genes and Ashkenazi Jews. A perceived responsibility for genetic disorders was described in other articles and is particularly relevant in the context of prenatal testing and reproductive choices. Articles in this subcategory illustrated the use of ‘genetic identity’ concerning genetic disorders or predispositions and demonstrated their impact on one’s sense of identity, choices, and options in life.

Genetic accountability and genetic identity are now expanding beyond prenatal testing. Jewish women may perceive a ‘social obligation to do anything they [can] to advance’ research on BRCA1 testing. Accordingly, at least one researcher has warned that 'those obtaining consent for Jewish women [for BRCA1 testing] should be aware of the “slippery slope” from perceived social responsibility to coercion’ [42].

#### Religious and spiritual identity (B3)

In this subcategory the term ‘genetic identity’ was used to describe the relation between religion and ancestry. This subcategory illustrates the complex relationship between ‘genetic identity’, religious and ethnic identity and shows ‘genetic identity’ and religious or spiritual identity are both described as related and distinct concepts.

That is, blood relationship is part of the glory and mercy of God’s creation; genetic identity is a God-given principle, not a man-made one. (…) In particular, verses 5–6 create a complicated relationship between genetic identity and religious identity, and they seem to affirm an Islamic social structure based not just on faith but also on blood relationships [43].

‘This “half-Sikh identity”’, Wilson J concludes, ‘is a “genetic identity” which the mother was attempting to ‘re-write’. So, like Ward LJ’s judgment in Re P, the child in Re S is seemingly racially or ethnically Sikh through carrying his father’s ‘Sikh genes’ (as N was a Jew in Re P), but he can and should become a Muslim by faith, like his mother [44].

#### Gender identity (B4)

‘Genetic identity’ was sometimes used in relation to sex and gender identity and concerned the male (XY) or female (XX) chromosome pairs. This subcategory includes articles that discussed transsexuality or inter-sex disorders and ‘genetic identity’ in this context was used to indicate the ‘biological’ gender. This subcategory relates ‘genetic identity’ to the male or female identity.

In its sentencing judgment, the court set a requirement for Alkobi and others like him to declare at a certain point their ‘biological-genetic identity’ and to reveal the fact that they are ‘male’ or ‘female’ in the narrow sense currently accepted in society [45].

#### Critiques of genetic determinism or essentialism (B5)

Explicit criticism of the term ‘genetic identity’ and value judgments associated with the term were also encountered in the reviewed literature. This subcategory includes terms like genetic determinism, genetic essentialism, genism and ‘true’ identity. Articles in this subcategory described the dangers of overemphasizing one’s ‘genetic identity’ (leading to the loss of the functional family, genetic discrimination, loss of autonomy in genetic testing or reproductive choices) and stress a person is more than the sum of his or her genes. Authors often referred to differences between identical twins to illustrate their point and most expressed concern about the ease with which complex traits are increasingly attributed to genetics. Some authors described the dangers of overemphasizing one’s ‘genetic identity’ by referring to Gattaca, a 1997 film portraying a dystopic society driven by eugenics [46–48].

These are all illusions of genetic identity, based on extreme genetic determinism, and indeed should be a priori condemned and never permitted. First, because it is not true that two individuals with the same genetic make-up (twins) would have identical lives and behavior [49].

The interest in genetic identity includes a preoccupation with biological determinism. Among the traits attributed to genetics are mental illness, homosexuality, aggressive personality, dangerousness, job and educational success, exhibitionism, the tendency to commit arson, stress, risk-taking, shyness, social potency, traditionalism, and even zest for life. These complex conditions frequently are described as directly inherited, as if they were single-gene disorders [50].

#### External or environmental influences (B6)

Closely related to the above subcategory is the relation between ‘genetic identity’ and environmental contributions to one’s identity and phenotype. Some articles in this subcategory described (phenotypic) differences in genetically identical twins that could not be explained by their ‘genetic identity’. Also included in this subcategory were articles that described contributions by epigenetics, heritable phenotype changes that do not involve changes in DNA sequence [51]. This subcategory illustrates the relative significance of ‘genetic identity’ in relation to other contributions to identity and phenotype.

From the moment the organism's growth begins, differences in environmental factors will cause phenotypic divergence. (…) Further, even copies which received relatively similar intrauterine treatment may be subjected to different environmental conditions (nutrition, exercise, rest, etc.) after birth. Thus, initially identical genetic identity may nonetheless result in different phenotypic development [52].

Epigenetics does not displace the chromosome as the primary mechanism of biological inheritance. It does, however, challenge genetic determinism and the dichotomy between genes and environment. In the context of pregnancy and gestation, it challenges the dichotomy between form and matter, between genetic identity and the supportive environment of the womb [53].

#### Human identity (B7)

The last subcategory within the personal identity category concerns ‘genetic identity’ in relation to human species membership, often in discussion of the start of life and personhood (further discussed in category C), gene patenting (further discussed in category D) or possible effects on future human ‘genetic identity’ (also further discussed in category C). The relation between ‘genetic identity’ and human identity was described in somewhat different ways; definitions of ‘genetic identity’ in this subcategory include ‘determining one’s status as a member of the human family’ [54]; and something ‘at the core of each human being which reflects our overwhelming similarities, and distinguishes us (human earthlings) from other earthlings’ [55]; Other authors rather stated that human identity and ‘genetic identity’ couldn’t be equated. Although articles in this subcategory all connected ‘genetic identity’ with humanness or human identity, the extent of this connection varied.

Though human identity is not equivalent to genetic identity (some sociobiologists might differ on this point), the entire human genome encroaches far enough on fundamental human identity to make it unethical to patent, Brody contends. Patenting parts of the genome (genes or shorter DNA sequences) would remain under consideration, though. [56].

### (The beginning of) individuality (Category C)

This category concerns the right to a unique or own ‘genetic identity’ or autonomy over one’s ‘genetic identity’. It further includes articles that described the start of life, which includes discussions about embryo development and the start of ‘genetic identity’ and modification or selection of ‘genetic identity’.

#### Uniqueness and autonomy (C1)

This subcategory includes articles describing certain rights; the right to genetic uniqueness (a unique ‘genetic identity’), the right to a random ‘genetic identity’ (instead of something predetermined), the right to a non-manipulated ‘genetic identity’ and the right to individual modification. The right to *know* your ‘genetic identity’ in relation to parentage has already been discussed in the ancestry and heritage category (category A). Autonomy and freedom were most frequently discussed in relation to cloning and genetic modification. Authors usually asserted that one should be in control over one’s own ‘genetic identity’ and discussed the threats of cloning to autonomy and freedom. Some authors claimed that a unique ‘genetic identity’ is a right, whereas others refer to identical twins to deny this right exists and claim genetic uniqueness and *individuality* are two distinct concepts that should not be equated. Some authors argued that one has a right to a non-manipulated ‘genetic identity’ whereas others stated autonomy over ‘genetic identity’ includes a right to modification. In this subcategory, the various uses of ‘genetic identity’ in reference to autonomy and freedom contained many different rights and freedoms, both including the right to a non-manipulated ‘genetic identity’ and the right to genetic modification, particularly in relation to severe genetic disorders.

In addition, cloning is viewed as illicit because it ‘would impose on the resulting individual a predetermined genetic identity, subjecting him—as has been stated—to a form of biological slavery, from which it would be difficult to free himself’ [57].

Genetic identity is often confused with genetic uniqueness (the right to an exclusive genome). Genetic uniqueness cannot be a fundamental or basic human right because of the frequency with which it is violated without any harmful effects by the existence of monozygotic twins [23].

Years ago, the European Community appropriately included the right to a non-manipulated genetic identity in the list of civil rights [58].

The right to genetic identity, therefore, should both foresee the integrity but also the changeability of one’s genetic architecture: the right to personal identity may perfectly encompass the right to individual genetic modification [59].

#### Beginning of life (C2)

Articles in this subcategory discussed the significance of ‘genetic identity’ in determining when life (individuality or personhood) begins, and whether this was relevant for the protection of the embryo, often in the context of abortion or embryo research. A frequently quoted legal case from the USA is the Supreme Court landmark decision in Roe v. Wade from 1973. In this decision ‘genetic identity’ was not deemed sufficient to (legally) recognize the embryo as person. Some authors also mentioned German Constitutional Court abortion decisions. In these decisions, the first determination of ‘genetic identity’ was not discussed. However, the term was used as one criteria, in combination with others, to judge when an embryo was (legally) considered a person and therefore deemed protectable. They placed an emphasis on implantation and the formation of the primitive streak as the beginning of a life capable of protection, as opposed to the construction of ‘genetic identity’ at conception, which is also reflected in several moral-religious judgments on the subject. Most authors agreed that ‘genetic identity’ was first established at conception, although some stress that this is a process rather than a fixed moment. The articles in this subgroup used the term ‘genetic identity’ to refer to the combination of a sperm and ovum into stable chromosomes forming a new and distinct genome and they show a more or less uniform view of when ‘genetic identity’ is first established (at conception). However, this subcategory shows varied importance given to the term in relation to the beginning of life and the protectability of the unborn.

In Roe, the Court espoused the view that the unborn offspring of human parents, a human fetus, must be capable of ‘meaningful life’ outside the womb before he or she can be can be considered as having anything more than the ‘potential’ for human life. Genetic identity is, under this formulation, irrelevant, except to determine ‘potential’ for human life [60].

In its abortion decisions the Bundesverfassungsgericht skillfully avoided the question of when life begins, however it did emphasize that at least from the time of implantation there could be an individual, no longer divisible, life with its own genetic identity and that from this point the entity could be protected by both Article1 I GG and Article 2 II GG [61].

Fertilization marks genetic identity. Implantation, however, marks the development of biologic individuality, as well as the first emergence of the rudiments of the nervous system [62].

But it is clear that genetic identity is not necessarily fixed by the mere penetration of a single sperm, because more than one may enter, and because even then some genetic material may be rejected. In fact, it is only at the completion of the process of fertilization with syngamy, that is, when the genetic material of the sperm and the egg have condensed into chromosomes to form a new genotype–a single cell- that genetic uniqueness can possibly be fixed [63].

#### Modification, alteration or selection (C3)

This sub-category includes topics like cloning, gene therapy, genetic modification and pre-implantation diagnostics. Authors described the risks and consequences of these techniques and sometimes weighted these against possible benefits. As for cloning, risks frequently mentioned included threats to dignity, threats to autonomy and individuality and disturbed familial relationships. Threats to dignity were often described from a religious perspective and were combined with the need for genetic uniqueness and individuality. As for genetic modification, risks most frequently mentioned included effects on future generations, ‘designer children’, commodification of ‘genetic identity’ (further discussed in category D) and new forms of liability. The most often encountered benefit of these techniques was the treatment of serious diseases. This subcategory describes the modification or selection of a ‘genetic identity’ and its effects, both in relation to the individual and of the human population as a whole.

The natural genetic identity of the prenatal child may become important in the future. By manipulation of the biological makeup of the prenatal child, it may be possible to increase or decrease intelligence, to correct physical defects, or to produce those characteristics needed for strenuous or unique physical activity such as sports or space travel. While the failure to provide the child with superior biological equipment may not give rise to a new variation of the wrongful life type of litigation, the use of such techniques may create substantial risk of negligence or disastrous injury during the experimental stages [64].

However, the fear that gene therapy may have some impact on the genetic identity of the human species must be weighed against the benefit of providing treatment for common and catastrophic diseases [65].

### Privacy and property (Category D)

Category D involves the use of ‘genetic identity’ in the context of privacy and property. This category includes subcategories of: identification and protection of ‘genetic identity’; durability of ‘genetic identity’; and ownership and commercialization of ‘genetic identity’.

#### Identification and the protection of genetic information (D1)

‘Genetic identity’ was frequently used as an identifier in combination with the identification of a suspect in forensics or in combination with paternity testing. The term was used in reference to the need for privacy and protection of genetic information, for example in relation to DNA databanks, and protection from discrimination. Definitions of ‘genetic identity’ in this subcategory include something ‘used to identify the relevant person’ [66]; and something that ‘concerns identification (idem identity)’ [67]. The extent to which ‘genetic identity’ was considered proof of identity differed throughout the reviewed literature.

Instead, as a symptom of biology, relatives and future generations risk being stigmatized for their mere genetic similarity to a previously convicted defendant. Assuming possible criminality based on genetic identity replaces the wrongdoing requirement with what might be described as wrongbeing [68].

Even with the present technology, however, the donor has an interest in the content of the DNA analysis because DNA test results provide evidence of identity [69].

The proposed legislation seems to be based on the assumption that the ‘gene is the person’, that is, constitutive of genetic integrity while legislation on DNA forensics by the state relies on the myth that ‘gene denotes genetic identity’ [70].

#### Durability of ‘genetic identity’ (D2)

In a few articles, emphasis was placed on the durability of ‘genetic identity’. Articles in this subcategory all included the use of ‘genetic identity’ in DNA analysis, however some warned that the durability of genetic material might provide a false sense of reliability if little is known about the processing and storage of DNA in forensic cases.

DNA, for example, appears to be everlasting and immutable. But the durability of genetic identity supplies no confirmation that any particular DNA introduced at trial has been appropriately collected, analyzed, and preserved, nor whether the testimony regarding the DNA ‘match’ will be truthful or accurate [71].

#### Ownership of genetic identity and the commercialization of genetic information (D3)

This subcategory mostly comprises articles describing the risks of ‘genetic identity’ theft and articles discussing the patenting of genes and ‘genetic identity’. Other articles discussed the commodification or commercialization of genetic information. All articles in this subcategory discussed ‘genetic identity’ in reference to property rights, however the desirability of property rights of genetic material and identity is valued differently in the reviewed literature.

If a person does not own his genetic material, he can neither own nor protect his genetic identity, thus leaving it in peril indefinitely. If no one owns something, then, by legal definition, no one can be guilty of stealing it. How can we best protect our genetic identity? The only way to protect genetic identity from theft is to create a legal property right in the genetic information that secures this identity [72].

In her construction of genetic identity, ownership is resisted not only in the name of the human subjects who supply the genetic material but also in the name of all generations, past and future, who are implicated, and hence somehow present in the germline [73].

Judge Sweet’s trepidations mirror the public’s concerns about the gene-patent debate. The plaintiffs’ legal challenges to the BRCA patents raise a difficult legal and ethical dilemma: should information about an individual’s personal genetic identity be protectable as intellectual property for the purpose of promoting scientific innovation [74]?

### Regions of DNA (Category E)

Different regions of DNA were used in descriptions of ‘genetic identity’, including DNA base sequence, nuclear or mitochondrial DNA, the Y-chromosome, specific genes and the whole genome. The term was used in different and often contradictory ways, for example in reference to the inclusion or exclusion of mtDNA in determining ‘genetic identity’. Definitions in this subcategory include both the explicit restriction of ‘genetic identity’ ‘to sharing the same nuclear gene set’ [75]; and two individuals ‘having an identical mtDNA type’ [76]; but also the complete genome [77]. Other contradictory statements were found in the distinction between ‘genetic identity’ and *genomic* identity. This subcategory illustrates the use of ‘genetic identity’ as concerning certain genes, parts of DNA or the entire genome and it most strikingly reveals the different and contradictory uses of ‘genetic identity’.

Notably, the common feature of the definitions for permitted gametes and embryos, even in their extended form, is that deliberate changes to their nuclear DNA are explicitly disallowed. This distinction is not as tenuous as it seems, because mitochondrial DNA contain 37 genes and are passed through the maternal line, whilst nuclear DNA contains 20,000–25,000 genes which are inherited from both parents. Hence, the latter more strongly embodies an individual’s genetic identity and editing nuclear DNA can be considered ‘full-blown’ germline editing [78].

The increased knowledge concerning mtDNA obtained in this study is believed to offer a useful means of determining genetic identity due to increased mitochondrial DNA haplotype diversity, by allowing mtDNAs to be classified into several types of peak patterns [79].

Furthermore, genomic identity needs to be distinguished from genetic identity. In order for someone A to be genomically identical, the total genetic information in her or his cells needs to be the same at time t and time t’. The total genetic information in human cells comprises both the complex nuclear genome and the simple mitochondrial genome. If A is genetically identical, A is the same as regards a certain gene or certain genes (and not necessarily the whole genome) at time t and t’ [80].

[w]e might suggest that genetic identity concerns identification (idem identity), and that genomic identity ultimately connects with our individuality (ipse identity) [67].

#### Cells and genes (Category F)

In this category ‘genetic identity’ was mentioned in relation to specific human cells, genes or cell lines, mostly in articles from medicine or the life science (Fig 3). This category includes healthy and or diseased cells, enzymes, specific loci or defected genes. Definitions include the ‘promoter region of an endogenous gene that defines a neuronal subtype’ [81]; and ‘specific chromosome changes and by molecular changes related to the chromosome anomalies’ [82]. In the other articles in this category, the term ‘genetic identity’ was used but not further explained.

## Discussion

This article aimed to systematically identify and review the use of the term ‘genetic identity’. In only twenty-five out of the 616 articles that used the term, ‘genetic identity’ was defined or the use of the term was specified. Within the included articles, the term was used in various ways and with different and even contradicting meanings.

Content analysis revealed six categories of meaning: ancestry and heritage; personal identity; (the beginning of) individuality; privacy and property; regions of DNA; and cells and genes. The use of the term varied both between and within the six categories. Overall, the diversity in the use of ‘genetic identity’ in the reviewed literature demonstrates that the term is used differently in different contexts, but also within each context the meaning of the term can vary widely. There is also a lack of consistency within individual disciplinary discourses, as demonstrated by the term’s various use and understanding within the legal literature (e.g. in paternity cases, property law etc.) and life sciences (e.g. in population genetics, clinical genetics and cell biology). These findings support previous assertions that genetic and genomic identity are discussed in vague and contradictory ways in regulatory discourse and that this has important implications related to rights and protections associated with genetic knowledge [83]. How then might the current Clinical Trials Directive (or future Clinical Trials Regulation), prohibiting ‘clinical trials that result in modifications to the subject's germ line genetic identity’, be interpreted? Although authors often interpret the Clinical Trials Directive (and hence the term ‘genetic identity’) as prohibiting any changes at the germ line level [84–85], the need for a definition of the term has also been expressed [86–87]. The various aspects of the term, identified in this review, may help to clarify the discussion on the current and future regulation.

In the first place, a distinction can be made between biological and social aspects of ‘genetic identity’. The notion ‘genetic’ refers to a biological basis, whereas ‘identity’ is more frequently understood to have a wider connotation [88]. Authors have stressed that ‘genetic information’ is a part of, but does not equate to, personal identity [59], and described the risks of genetic determinism, attributing complex traits to genetics [50]. A recurrent term in the reviewed literature that addresses both biological and wider aspects is ‘biosociality’: a term that combines the biological body or biomedical knowledge with social relationships. The review has demonstrated that the term ‘genetic identity’, first coined by Sylvia Schild [89], a social worker, to describe ‘that dimension of self-concept that develops from the individual’s perception of his or her inherited endowment’, integrates biological and social elements. Although the distinction between biological and social aspects is not directly relevant for the current legislation, which focuses on biological interventions, it does show that issues of identity are not merely biological, but involve social interpretation. Decisions whether or not to allow for certain modifications are influenced by social and political views on human nature, which are not based on facts alone, but include values.

A second distinction is that between individual and collective aspects of the term “genetic identity”. Boussard explains how these dimensions could have different interests [90]; when the individual is protected as a depositary for the genetic heritage and diversity of the human species, it can be associated with the right to uniqueness, the principle of non-discrimination and the right to genetic integrity. These provisions protect the right to ‘genetic identity’ of present and future individuals and of the human species over time, and thereby limit scientific practices like germline gene modification, because they would affect future generations. However, when the individual is (artificially) separated from the human gene pool, the right to know one’s genetic constitution and the right to confidentiality become more prominent. On this level of ‘genetic identity’ protection, human rights encase the individual need for self-perception [90]. In the reviewed literature the right to ‘genetic identity’ was used to describe both the right to genetic integrity (originally termed in 1982 as ‘the right to a non-modified genetic heritage’) [91] and the right to individual genetic modification [59]. The central question that remains is if the prohibition in the Clinical Trials Directive and Regulation is intended to protect next generations because of a perceived inviolability of the human germline, or if it is focussed more on individual protection and rights (including freedom of procreation). Where the Oviedo Convention speaks of the protection of the human identity both as an individual and with regard to the human species [6], both the Clinical Trials Directive and Regulation seem to focus on the individual ‘subject’ or ‘human being’, and do not mention the human species or the protection of future generations in their provisions, although the relevant prohibition is limited to the *germline* ‘genetic identity’, which implies it concerns future generations in particular [8, 11].

Finally, a distinction can be made on the level of genes. The term ‘genetic identity’ may concern both the whole genome and only certain genes, and it may concern solely nuclear DNA as well as nuclear DNA in combination with mitochondrial DNA. This last distinction is also evident in current policy debate; in the UK for example, mitochondrial replacement techniques are treated more permissively than nuclear genetic modification, because they are deemed ‘unlikely to alter in significant ways the identity of the person created’ [92]. The global policy divergence around Mitochondrial Replacement Therapy (MRT) indicates regulatory uncertainty [93], and in this context a more clearly established definition of ‘genetic identity’ could clarify the European regulatory landscape. However, the ambiguity surrounding the term might provide the possibility to flexibly adapt practices in the light of new evidence and technologies.

Several strengths and limitations of this review need to be addressed. To our knowledge, this is the first systematic (scoping) review on the term ‘genetic identity’, and it comes at a significant time. The Clinical Trials Regulation is set to replace the current Clinical Trials Directive and scheduled to come into application soon, binding in its entirety in all Member States, including the prohibition of clinical trials ‘that result in modifications to the germ line genetic identity’ [11]. Furthermore, recent developments have led to techniques that could presumably facilitate germline modifications in humans. To understand the scope of the prohibition it is therefore crucial to know how the term ‘genetic identity’ is to be understood. The search of this review was performed in several databases, including the most relevant disciplinary perspectives (e.g. biomedicine, social sciences, humanities). However, no full-text access was obtained in 17% of articles, which undermines the completeness of this review. Finally, the analysis part of the review needs some clarification. Categories of content analysis were developed with the aim to be as mutually exclusive as possible. However, some overlap between categories was unavoidable. Furthermore, although the content analysis was performed by two reviewers, it involved interpretation by authors, which means some subjectivity was inevitable.

This review reveals the various and contradicting ways the term ‘genetic identity’ is used and understood. The implication for EU law (mainly the prohibition in the Clinical Trials Directive and Regulation) is that it is currently open to interpretation. Ambiguity in legal terminology is recognised as creating uncertainty and even potential loopholes.[94] Indeed, in Europe, national laws which are considered to be in line with the EU wide Clinical Trials Directive and Regulation range from the strictly prohibitive, such as the Dutch ‘Embryo Law’ (Embryowet) [95] which does not allow embryos to be made for research, to the more liberal, as is the case in the UK where mitochondrial replacement techniques are permissible [92]. Strict prohibition prevents investigation into the potential effectiveness and safety of germline genome editing and has real consequences for patients and their families. For example, women with mtDNA diseases and couples with cystic fibrosis who have a 100% recurrence risk are not allowed to use gene editing techniques. Differences in prohibitions both within and outside of Europe raise the possibility of medical tourism, now and in the future. Already, women with mtDNA disease sometimes consider going to the UK to take advantage of treatment possibilities unavailable to them in the Netherlands. In light of these policy and practice implications, clarity of meaning is needed from the European Medicines Agency in relation to the Clinical Trials Directive and Regulation. The importance of the Clinical Trials Directive cannot be overstated; although it may not carry the same moral weight as the Oviedo convention, it is legally more significant within a European setting. [7, 9–10] It should, therefore, reflect the values and concerns about gene editing technologies of European stakeholders. In order to decide upon the boundaries of what is or is not permissible and provide the kind of conceptual clarity required for proper regulatory implementation, further medical, legal, ethical and social debate would be required including a wide range of stakeholders. Such debate needs to look beyond more technical aspects of effectiveness and safety, to more far reaching societal implications, for example social justice considerations related to the potential for new forms of inequality. Indeed, the need for public consultation and co-responsibility for the normative framework governing gene editing technologies has been repeatedly recognised [96–99] and has gained new urgency following the birth of the first gene edited babies in China.[100] However, an appropriate public platform for debate has yet to manifest. The World Health Organisation’s new advisory committee to develop global standards for the governance and oversight of human genome editing,[101] whilst representing different countries and disciplinary perspectives, is an official body with a closed process and does not represent the kind of more broad, democratic discussion that has been repeatedly advocated for [96–99]. An inclusive and democratic platform is needed specifically at a European level to ensure Europe-wide regulations governing gene editing technologies reflect European values and concerns, but also to ensure consistency in the intention and wording of regulations and guidelines across other relevant European legal frameworks and programmes (e.g. the European legal framework related to patents, [102] the European regulation on Genetically Modified Organisms, [103] and the European Commission’s research funding programmes, responsible for the ethics guidelines informing EU funded research [104]). Furthermore, due to the potential for medical tourism, European interests should be represented in an international body such as the United Nations [96] or a Global Observatory [97] in a coordinated global response. In conclusion, because of the diversity of meaning with which ‘genetic identity’ is used and understood, further reflection is needed. This requires medical, legal, ethical and social debate and a coordinated response at both a European and a global level.

## Supporting information

S1 Appendix Detailed search strategy. (DOCX)

S2 Appendix Data extraction table. [available at: https://doi.org/10.5281/zenodo.2611190]. (DOCX)

S3 Appendix PRISMA-ScR Fillable checklist. (DOCX)
