# Supplementary material for: Systematic scoping review of the concept of ‘genetic identity’ and its relevance for germline modification
Source: PLoS One. 2020 Jan 24;15(1):e0228263. doi: 10.1371/journal.pone.0228263 (PMC6980575; doi:10.1371/journal.pone.0228263)
Supplement: S1 Appendix — (DOCX) [file pone.0228263.s001.docx]

***S1. Appendix. Detailed search strategy***

Database: PubMed Session Results (March 1, 2018)

Results: 560 items

Search terms: genetic identit*[tiab] NOT (‘Animals’[Mesh] NOT ‘Humans’[Mesh])

Database: Embase Session Results (March 1, 2018)

Results: 573 items

Search terms: 'genetic identit*':ab,ti,kw NOT ([animals]/lim NOT [humans]/lim)

Database: IBSS Session Results (March 1, 2018)

Results: 32 items

Search terms: ti(‘genetic identit*’) OR ab(‘genetic identit*’)

Database: ATLA Religion Session Results (March 1, 2018)

Results: 57 items

Search terms: TX(‘genetic identit*’)

Database: PsycINFO Session Results (March 1, 2018)

Results: 34 items

Search terms: (TI(‘genetic identit*’) OR AB(‘genetic identit*’)) NOT (PO Animal NOT PO Human)

Database: CINAHL Session Results (March 1, 2018)

Results: 23 items

Search terms: (TI(‘genetic identit*’) OR AB(‘genetic identit*’)) NOT (MH ‘Animals’ NOT MH ‘Human’)

Database: Scopus Session Results (March 1, 2018)

Results: 1,993 items

Search terms: TITLE-ABS-KEY (‘genetic identit*’)

Database: HEINonline (March 7, 2018)

Results: 758 items

Search terms: ‘genetic identity’ OR ‘genetic identities’ [Law Journal Library]

Database: Web of science (March 7, 2018)

Results: 1,592 items

Search terms: [Topic] ‘genetic identity*’

Database: JSTOR (March 7, 2018)

Results: 47 items

Search terms: ‘genetic identity’ OR ‘genetic identities’ [Religion]

Database: The Philosopher’s Index (March 7, 2018)

Results: 13 items

Search terms: ‘genetic identity’ OR ‘genetic identities’
