# Supplementary material for: Systematic scoping review of the concept of ‘genetic identity’ and its relevance for germline modification
Source: PLoS One. 2020 Jan 24;15(1):e0228263. doi: 10.1371/journal.pone.0228263 (PMC6980575; doi:10.1371/journal.pone.0228263)
Supplement: S2 Appendix — (DOCX) [file pone.0228263.s002.docx]

***Appendix 2 –Data extraction table and references***

| **Author (s), Year, Reference** | **Location** | **Discipline** | **Context, value** | **Defined or undefined** | **Code** |
| --- | --- | --- | --- | --- | --- |
| Abbing, H.D.C. (1998) [1] | 2. Netherlands | Law (1) | ‘It explicitly restricts genetic identity to sharing the same nuclear gene set, meaning any intervention by embryo splitting or nuclear transfer techniques seeking to create a ‘human being’ in the sense as indicated above. | Defined | A2i  E2  C3 |
| Abrams K. & Garett , B.L. (2016) [2] | 1. USA | Law (1) | ‘Our focus in this Article is on genetic identity used to identify the relevant person and not in the sense of identifying a person's phenotype or genetic characteristics.’ | Defined | D1 |
| Abrams, K. & Piacenti, R.K. (2014) [3] | 1. USA | Law (1) | ‘In those cases where there is a dispute about genetic identity, marriage, genetics, and functional parenthood compete for primacy and states have come to different conclusions regarding how to weigh their importance.’ | Undefined | A1 |
| Abuissa, H. et al. (2005) [4] | 1. Canada | Medicine (4) | ‘Realigning our 21st century diet and lifestyle with our hunter-gatherer genetic identity’ | Undefined | A2ii |
| Adams, K.E. (2003) [5] | 1. USA | Medicine (4) | ‘The creation of a donor child, or having a child to save another child, has met with criticism that dates back to the 1990 Ayala case, in which the father had a vasectomy reversed after 17 years so he and his wife could conceive a child in hopes of creating a marrow donor for their 18-year-old daughter who was ill with leukemia. In such a scenario there truly is no unconflicted decision-maker, no one who simply represents the interest of the child to be desired and appreciated regardless of his genetic identity.’ | Undefined | C3 |
| Aglaguel, A. et al. (2017) [6] | 7. Morocco | Biosciences (4) | ‘(…) Poikiloderma with Neutropenia (PN) is inherited genodermatosis which results from a biallelic mutation in the USB1 gene (U Six Biogenesis 1). (…) We identified two undescribed homozygous mutations in the USB1 gene: c.609 + 1G>A in two siblings and c.518 T>G(p.(Leu173Arg)) in the other case. Conclusion This report confirms the clinical and genetic identity of Poikiloderma with Neutropenia syndrome.’ | Undefined | F1 |
| Ahmadi, M. & Ahmadi, L. (2013) [7] | 5. Iran | Law (1) | ‘Germ line gene therapy on the other hand operates on the reproductive cells and transmits the changes to the person’s future offspring’s thus changing the genetic identity of futures generations.’ | Undefined | C3  E5 |
| Ajunwa, I. (2014) [8] | 1. USA | Law (1) | ‘The practice of gene theft … opens the door to the negligent disclosure of an individual’s private and potentially damaging genetic information, and leaves the individual vulnerable to the menace of genetic identity theft.’ | Undefined | D1  D3 |
| Alexander, F. S. (1985) [9] | 1. USA | Law (1) | ‘Within the last few years, however, it has become possible to achieve a complete genetic identity with the contracting couple through the transplantation of a fertilized ovum to the third party.’ | Undefined | A1 |
| Alfano, J.A. (2005) [10] | 1. USA | Law (1) | ‘ The court observed that although saliva contains ‘a significant amount of genetic identity information’ which the suspect may understandably expect to be private, ‘expectorating is not ... concealed behind closed doors as urinating.’ | Undefined | D1 |
| Aloni, E. (2011) [11] | 1. USA | Law (1) | ‘Despite their shared genetic identity, clones will not be identical in physical or behavioral characteristics, because DNA is not the sole determinant of appearance or personality.’ | Undefined | A2i  B1  C3 |
| Alpa, G. (1997) [12] | 3. Italy | Law (1) | ‘The first reason is that-even in the context of a general right of personality-several forms of right can be discerned which resemble, or assist, or are actually intertwined with the right to privacy. I refer to the following: (…) (iv) The right to a person’s genetic identity.’ | Undefined | D1 |
| Alpert , R.T. (2007) [13] | 1. USA | Religion (3) | ‘As science becomes more comfortable with the idea that ‘nature’ and ‘nurture’ interact to make us who we are, so we Jews are beginning to accommodate ourselves to understanding the ways in which our genetic and social identities interact to define who we are.’  ‘*Jewishness is not determined by genetics. Nonetheless, genetic threads run through Jewish populations that [provide] them with a group identity. This genetic identity has been retained and modified, much as the religious and cultural identity of Jews has been retained and modified over more than two millennia (Ostrer, 897).’* | Undefined | B1  A3i |
| Alsgaard, H. (2012) [14] | 1. USA | Law (1) | ‘Early in the history of reproductive technology use, ‘[p]arents rarely told their children that they were conceived with donated sperm; many psychologists counseled parents to protect themselves and their child from the possibility that the child might feel resentment if she learned that she was ‘different’ from other children.’ Certainly, not all parents hide the genetic identity of their children, and as more children become aware of their genetic history, more internet identification and connection sites have opened.’ | Undefined | A1 |
| Alvare, H.M. (2003) [15] | 1. USA | Law (1) | ‘Collaborative reproduction also intimately affects children’s well-being: it affects children’s genetic identities, as well as their physical and emotional health. Expert observers of collaborative reproduction, even those generally favoring use of ARTs, have concluded that existing law and practices are driven primarily by adult desires rather than children’s needs.’ | Undefined | C3 |
| Almer, M.S. (1996) [16] | 1. USA | Law (1) | ‘While courts generally agree that the embryos created from IVF belong to the parents, no one has considered whether the children created from this procedure, once born, have any property rights over the other cloned embryos by virtue of their shared genetic identity.’ | Undefined | D3  A2i  C3 |
| Anca, M.H. et al. (2004) [17] | 5. Israel | Medicine (4) | ‘This is the first report of genetic identity associated with different age of disease onset as well as a different motor and behavioral phenotype. Postzygotic events are a likely explanation for the observed differences of phenotype in these genetically identical twins.’ | Undefined | A2i  B6 |
| Anderlik, M.R. & Rothstein, M.A. (2002) [18] | 1. USA | Law (1) | ‘More generally, it is easy to slide from an appreciation of the relevance of genetic identity to healthcare, given the current state of technology, to a belief that genetic identity is one’s ‘true’ identity and genetic relationships are more fundamental, enduring or ‘real’ than other kinds of relationships. | Undefined | B5  A2ii  B1 |
| Anderson, M.L. (2006) [19] | 1. USA | Law (1) | ‘The lack of regulation will also create a generation of children born from these procedures who will be unable to fulfill the compelling psychological need to know their complete genetic identity’ | Undefined | A1 |
| Andrews, L.B. (1998) [20] | 1. USA | Law (1) | ‘Cloning may also have negative impacts on broader legal concepts. Pizzulli points out that: a) privacy and autonomy might be severely attenuated in one known by himself or others to have a predetermined genetic identity;’ | Undefined | D1  C1  C3 |
| Andrieu, J. et al. (2002) [21] | 1. USA | Law (1) | ‘… to grant exclusive rights to the use and sale of an individual’s entire genome promotes commercial exploitation and exchange of a person’s unique genetic identity. This is a particularly troubling negation of that individual' s -Defendant Dolly' s- right to bodily autonomy.’ | Undefined | D3  E6  C1 |
| Annas, G.J. (2009) [22] | 1. USA | Law (1) | ‘Preventing genism from taking over where racism left off by substituting molecular differences for skin color differences will not be easy. Two actions, however, seem necessary. First, genetic privacy must be protected. No one’s genes should be analyzed without express authorization, and, of course, no ‘genetic identity cards’ should be permitted. Second, pseudoscientific projects that purport to identify genetic differences between ‘races’ should be rejected.’ | Undefined | D1  B5 |
| Archard, D. (1993) [23] | 2. Ireland | Philosophy (3) | ‘The entitlements to ownership of the children generated by reproductive labour also meet the Lockean proviso. The child conceived by a particular couple could not have come into existence but for their sexual congress, and its unique genetic identity is solely attributable to their powers and nature.’ | Undefined | D3  A1 |
| Armstrong, D. et al. (1998) [24] | 2. UK | Medicine (4) | ‘This paper describes a qualitative study of the processes employed within a genetic counselling consultation. These processes, it is argued, can be seen to provide the patient with a genetic identity. But unlike the new identity conferred by the diagnosis of many chronic medical conditions, a genetic identity is presented as an old one that is now revealed.’ ‘But genetic medicine both reasserts the pathological model and extends it to community surveillance, while at the same time it removes the problem of identity from the purview of psychology and the other ``human sciences'' to the new genetics in a process that can be described as ``geneticisation'' (Lipmann, 1991). This process, reproduced in the structuring of the consultation, entangles the individual in the genetic identity of others. Patients are at once incriminated within lines of inheritance and have the responsibility of that problem dissipated in the web of consanguineous relations that they share. Despite its name, the genetic counselling consultation is an important mechanism in the process of revealing and consolidating an identity in a web of genetic connectedness.’ | Undefined | B2  A2ii |
| Attanasio, J.B. (1988) [25] | 1. USA | Law (1) | ‘Much of the chapter explores how courts have struggled to reconcile the conflicting desires of the birth parents for privacy with the child’s need to know her genetic identity.’ | Undefined | A1  D1 |
| Atwill, N. (2000) [26] | 1. USA | Law (1) | ‘Although complete genetic identity of two human beings does not lead to psychic similarity, these human beings would be still seen as identical copies of each other and of the cloned individual of which they would be truly a copy.’ | Undefined | A2i  B6  C3 |
| Atzmon, G. et al. (2010) [27] | 1. USA | Medicine (4) | ‘However, these and successor studies of monoallelic Y chromosomal and mitochondrial genetic markers did not resolve the issues of within and between-group Jewish genetic identity.’ | Undefined | E4  E3  A3i |
| Bady, P. et al. (2012) [28] | 2. Switzerland | Biology and Genetics (4) | ‘Glioma cell lines are an important tool for research in basic and translational neuro-oncology. Documentation of their genetic identity has become a requirement for scientific journals and grant applications to exclude cross-contamination and misidentification that lead to misinterpretation of results.’ | Undefined | F1 |
| Bahri, R. et al. (2014) [29] | 7. Tunisia | Anthropology (2) | ‘In spite of the population movements suggested between the two shores of the Mediterranean, and between the Middle East and the South Mediterranean, these regions have retained their genetic identity.’ | Undefined | A3i |
| Baker, K.K. (2016) [30] | 1. USA | Law (1) | ‘Being raised by genetic parents does help ensure that children have knowledge of genetic information that could affect their own medical treatment, and it may play a role in children's sense of their own identity. But genetic science will likely soon render the former inconsequential, and the importance of genetic identity to psychological health is much debated.’ | Undefined | A1 |
| Baker, T.S. (1987) [31] | 2. UK | Law (1) | ‘Who is the mother? Is it the woman who carried the child for nine months and gave birth, or is it the woman who provided the egg and therefore gave the child its genetic identity?’ | Undefined | A1 |
| Bangert, B.C. (1979) [32] | 1. USA | Religion (3) | ‘Genetic identity exists from the time of conception. From floating blastocyst to implanted embryo to a fetus… the organism possesses an entelechy, or a pattern of development, which demands the conclusion that this life is anything but a mere tissue or appendage of a woman’s body.’ | Undefined | C2 |
| Barasoain, M. et al. (2011) [33] | 3. Spain | Genetics (4) | ‘Some studies suggest that stable markers ... and the study of populations with *genetic identity*, could provide a distinct advance to investigate the origin of CGG repeat instability.’  ‘According to Cavalli-Sforza & Piazza (1993), ‘*conservation of a distinct language must have been an important factor in maintaining social and genetic identity*.’’ | Undefined | A3i  B1 |
| Barnes, E.B. (2002) [34] | 1. USA | Theology (3) | ‘Unable to walk … Eugene sells his body fluids, hair, and skin cells to Vincent who assumes Eugene’s superior genetic identity and with it his name and social identity…’ | Undefined | B5  D3  D1 |
| Barnett, D.L. (1985) [35] | 1. USA | Law (1) | ‘Although the surrogate mother and the IVF-carrier situations are similar, they are distinguishable on the basis of genetic identity. A surrogate mother is related to the child biologically because she is the genetic mother of the child she is carrying. The IVF carrier, on the other hand, does not have the same biological relationship to the child since the husband and wife are the genetic parents.’ | Undefined | A1 |
| Baron, P.D. (2006) [36] | 4. New Zealand | Law (1) | ‘… there is a natural link between genetic fathering and legal parenting. In other jurisdictions, the increasing emphasis upon genetic identity, the ready availability of DNA paternity testing, the failure of marriage as a securer of paternity and the rhetoric of ‘family values’ have reinforced the idea of family as genetically determined.’ | Undefined | A1 |
| Basset, U.C. (2012) [37] | 6. Argentina | Law (1) | ‘The concept of the blood tie grew out of an entanglement of marriage and birth more than out of the more contemporary idea of biological or even genetic identity. The remote biological component was presupposed in marriage. Thus society disregarded adulterous children and, in general, children born out of wedlock.’ | Undefined | A1 |
| Basu, D. et al. (2016) [38] | 1. USA | Biology (4) | ‘Nevomelanocytic cells from tumors of the 4 NCM patients were isolated following previously described procedures. (…). Cells grown in vitro from the patients’ tumors showed the same mutation, confirming their genetic identity. P01S was positive for NRAS p.Q61R and all others were positive for NRAS p.Q61K (Table 1).’ | Undefined | F1 |
| Beadle, L. (2004) [39] | 4. New Zealand | Law (1) | ‘The commercialization of such entities is regarded as morally concerning per se, but is also seen as objectionable as, if a market of buying and selling human embryos was to emerge, it would almost certainly classify such embryos by genetic identity and other measures of utility.’ | Undefined | D3 |
| Becker, S.W. (2009) [40] | 1. USA | Law (1) | ‘That is, the defendant's request was not merely for the purpose of impeaching the prosecution's evidence; ‘[i]nstead, he [sought] to present, for the first time, evidence about the genetic identity of Payne's as- assailant.’’ | Undefined | D1 |
| Beh, H.G. & Diamond, M. (2000) [41] | 1. Hawaii | Law (1) | ‘Upon discovery ‘that the girl has an XY genotype, a genetic abnormality called testicular feminization’ and ‘precancerous testes that require surgical removal’ and will need vaginal surgery to have intercourse, the question arose whether the child or the parents should be told the genetic information or the fact that she is ‘'really a guy.'‘ (…) The authors conclude that if ‘the functions of guardians to secure the wishes and welfare of minors ... [cannot] be secured by disclosing [the patient’s] genetic identity to her parents, then there seems no sound ethical reason to disclose this information in these circumstances.’ | Undefined | B4  D1 |
| Bell, D. (1999) [42] | 4. Australia | Law (1) | The report of the French Council … says the notion that genetic identity equates with personal identity ‘is devoid of any scientific foundation’. The United States National Bioethics Commission’s report also distances itself from any mechanistic identification between the human genome and the human psyche.’ ‘The Committee argues that while genetic identity does not equal personal or psychic identity, nevertheless certain things which flow from genetic uniqueness (for example, distinctive ‘appearance of body and countenance’) are culturally valued.’  ‘It is a limited conception of human rights which, in trying to ascribe dignity to human beings, eviscerates the human subject of the capacity for self-definition and self-determination in the name of the protection of a subject which is defined by its own genetic identity.’ | Undefined | B1  B5  C3 |
| Bennett, B. (2009) [43] | 4. Australia | Law (1) | ‘It is … through our genes that are not ours alone, but also form part of our familial story, past and present, forming the biological bonds that join us to others, structuring the family and defining – at least in part – who we are and who our children will be. Through all of these ways, genetics configures us as inherently relational. (…) ‘*Genetic identity is thus revealed and established within a web of genetic connectedness overlaid upon a web of family bonds and family memories, with the burden of mutual obligations and caring commitments, and with all the ethical dilemmas they entail.’* | Undefined | A2ii  B1 |
| Bently, L. & Sherman, B. (1995) [44] | 2. UK | Law (1) | ‘Whereas, even if it were possible to obtain a patent for modifying genetic identity, for example, a process which would enable a modification of the human genetic code to be controlled in connection with in vitro fertilization intended to correct certain genetic deficiencies, such a process should be compatible with the dignity of man.’ | Undefined | D3  C3 |
| Berg, K.D. & Murphy, K.M. (2003) [45] | 1. USA | Medicine (4) | ‘Human genetic identity testing is a highly useful means to discriminate between individuals in forensic, paternity, and medical testing. The application of the polymerase chain reaction to genetic identity testing has facilitated the spread of this type of analysis into the realms of quality control and quality assurance of human tissues being assessed for pathologic processes.’ | Undefined | D1 |
| Bergeron, J.H. et al. (2002) [46] | 2. UK | Law (1) | ‘Freedom in the development of personality is enhanced by establishing a right to the protection of health and genetic identity and requiring legislative initiatives to protect each person from biomedical interventions.’ | Undefined | C1 |
| Black, J. (1998) [47] | 2. UK | Law (1) | ‘To what extent can a person be said to have a right to an individual genetic identity, particularly given that the experience of genetically identical twins suggests that a unique genetic identity is not essential for a human being to feel and to be an individual?’ | Undefined | C1 |
| Blake, V.K. (2013) [48] | 1. USA | Law (1) | ‘Parental rights of gamete donors come under a number of arguments including genetic-identity argument (that genetic relation takes precedence) as well as property rights over gametes.’ | Undefined | A1 D3 |
| Blyth, E. (1995) [49] | 2. UK | Social Work (2) | ‘Interviews with centre staff provided limited information about donor recruitment and selection practices and their implications for the welfare of the child. (…) However (…) given that donor offspring are not to be granted access to information identifying the donor, the HFEA and centres need to recognise that the information provided by the donor at the point of recruitment represents one half of the donor offspring's genetic identity. (…) Future practices concerning the collection of such information should be influenced by the wishes of donor offspring, such as those cited earlier in this paper.’ | Undefined | A1  D1 |
| Blyth, E. (2010) [50] | 2. UK | Social Work (2) | ‘This paper outlines these developments and discusses continuing challenges as regards registration of births following a Parental Order and arrangements for ensuring that individuals subject to a surrogacy arrangement are able to learn the circumstances of their conception and access information regarding their biographical and genetic identity.’ | Undefined | A1 |
| Blyth, E. & Farrand, A. (2004) [51] | 2. UK | Social Work (2) | ‘On the other hand, Steve Ramsey, Director of South Australia's Office of Families and Children, has stated: ‘*principles from the domain of human rights can provide an important framework for responding to one of the most pressing challenges confronting reproductive technology, and that is access by donor offspring to information about their origins...Put at its most succinct, from a human rights perspective, one might ask the question - how can one argue against the basic human right to know one’s own genetic identity*. ‘‘ | Undefined | A1 |
| Blyth, E. & Frith, L. (2008) [52] | 2. UK | Social Work (2) | ‘Sixty-three per cent of the heterosexual couples and 98 per cent of the lesbian couples had chosen an identifiable donor and most of these couples stated their belief that their child had a right to know his or her genetic identity.’ | Undefined | A1 |
| Bordet, S. et al. (2007) [53] | 1. Canada | Law (1) | ‘Human individuality and identity are also concerns identified in international instruments, although they are ordinarily mentioned to underline the contribution of factors other than genetics to individuality and to prohibit discrimination against persons on the basis of their genetic identity. They are not discussed in the context of animal-human combinations.’ | Undefined | B1  D1  B6 |
| Borecki, I.B. (1998) [54] | 1. USA | Biostatistics (4) | ‘The body-mass index (BMI) and fat mass (FM), measured by underwater weighing, were assessed for 1,630 individuals from ∼300 families from phase 1 of the Quebec Family Study. The high degree of genetic identity between the two traits is not surprising, since the BMI often is used as a surrogate for FM; however, simultaneous analysis of both phenotypes enabled the detection of a second major locus, which apparently does not affect extreme overweight (as does the primary major locus) but which affects variation in the ‘normal’ range.’ | Undefined | F1 |
| Borowski, C.M. (1999) [55] | 1. USA | Law (1) | ‘It could be argued by the proponents of human cloning that they have a fundamental right to their genetic identity and therefore have the right to copy it if they choose, while opponents of human cloning could counterargue that the clone ultimately has the fundamental right to have her own unique identity and genetic makeup.’ | Undefined | D3 C1  C3 |
| Bozzato, G. (2010) [56] | 3. Italy | Bioethics (3) | The genetic identity-individuality is the embryo’s ‘genetic form,’ which corresponds to the ‘static’ (synchronic) structural order of its DNA. This is an unchangeable molecular form throughout the whole spectrum of embryo development till the end of its individual adult life cycle. The genetic form is given by the sequence of bases of which each gene is constituted and by the genes’ arrangement along the DNA molecule. It expresses the ‘genome or the genetic information,’ otherwise named ‘informational content … which determines the structure (‘molecular form’ of DNA).’’ ‘… the genetic identity is the DNA molecule, i.e., the arrangement of genes … Biological identity is the DNA expression (expression of genes) and the interaction among gene products over time…’  ‘For all these reasons, to unmistakably characterize the biological (individual, personal) history of each human individual and to ontologically identify him from the first instant of his existence on is not determined by the ‘static’ structural order of his DNA. Rather this biological history derives from the dynamic genetic organization of his DNA (its scheme of the organization of gene expression of DNA). Biological identity (which includes the genetic identity), of which somatic (corporal) identity is the fruit (visible on optical microscopy as the primitive streak only from the fourteenth day on), is really singular for each human individual.’ | Defined | C2  E1  E6 |
| Bradley, G.V. (1997) [57] | 1. USA | Law (1) | ‘Racial discrimination is generally wrong because it is almost always unfair. Why is it unfair? Because race (and skin color, genetic identity) is not a character trait.’ | Undefined | A3i |
| Brannigan, M.C. (1989) [58] | Unknown | Ethics (3) | ‘The painful question of genetic identity and its complexity is evidenced by the nomenclature advanced by Snowden and Mitchell. They contend that there are actually seven distinct roles for the female: genetic mother, carrying mother, nurturing mother, genetic-carrying mother, genetic-nurturing mother, carryingnurturing mother, and complete mother.’ | Undefined | A1  B1 |
| Brodeala, E. (2016) [59] | 3. Italy | Law (1) | ‘Referring to the interpretation of Article 8 given in these cases, the Court of Appeal concluded that if it did not recognize the genetic parents as legal parents, it would unjustifiably disregard the children’s best interest and their right to know their real genetic identity.’ | Undefined | A1 |
| Brodie, D. W. (1970) [60] | 1. USA | Law (1) | ‘The natural genetic identity of the prenatal child may become important in the future. By manipulation of the biological makeup of the prenatal child, it may be possible to increase or decrease intelligence, to correct physical defects, or to produce those characteristics needed for strenuous or unique physical activity such as sports or space travel. While the failure to provide the child with superior biological equipment may not give rise to a new variation of the wrongful life type of litigation, the use of such techniques may create substantial risk of negligence or disastrous injury during the experimental stages.’ | Undefined | C3 |
| Brown, M.E. et al. (2010) [61] | 1. USA | Cellular and molecular pathology (4) | ‘Induced pluripotent stem cells (iPSCs) hold enormous potential for the development of personalized in vitro disease models, genomic health analyses, and autologous cell therapy. Here we describe the generation of T lymphocyte-derived iPSCs from small, clinically advantageous volumes of non-mobilized peripheral blood. These T-cell derived iPSCs (‘‘TiPS’’) retain a normal karyotype and genetic identity to the donor.’ | Undefined | F1  A2i |
| Brownsword, R. & Somsen, H. (2009) [62] | 2. UK | Law (1) | ‘Applying these general observations to the field of human genetics, we might suggest that genetic identity concerns identification (idem identity), and that genomic identity ultimately connects with our individuality (ipse identity). Genetics generates reliable knowledge (because it is derived from few data), on the basis of which we may identify persons; genomics generates unreliable and ambiguous knowledge (because it is based on imperfect understandings of numerous complex inter- relationships) about our individualities.’ | Defined | D1 |
| Brumnik, R. & Podbregar, I. (2010) [63] | 3. Slovenia | Criminal, Justice, Security (1) | ‘In the light of the above analysis, CCNE (2007) recommends: (…) placing genetic identity databases under the authority of an independent judge, assisted if necessary by other judges.’ | Undefined | D1 |
| Brunk, C.G. (1988) [64] | 1. Canada | Philosophy, Religion (3) | ‘Voth says that the practice of cloning people represents a ‘grotesque travesty of man … and human reproduction,’ (…) Certainly he does not want to say that genetic identity itself is a grotesque distortion of the divine image in humanity, since to do so would be to say this about all identical twins. Rather, he must mean that there is something in this means of producing genetic identity that threatens the dignity of persons as well as the character of our community.’ | Undefined | A2i  C3 |
| Buckle, S. et al. (1989) [65] | 4. Australia | Philosophy (3) | ‘This is the argument from genetic uniqueness. It can be argued that the significance of the moment when the sperm passes into the egg is that this is when the unique genetic identity of the child-to-be is determined.’ ‘If there are just a few cases in which another sperm will subsequently be incorporated, and genetic identity will only be fixed at a later stage, then it can never be said with certainty that genetic identity is fixed at the moment the first sperm passes through the zona pellucida.’ ‘We would then either have to take this moment as the beginning of a new human life, or conclude that the determination of genetic identity is not, after all, a sufficient ground for holding that a new human life has begun.’ | Undefined | C2 |
| Bugert, P. et al. (2012) [66] | 2. Germany | Medicine (4) | ‘In paternity testing ABO genotyping is superior to ABO phenotyping with regard to PL and PE, however, ABO genotyping is not sufficient for valid paternity testing. Due to the much lower mutation rate compared to STR markers, blood group SNPs in addition to anonymous SNPs could be considered for future kinship analysis and genetic identity testing.’ | Undefined | D1 |
| Cahill, L.S. (1999) [67] | 1. USA | Theology (3) | ‘To return to the case of the ‘sibling’ clone (to replicate a living or dead child, or to supply body products for an ill sibling), the issue of genetic identity within greatly unequal power positions in the family might be diminished, but the objectification or end-means aspect would certainly become more pronounced.’ | Undefined | A2i  C3 |
| Cahn, N. (2012) [68] | 1. USA | Law (1) | ‘Finally, such families raise the danger of overemphasizing one’s genetic identity at the expense of the functional family, or ‘genetic essentialism,’ the concept that a person is the sum of her genes.’ | Undefined | B1  B5 |
| Cahn, N. & Singer, J. (1999) [69] | 1. USA | Law (1) | ‘Unsealing birth records allows adoptees to find their biological relatives. Such a focus raises the danger of overemphasizing one’s genetic identity (…) books and articles written for adoptees stress the importance of finding one’s natural or birth parents and suggest that knowing one’s genetic heritage is a way to define identity. These books and articles are part of a trend, which Dreyfuss and Nelkin define as ‘genetic essentialism,’ the concept that a person is the sum of her genes and that behavior can be predicted based on genetic information.’ | Undefined | B5  A1  B1 |
| Callahan, S.C. (1995) [70] | 1. USA | Psychology (2) | ‘In our society the ongoing abortion debate and the new debate over whether to use aborted fetal tissue for research also centers on whether we accept the embryo as morally equal to other developing stages of the human lifecycle. Those like myself, who take genetic identity as determining one’s status as a member of the human family, affirm a radically inclusive moral stance of equality for embryonic life – and demand equal protection for these most vulnerable of all lives.’ | Defined | B7  C2 |
| Callus, T. (2004) [71] | 2. UK | Law (1) | ‘Indeed, the case of genetic identity of donor offspring highlights the need for the court to embark upon an appreciation of the relevant interests at stake: those of the offspring, the donor, the social parents and any other members of the wider genetic family. If the circumstances surrounding anonymous birth allowed the Court to evade this task by relying on the superior right of respect for life, it is evident that the exercise of the right to access of one’s genetic information will not compete with any superior right, but rather with equal rights of others.’ | Undefined | A1 |
| Campbell, R.B. (1991) [72] | 1. USA | Mathematics (5) | ‘Inbreeding coefficients genetics (or coefficients of consanguinity) measure the genetic identity between two individuals. They are discussed in all standard population genetics texts (e.g., [S], [6]). Although concise formulas that employ genetic identity as measured by inbreeding coefficients are available, many caveats limit their utility.’ | Undefined | A3ii |
| Campbell, R.B. (2015) [73] | 1. USA | Mathematics (5) | ‘Our question is what is the expected time since two alleles in an individual in generation 0 had a common genetic ancestor. We shall consider the top left individual in Fig. 2: one of its alleles came from one parent and the other from its other parent in generation 1. The possible locations of the ancestral alleles are indicated with a and b. (…) We shall first answer the preliminary question: ‘what is the expected time since the other allele had a parent on the left side?’ and then multiply the answer by 2 to address genetic identity.’ | Undefined | A3ii |
| Campiglio, C. (2004) [74] | 3. Italy | Law (1) | ‘It has also been suggested that the duty to respect the identity of every child, according to the Convention on the Rights of the Child of 1989 (Art. 8) would include genetic identity too, and would therefore exclude any intervention whatsoever that intended to modify it ab origine. The right of the child to his genetic identity has been inferred also from principles 3 and 4 of the Declaration of the Rights of the Child of 1959, which recognize, respectively, the right to a name and a nationality, and the right to grow and develop in health. On the contrary, somatic gene therapy that is intended to ‘repair’ genetic disorders by causing permanent but non-hereditary alterations or to perform genetic engineering in order to cure some diseases (for instance, tumours) would be lawful. Different from germinal gene therapy, somatic gene therapy modifies the genetic inheritance of a single individual without being transmitted to descendants. | Undefined | C3 |
| Canellopoulou-Bottis, M. (2002) [75] | 3. Greece | Law (1) | ‘Constitution, Art. 5 being the major provision on the protection of the free development of personality and human freedom’. This (new) section reads: ‘everyone has the right to the protection of her health and her genetic identity. A statute shall provide the necessary protection of a person against biomedical interventions.’ The interpretation of the phrase ‘protection of genetic identity’ necessarily involves the protection of genetic personal data.’ | Undefined | D1 |
| Caplan, A.L. (2003) [76] | 1. USA | Bioethics (3) | ‘So, on the weighty matter of whether clones are people too, we could, based on our knowledge of twins … say, ‘Yes, clones are people too.’ Twins have a genetic identity identical to someone else, but they are no less people because of their genetic commonality.’ ‘The reason that scientists are interested in cloning for stem cells, as opposed to adult cells, bone marrow, or making stem cells from sperm and egg or frozen embryos is genetic identity. This would solve the problem of rejection.’ | Undefined | A2i  B7  C3 |
| Capps, W.H. (1993) [77] | 1. USA | Religious studies (3) | ‘Now all aspects of the debate must be re-examined in light of the Human Genome Project and its attempt to identify the ‘code of codes’ by means of which genetic identity can be discerned. (…) The startling likelihood is that if the genetic structure is discernible it must also be alterable, and if alterable it can be corrected and directed toward previously inconceivable human achievement.’ ‘To what extent should genetic predispositions be a factor in choosing a mate or having children? Should two individuals who know that their combined genetic identities will adversely affect their offspring have unlimited freedom to make reproductive choices, or should they be encouraged (and by whom?) to consider alternatives?’ | Undefined | C3  B2 |
| Carbone, J. & Gottheim, P. (2005) [78] | 6. Cuba | Law (1) | ‘These attitudes have changed as greater emphasis has been placed on genetic identity and the stigma associated with non-traditional families has lessened. Mary Shanley observes that: ‘The right to learn the identity of one’s genetic forebear stems from some people’s desire to be able to connect themselves to human history concretely as embodied beings, not only abstractly as rational beings or as members of large social (national, ethnic, religious) groups.’ | Undefined | A1 |
| Carmel, J.B. (2016) [79] | 1. USA | Medicine (4) | ‘Section 2 begins with a chapter by Drs. HongGeun Park and Jason Carmel [5], which discusses how precision of circuit manipulation can be achieved by genetic tools, based on the circuit location and/or its genetic identity.’ | Undefined | F1 |
| Carpenter, B.C. (2012) [80] | 1. USA | Law (1) | ‘If the survivor wishes to have a (or another) child, she must decide whether to commit to a new partner, use a sperm donor, or use the sperm or embryos she had preserved with her deceased partner. Some choose the latter because they are not ready to commit to a new partner (and may be nearing the end of their reproductive life), some do it to honor their deceased partner, and some do it to know the genetic identity of their child.’ | Undefined | A1 |
| Casey, R.P. (1992) [81] | 1. USA | Politics (2) | ‘From the beginning, each human embryo has its own unique genetic identity. Three-and-a-half weeks after conception, its heart starts beating. At six weeks, brain activity can be detected.’ | Undefined | C2 |
| Caterina, J.R. (2010) [82] | 1. USA | Law (1) | ‘Many people assume that adoptees who seek information about their genetic identities are maladjusted, anti-adoption, or ungrateful.’ ‘ The visits to the doctor get more frustrating each year, and the burden of not knowing my genetic identity carries with it a continuous anxiety over the fact that I have no idea what remains.’ | Undefined | A1 |
| Chatzinikolaou, N. (2010) [83] | 3. Greece | Religion (3) | ‘We open the book of the code of life and we find out that we cannot read it. We learn more about the genome in the hope that we are discovering the truth of our genetic identity, and we are led to the proteome, which confirms our greater ignorance. Before we can even get to grips with one alphabet, we are forced to learn an even more difficult language.’ | Undefined | E6  B6 |
| Chen, I.C. et al. (2016) [84] | 1. USA | Biology (4) | ‘… we studied a differentiated cell population and pluripotent iPSC lines derived from those cells. This approach facilitated direct comparisons of mutation frequencies in matched pluripotent/differentiated cell populations that shared a common origin and genetic identity and therefore differed only with respect to having attained a pluripotent or differentiated cellular state, respectively. This provided a tightly controlled assessment of the correlation between pluripotency and enhanced maintenance of genetic integrity.’ | Undefined | F1 |
| Cho, Y.K. (2015) [85] | 1. USA | Biomolecular engineering (4) | ‘However, it is also becoming clear that genetically defined neuronal identity does not always capture information provided by context. (…) Therefore, the genetic identity of a neuron needs to be mapped onto its anatomical structure (as provided to some extent in the above studies), as well as linked to its activity profile through electrophysiological and neurochemical characterization.’ | Undefined | F1 |
| Choi, J. (2011) [86] | 1. USA | Law (1) | ‘There are a myriad of social and policy concerns arising out of familial DNA testing. First, there is the worry that these searches erroneously embody the concept that criminality is biological. (…) points out that ‘*inferring the possibility of wrongdoing through genetic identity will stigmatize some groups than others …[which] creates the possibility of entrenching stereotypes that correlate race and ethnicity with criminality*.’ While this may carry some weight, it is still based on outsiders’ ignorance rather than the actual intent of such searches.’ | Undefined | D1 |
| Chorpening, J. (2004) [87] | 1. USA | Law (1) | ‘And, if the real risks lie with lower-skilled workers, then, until genetic testing is as simple as getting one’s genome microarrayed on a chip, employers are less likely to make an investment in investigating a lower-skilled worker’s genetic identity-except for specific occupational diseases such as..’ | Undefined | E6  D1  B2 |
| Christgen, M. et al. (2008) [88] | 2. Germany | Medicine (4) | ‘Infiltrating lobular breast cancer (ILBC) is a clinically and biologically distinct tumour entity defined by a characteristic linear cord invasion pattern and inactivation of the CDH1 tumour suppressor gene encoding for E-cadherin. (…) We report the establishment of a permanent ILBC cell line, named IPH-926, which was derived from a patient with metastatic ILBC. The DNA fingerprint of IPH-926 verified genetic identity with the patient and had no match among the human cell line collections of several international biological resource banks. ‘ | Undefined | F1 |
| Chua, H. (2017) [89] | 2. UK | Law (1) | ‘Notably, the common feature of the definitions for permitted gametes and embryos, even in their extended form, is that deliberate changes to their nuclear DNA are explicitly disallowed. This distinction is not as tenuous as it seems, because mitochondrial DNA contain 37 genes and are passed through the maternal line, whilst nuclear DNA contains 20,000 – 25,000 genes which are inherited from both parents. Hence, the latter more strongly embodies an individual’s genetic identity and editing nuclear DNA can be considered ‘full-blown’ germline editing. Henceforth the terms ‘germline editing’ and ‘germline therapy’ in this paper shall pertain to nuclear DNA. | Undefined | C3  E2  E3  E5 |
| Chumakov, P.M. (2007) [90] | 3. Russia | Molecular Biology (4) | ‘In spite of the vast diversity of different activities of the p53 molecule, each of them contributes to a common macrofunction, which is maintenance of genetic identity of somatic cells in a multicellular organism. The biological role of p53 is related to its important ‘social’ function in the organism, which ensures benefits of the organism over those of an individual cell. The function of p53 specifies altruistic behavior of cells in a multicellular organism, which is manifested by separate decisions of individual cells to commit suicide.’ | Undefined | F1 |
| Cicero, C. (2016) [91] | 3. Italy | Law (1) | ‘For a child, the need to know one’s own genetic identity is a need rooted in the depths of the human condition – a natural right which is vested in the person solely by virtue of his human dignity.’ | Undefined | A1 |
| Ciciarello, M. et al. (2007) [92] | 3. Italy | Molecular Biology (4) | ‘Mitosis is the most potentially dangerous event in the life of a cell, during which the cell genetic identity is transmitted to daughters; errors at this stage may yield aneuploid cells that can initiate a genetically unstable clone. The small GTPase Ran is the central element of a conserved signaling network that has a prominent role in mitotic regulation.’ | Undefined | F1 |
| Clark, B. (2011) [93] | 2. UK | Law (1) | ‘Article 8 does not define the concept of identity. Instead, it gives three examples of what identity includes: nationality, name, and family relations. Knowledge of one’s family relations is usually interpreted as going beyond knowing one’s legal parents and extending to biological and birth parents. (…) The United Nations Committee on the Rights of the Child appears to interpret the CRC as bestowing a clear right to donor-conceived children to knowledge of their genetic identity.’ | Undefined | A1 |
| Clayton, E.W. (2009) [94] | 1. USA | Law (1) | ‘In a nightmare of genetic discrimination, Jerome suffers numerous indignities as a result of his genetic heritage, from being denied admission to a nursery school for fear of liability were he to fall to being employed as a janitor. But this is a story of redemption, and by using the genetic identity of Eugene (literally, good gene), Jerome is able to fulfill his lifelong dream of becoming an astronaut.’ | Undefined | D1 |
| Cleal, B. & Gamble, N. (2015) [95] | 2. UK | Law (1) | ‘Crucially, the nuclear DNA from the mother (which carries all of her significant genetic identity) is retained, but the cell (and therefore all the other cells that replicate from it as a baby grows) are healthy. This enables a woman with faulty mitochondrial DNA to conceive a (healthy) genetic child, while removing the risk that mitochondrial disease will be passed to her children and future generations.’ | Undefined | E2  E3  A1 A2ii |
| Cobbe, N. (2007) [96] | 2. UK | Cellular biology (4) | Moreover, since even a sloughed human skin cell is recognized as human in terms of both its specific origin and corresponding genetic identity rather than its telos (which is non-organismal), what status should the mere presence of a human nucleus confer in the absence of any intrinsic potential for further significant and organized development?’ | Undefined | F1  E2  B7 |
| Cockfield, A.J. (2011) [97] | 1. Canada | Law (1) | ‘Other highly sensitive forms of personal information, such as genetic identity via DNA samples, increasingly are collected and stored in databases by state agents, creating potential problems of mistakes, tampering, outside access and so on.’ | Undefined | D1 |
| Cockfield, A.J. (2004) [98] | 1. Canada | Law (1) | ‘Finally, governments are increasingly gathering information on our genetic identity (…) the police canvassed certain neighborhoods and requested swabs from the inside of cheeks of hundreds of residents to conduct DNA analysis. The DNA was then checked against a databank of DNA samples to determine whether an individual could be a suspect.’ | Undefined | D1 |
| Cockfield, A.J. (2003) [99] | 1. Canada | Law (1) | ‘Finally, it should be noted that governments are increasingly gathering information on our genetic identity (...) the police canvassed surrounding neighborhoods and requested swabs from the cheeks of hundreds of residents to conduct DNA analysis. Each individual’s DNA was then checked against a databank to determine whether the individual could be a suspect.’ | Undefined | D1 |
| Cohen, D.M. (2001) [100] | 1. USA | Law (1) | ‘In sexual reproduction, a new form of life constitutes a convergence of the genetic identity of each parent. It is for this reason that a child carries physical traits of both parents, not only in the genotypes such as hair, eye, and skin color, but also in recessive traits that may later appear in the child’s offspring.’ | Undefined | A2ii  A1 |
| Cohen, I.G. (2011) [101] | 1. USA | Law (1) | ‘Genetic identity does not ensure narrative identity –identical twins share the same genes but are different people. Thus, it is also not a claim about identity and lack thereof in all senses of the word. It is the weak claim that if we want to know whether the person that results from the particular sperm and egg combination would be harmed, we cannot say that it would further the welfare of that person if we instead substituted a different sperm and egg combination. Philosophers often refer to this as ‘numerical identity,’ two entities are not the same because there are two of them.’ | Undefined | A2i  B1  C3 |
| Cohen, I.G. (2012) [102] | 1. USA | Law (1) | ‘Genetic identity does not ensure narrative identity –identical twins share the same genes but are different people. Thus, it is also not a claim about identity and lack thereof in all senses of the word. It is the weak claim that if we want to know whether the person that results from the particular sperm and egg combination would be harmed, we cannot say that it would further the welfare of that person if we instead substituted a different sperm and egg combination. Philosophers often refer to this as ‘numerical identity’; two entities are not the same because there are two of them.’ | Undefined | A2i  B1  C3 |
| Cohen, I.G. & Coan, G.T. (2013) [103] | 1. USA | Law (1) | ‘(…) these arguments have largely focused on whether children have a right to their genetic identity, whether they are harmed by a lack of connection to their genetic parents, whether intended parents (i.e., those who intend to rear the child and find a sperm or egg donor or surrogate to assist them) have a privacy or other interest in not sharing this information, and concerns about the commodification and corruption of reproductive practices.’’ | Undefined | A1  D1 |
| Cohen, J. (2003) [104] | 1. USA | Law (1) | ‘The genetic identity of the child was a key factor that distinguished Culliton from a previous case, R.R. v. MH., where the Supreme Judicial Court declined to enforce a surrogacy agreement. In R.R., the gestational mother was also the genetic mother, having been artificially inseminated with sperm from the intended father. The woman, who had agreed to be a surrogate mother, changed her mind after she became pregnant. The court relied on the adoption statute and refused to enforce the terms of the surrogacy agreement to force her to give up her parental rights.’ | Undefined | A1 |
| Collins, E. (2013) [105] | 1. USA | Law (1) | ‘Thirdly, the use of genetic information as identification has created a new form of identity theft: genetic identity theft. (…) Genetic identity theft delves far deeper into an individual’s personal information than simply a social security number; genetic identity theft includes physical characteristics and genetic makeup, a far more egregious violation of privacy. Without legislative protection against DNA theft, people’s safety and security are left defenseless.’ | Undefined | D1 D3 |
| Conley, J.M. & Makowski, R. (2003) [106] | 1. USA | Law (1) | ‘The PNG- I cell line required the addition of two cell types before a long-term cell line became established. It was also dependent on chemical additives to sustain optimal growth. To assess changes in the transformed cell line, comparisons were made between cell-surface proteins from the PNG-l cells and from the two cell types that were used in coculturing. There were no cell-surface protein markers in common between PNG-l cells and the two additional cell types. This is evidence that the PNG-l cell line retained its genetic identity.’ | Undefined | F1 |
| Conley, J.M. & Makowski, R. (2003) [107] | 1. USA | Law (1) | ‘In that patent, it will be recalled, tests showed that the cells of interest retained their genetic identity through the co-culturing process. They differed from their natural occurring counterparts only in that they had been induced to grow in an artificial medium, a medium that could not be replicated in nature. The sole reason for producing this medium, however, was to perpetuate the cells of interest for research and therapy purposes, and such purposes could be achieved only if the cells retained their genetic identity.’ | Undefined | F1 |
| Conley, J.M. & Makowski, R. (2004) [108] | 1. USA | Law (1) | ‘In that patent, it will be recalled, tests showed that the cells of interest retained their genetic identity through the co-culturing process. (…) The sole reason for producing this medium, however, was to perpetuate the cells of interest for research and therapy purposes, and such purposes could be achieved only if the cells retained their genetic identity.’ | Undefined | F1 |
| Connolly, U.  (2008) [109] | 2. Ireland | Law (1) | ‘Kording in her article proposes a broader view of the position or role as father, one based not on genetic identity alone. Baron has also rejected the genetic model, basing her arguments on the works of Lacan and Freud who place emphasis on the role of the father figure rather than his genetic identity.’ | Undefined | A1  B1 |
| Constand, S. (2013) [110] | 4. Australia | Law (1) | ‘However, this genocentric approach essentially equates genetic identity with personal identity. Yet it may be contended that a human is more than the sum of his or her genealogical composition, an argument that renders the metaphysical conflation of the human genetic makeup and human essence somewhat tenuous. Under this view, genes are not inherently tied to the essence of an individual. (…) From a purely chemical perspective, human genes may therefore be considered merely another arrangement of complex molecular structures that is shared by several other living organisms, and as such they are not necessarily solely responsible for creating the unique nature of humans.’ | Undefined | B7  B5  B1 |
| Crespi, G.S. (2008) [111] | 1. USA | Law (1) | ‘The long-term consequences of radically present-oriented policies and the ethical questions they present are quite subtle and complicated by what I call the problem of person-altering consequences. The decision whether to undertake such a policy (…) should be made solely on the basis of an assessment of the consequences for existing persons, and not upon any claimed ethical obligations to future generations distant enough from us in time for their members to all have had their genetic identities significantly altered by those person-altering consequences. We have no ethical obligations to these distant future generations based on conventional ethical premises to consider their rights or interests in making environmental or other policy decisions, because virtually nothing that we could possibly do would harm any specific future persons, counter-intuitive as this claim may seem.’ | Undefined | C3 |
| Crespi, G.S. (2009) [112] | 1. USA | Law (1) | ‘The phrase ‘person-altering consequences’ refers to the idea that one of the consequences of any significant policy is that it will lead to exponentially cascading and eventually universal changes in the fundamental genetic identities of future persons. The implementation of that policy will therefore be a necessary condition of the existence of those future persons, and the policy would obviously be very highly valued by them as a result regardless of its other consequences for their welfare.’ | Undefined | C3 |
| Crespi, G.S. (2010) [113] | 1. USA | Law (1) | ‘Genetic identity is thus an endogenous policy variable after a lapse of probably no more than a few decades. Put another way, one dramatic consequence of any policy measure, even one of limited and localized initial impact, is that over the longer-term it will eliminate the coming into existence of many and eventually all future persons who would otherwise have been conceived and born. It will result instead in the conception and birth of an increasingly and eventually entirely genetically different group of people, with their genetic endowments also increasingly diverging over time from that of the persons who would otherwise have been born. The policy will thus fundamentally alter the personal identities of all members of distant future generations; one vast group of what might be referred to loosely as ‘potential persons’ will now never be conceived and born, and will be replaced by an entirely different group of individuals.’ | Undefined | C3 |
| Crespi, G.S. (2010) [114] | 1. USA | Law (1) | ‘In my previous work I have referred to the fact that the fundamental genetic identities of the members of future generations are impacted by the current social polices that we pursue-in other words, that their fundamental identities are not exogenous to those policies, but are endogenously determined by them-as the ‘problem of person-altering consequences.’ (…)Which particular one of the hundreds of millions of sperm that are released in an ejaculation will unite with the female egg, if any, is a very uncertain event. Even the slightest difference in the timing or any other aspects of a reproductively successful act of intercourse will almost certainly lead to a different sperm-egg fusion, and therefore ultimately to the birth of a genetically different individual than would otherwise have been born. The person now conceived and born will be a different individual in the most fundamental genetic sense.’ | Undefined | C3 |
| Cronin, A.J. & Douglas, J.F. (2013) [115] | 2. UK | Biomedical sciences (4) | ‘The most desirable donation, as was the case in the first successful transplant by Murray in 1954, is between young adult identical twins, whose genetic identity precludes any tissue incompatibility and whose kidneys are unaffected by deterioration related to age or hypertension…’ | Undefined | A2i |
| Culty, M. (2009) [116] | 1. Canada | Medicine (4) | ‘This review summarizes the findings regarding the genetic identity of gonocytes, providing a description of the ‘‘common’’ gene expression profiles of fetal and neonatal gonocytes, as well as information on the main regulatory factors of gonocyte functions. A better comprehension of gonocyte development should help in the understanding of how germline stem cells are formed, possibly providing valuable clues on the origins of germ cell tumors or infertility.’ | Undefined | F1 |
| Da Costa Francez, P.A. et al. (2012) [117] | 6. Brazil | Forensic genetics (4) | ‘Insertion–deletion (INDEL) markers are very frequent in the human genome and present several advantages for population and forensic studies (…) The great adaptability of INDELs for amplification of low copy number or degraded DNA allows its using as an interesting platform of genetic identity by DNA in forensic cases.’ | Undefined | D1 |
| Daar, J.F.  (1998) [118] | 1. USA | Law (1) | ‘As noted by myriad commentators, any person born as a result of somatic cell nuclear transfer will mature and gain influence from a historical timeframe, family structure, and parental interaction different from those of the adult from which his or her genetic identity was gleaned. Scientific journals are replete with studies showing the profound influence environmental factors play in psychological, social, and physical development, so as to dismiss any notion that a human clone would be, in every sense, exactly the same as its adult predecessor.’ | Undefined | B6  A1  B1  E2  C3 |
| Dal Cin, P. & Van den Berghe, H. (1997) [119] | 2. Begium | Pathology (4) | ‘We have gained new insights in how tumors may arise, and some soft tissue tumors besides their identification by pathology now also have a genetic identity. This genetic identity is defined by: specific chromosome changes and by molecular changes related to the chromosome anomalies.’ | Defined | F1 |
| Das, B. et al. (2001) [120] | 1. USA | Medicine (4) | ‘GENETIC IDENTITY AND DIFFERENTIAL EXPRESSION OF P38.5 (HAYMAKER) IN HUMAN MALIGNANT AND NONMALIGNANT CELLS’ | Undefined | F1 |
| Davies, J.R. et al. (1999) [121] | 2. Sweden | Molecular Biology (4) | ‘In this study we have, for the first time, investigated the genetic identities of the major mucins which are present in human CF airway secretions using antisera raised against regions within the non-glycosylated domains of the MUC5AC, MUC5B and MUG2 mucins.’ | Undefined | F1 |
| De Andrade, N.N. (2010) [122] | 3. Italy | Law (1) | ‘As a right to identity that encompasses genetic characteristics, the protection of genetic identity must be understood within the broader concept of the right to identity. Accordingly, ‘genetic information is a component of, and therefore does not equate to, identity’.’ ‘The right to genetic identity, therefore, should both foresee the integrity but also the changeability of one’s genetic architecture: the right to personal identity may perfectly encompass the right to individual genetic modification.’ | Undefined | C1  D1  B1  C3 |
| De Meeus, T. (2018) [123] | 2. France | Biology (4) | ‘Temporal Wahlund effect should have similar consequences as geographic Wahlund effect though it may affect total genetic identity more strongly, depending on the cohort composition of each subsample and drift speed (hence population structure).’ | Undefined | A3ii |
| DeBre, K.D. (1989) [124] | 1. USA | Law (1) | ‘An individual’s knowledge that he possesses a patented genotype may create a perception that he is ‘less human’ than non-patented individuals. If the trait is one that is commonly available in the marketplace, the individual may see himself as an article of manufacture and hence inferior to other members of society. If one’s basic nature, one’s genetic identity, becomes subject to external manipulation, one’s ability to conceive of oneself as a free and rational being may weaken and ultimately disappear.’ | Undefined | B7  D3  C1 |
| Deckers, J. (2007) [125] | 2. UK | Healthcare ethics (3) | ‘Eberl also claims that ‘unique genetic identity (which the zygote does posess)’ is a necessary condition for ensoulment, but this is also unconvincing. While Eberl is correct that all zygotes have unique genotypes, if monozygotic twins do not have unique genotypes, the view that they are ensouled after implantation is inconsistent with the claim that ‘unique genetic identity’ is a necessary condition for ensoulment. (…) The embryo’s individuality starts from the moment when two gametes enter into contact with each other (...) Thus, an embryo’s individuality starts before (s)he has gained his or her genetic identity as sperm and ovum start to interact before syngamy, thereby becoming a new entity.’ | Undefined | C2  C1  B3 |
| DeCoursey, T.E. et al. (2002) [126] | 1. USA | Molecular Biophysics and physiology (4) | ‘Neither gp91phox nor any of its homologues has been demonstrated to function as a proton channel. On the contrary, expression of gp91phox does not result in H+ currents. Furthermore, all known components of H+ current in resting and activated phagocytes persist in cells lacking gp91. Because the molecular and genetic identity of the voltage-gated proton channel remains unknown, we still need to identify this molecule.’ | Undefined | F1 |
| Deech, R.L. (1998) [127] | 1. UK | Law (1) | ‘DNA testing which can establish with certainty the father of the child was introduced by the Family Law Reform Act 1987,19 but is confined to blood samples. (…) The accuracy of DNA testing is based on the unique genetic identity of each child, composed of the genes of its two parents. It was at first regarded as shocking and almost suspect by judges who (…) seemed to feel that to *know* who one’s father was, was something never intended by nature.’ | Undefined | A1  D1 |
| Deech, R.L. (1999) [128] | 1. UK | Law (1) | ‘You might conclude that because natural identical twins are generally all right, it therefore follows that a unique genetic identity is not essential for a human being to feel and be individual and to have his or her own personality. (…) Although the twins are identical to each other, they are genetically mixed because they came from two parents. The point about the clone is that there is only one parent.’ | Undefined | C1  C3 |
| Den Dunnen, J.T. (2015) [129] | 2. Netherlands | Clinical genetics (4) | ‘The DNA Bank: High-Security Bank Accounts to Protect and Share Your Genetic Identity’ | Undefined | D1 |
| Deng, L. et al. (2015) [130] | 5. China | Computational Biology (4) | ‘In summary, this study revealed that the genetic identity of the Malays comprises a mixed entity of multiple ancestries represented by Austronesian, Proto-Malay, East Asian and South Asian, with most of the admixture events estimated to have occurred 175 to 1,500 years ago, which in turn suggests that geographical isolation and independent admixture have significantly shaped the genetic architectures and the diversity of the Malay populations.’ | Undefined | A3i |
| Dennison, M. (2007) [131] | 1. USA | Law (1) | ‘Shortly thereafter, in 1989, the United Nations Convention on the Rights of the  Child recognized the ‘right to know one’s parents’ as a fundamentally important human right. (…) As one delegate claimed, ‘[i]ncreased knowledge and a gradual shift in attitudes has enabled us to acknowledge that in our contemporary culture young people have strong moral claims to know their genetic identities. It is now time for these moral claims to be converted to legal rights.’’ | Undefined | A1 |
| Destro, R.A.  (1986) [132] | 1. USA | Law (1) | ‘In Roe, the Court espoused the view that the unborn offspring of human parents, a human fetus, must be capable of ‘meaningful life’ outside the womb before he or she can be can be considered as having anything more than the ‘potential’ for human life. Genetic identity is, under this formulation, irrelevant, except to determine ‘potential’ for human life. But this ‘potential’, standing alone, is, in the Court’s view, insufficient to permit legal recognition of the unborn offspring of human parents as a ‘person’ under the fourteenth amendment. More – ‘meaningful life’  is required.’ | Undefined | C2  B7 |
| Deutsch, J. (2005) [133] | 1. USA | Law (1) | ‘In defense of the genetic donor’s priority, Professor Hill noted a ‘genetic-identity’ claim, suggesting that ‘an important aspect of parenthood is the experience of creating another in one’s own likeness. Part of what makes parenthood meaningful is the parent’s ability to see the child grow and develop and see oneself in the process of this growth.’’ | Undefined | A1 |
| Dickens, B.M. (1979) [134] | 1. Canada | Law (1) | ‘The test of replacement by natural processes of repair may in a sense be met by the unfertilized ovum; in itself it is unique, but within finite limits an ordinarily healthy woman who is not pregnant will be able to replace it in the normal course of her monthly menstrual cycle. A fertilized ovum, bearing the genetic identity of a unique being, may not be replaceable in this sense, however, and may therefore be ‘tissue.’’ ‘The human origin and potential of the ovum are clear, and its capacity upon fertilization for growth into a human person of unique identity follows from its nature.’ | Undefined | C2 |
| Dickman, D.G. (1985) [135] | 1. USA | Law (1) | *‘[T]hough not yet organized into distinctive parts or organs, the [embryo] is an organic whole, self-developing, genetically unique and distinct from the egg and sperm whose union marked the beginning of its career as a distinct, unfolding being. While the egg and sperm are alive as cells, something new and alive in a different sense comes into being with fertilization. (…) For after fertilization is complete, there exists a new individual, with its unique genetic identity, fully potent for the self-initiated development into a mature human being, if circumstances are cooperative. Though there is some sense in which the lives of egg and sperm are continuous with the life the new organism-to-be (or, in human terms, that the parents live on in the child or child-to-be), in the decisive sense there is a discontinuity, a new beginning, with fertilization.’* | Undefined | C2 |
| Dieterlen, F. & Lucotte, G. (2005) [136] | 2. France | Molecular anthropology (2) | ‘This study demonstrates that the geographic distribution of Y-chromosome variation for p49a,f *TaqI* haplotype XV reveals an important genetic identity for populations that live in the Occidental part of Europe.’ | Undefined | E4  A3i |
| DiFonzo, J.H. (2005) [137] | 1. USA | Law (1) | ‘DNA does, indeed, work. Theoretically, forensic DNA analysis of a thirty-two year old semen sample should be just as accurate as examination of bodily fluid from a rape kit collected the day before. But DNA’s capacity to survive the ravages of time attests only to the durability of genetic identity.’ | Undefined | D2 |
| DiFonzo, J.H. & Stern R.C. (2007) [138] | 1. USA | Law (1) | ‘DNA, for example, appears to be everlasting and immutable. But the durability of genetic identity supplies no confirmation that any particular DNA introduced at trial has been appropriately collected, analyzed, and preserved, nor whether the testimony regarding the DNA ‘match’ will be truthful or accurate.’ | Undefined | D2 |
| Dimond, R. et al. (2014) [139] | 2. UK | Social Sciences (2) | ‘Biosociality recognises a central role for biomedical knowledge in constructing genetic identities and producing and reproducing social relationships. Accordingly, it is often imagined as a new form of social solidarity.’ | Undefined | B1 |
| Dolgin, J.L. (2008) [140] | 1. USA | Law (1) | ‘In ‘families of shared DNA,’ genetic links displace tradition and ultimately challenge choice. Yet, more often than not it seems, the self-conscious effort to create enduring familial ties to ‘kin’ connected only through DNA is unsuccessful For instance, searches for kin, carried out by children conceived from the sperm of anonymous donors, do encourage participants to focus on genetic identity. But that focus seems to facilitate a form of individualism dependent on a notion of self-as-DNA rather than to facilitate enduring family relationships.’ | Undefined | A2ii |
| Dove, E.S. (2013) [141] | 1. Canada | Law (1) | ‘Control of dissemination of personal information is curtailed, and the disclosure of unexpected or adverse findings about the genetic identity of families violates dignity, which lies at the core of all humans.’ | Undefined | D1 |
| Dowd, N.E. (2003) [142] | 1. USA | Law (1) | ‘Genetic ties should, on the other hand, create identity rights. The child has a right to know their genetic identity, most strongly for medical and health reasons, but also to value cultural and social identity.’ | Undefined | A1  B1 |
| Dowd, N.E. (2005) [143] | 1. USA | Law (1) | ‘Genetics may provide the opportunity for fatherhood, but it would not be the sole trigger. So this means taking the existing standard and reworking it, based on the acknowledgement that men and women are not differently situated with respect to genetic identity, because DNA analysis removes that difference. The remaining difference is that women have acted, prebirth, to nurture; men's nurture in that time frame must be differently measured. But postbirth, the period that I focus on here, that difference disappears and permits a gender neutral standard.’ | Undefined | A1 |
| Dowd, N.E. (2006) [144] | 1. USA | Law (1) | ‘Social fatherhood values relationships and benefits the child. My definition, therefore, places me firmly in the camp of those who have argued against severing relationships based on after-the-fact discovery of the lack of a genetic link between father and child. (…) have argued for a model of universal DNA testing at birth or an acknowledgment of paternity that would stop any later DNA evidence to the contrary. This would re-orient the notion of fatherhood away from genetics by making genetic identity known, but grounding fatherhood in commitment and care.’ | Undefined | A1  B1 |
| Dreyfuss, R.C. & Nelkin, D. (1992) [145] | 1. USA | Law (1) | ‘The interest in genetic identity includes a preoccupation with biological determinism. Among the traits attributed to genetics are mental illness, homosexuality, aggressive personality, dangerousness, job and educational success, exhibitionism, the tendency to commit arson, stress, risk-taking, shyness, social potency, traditionalism, and even zest for life. These complex conditions frequently are described as directly inherited, as if they were single-gene disorders.’ | Undefined | B5  A2ii |
| Du Toit, C.W. (2002) [146] | 7. South Africa | Theology (3) | ‘Genetic identity is closely linked to the more immediate environment in which  the cell and organism find themselves. The environmental niche of the organism determines what genotype, what biochemistry, is selected and maintained.’ | Undefined | B6 |
| Ducharme, Howard M. (2001) [147] | 1. USA | Philosophy (3) | ‘The nature of genes and neuronal activity are not necessarily restrictive in telling us what it is to be a person. Persons are nonreductive moral agents who exist in the world, whose basic activities may be mediated, modified, and influenced by genetic and neurological factors. But persons are not reducible to genetic activity and/or neurological brain functions. We have first-person knowledge of ourselves as persons (in contrast with inferential explanations of ourselves via abstract theories). This direct knowledge includes being self-conscious, having free will, enduring with a strict personal identity through time in spite of the ever-changing neuronal and genetic identity of our bodies, having an irreducible moral nature and inherent moral value, and being aesthetic and religious beings.’ | Undefined | B1 |
| Duddington, J. (1999) [148] | 2. UK | Law (1) | ‘We are concerned with cloning in the sense understood in the Report from the Human Genetics Advisory Commission (HGAC) and the Human Fertilisation and Embryology Authority (HFEA) [1998] where it is defined as ‘producing a cell or organism with the *same* [my italics] nuclear genome as another cell or organism. This definition therefore assumed that the entire genetic identity of an individual is copied from one cell but, as we have seen above, this is not strictly true, because some genetic material from outside the nucleus will have also been copied.’ | Undefined | E2  C3 |
| Dunne, L.M. (2010) [149] | 1. USA | Law (1) | ‘In Judge Sweet stated in his opinion denying the defendants’ motions to dismiss: ‘[t]he challenges to the patents-in-suit raise questions of difficult legal dimensions concerning constitutional protections over the information that serves as our genetic identities and the need to adopt policies that promote scientific innovation in biomedical research.’ This statement, characterizing genes as information rather than chemical molecule, suggests that he may be more aligned with the ACLU's argument that gene patents constitute a ‘monopoly on information.’ This misguided interpretation of the patents-in-suit could have potentially harmful implications on his ultimate ruling on the plaintiffs' cause of action under the First Amendment.’ | Undefined | D1 |
| Dunstan, G.R. (1983) [150] | 2. UK | Theology (3) | ‘It would be tedious to recite the points of agreement and nuances of difference between the three submissions on the ancillary matters which the Warnock Committee has to consider: AID or IVF for the unmarried; surrogate motherhood; the law of legitimacy and the registration of AID births; the AID child's knowledge of his genetic identity, etc.’ | Undefined | A1 |
| Dunstan, G.R. (1988) [151] | 2. UK | Theology (3) | ‘Under (3) the familial and social dimensions of screening raise questions of the disclosure of information and the keeping or extending of confidences. Last comes the value placed on truth in two related areas of developing practice. In infertility treatment, the donors of gametes are required to remain anonymous. Gene tracing through families requires for its effectiveness some correspondence between assumed identity and genetic identity.’  ‘The more the study moves out from the individual subject to the family, the more important is the assumption that social identity–who we believe we are–coincides with genetic identity–who genetically we are. Uncertainty of parentage arising from human waywardness is morally irrelevant. If this be so, is it not time to call into question the insistence on anonymity for donors of gametes in the medical remedying of infertility?’ | Defined | A1  B1 |
| Earl, J. et al. (2016) [152] | 3. Spain | Cell Biology (4) | ‘It has been reported that UM-UC-2 is a T24 contaminant (…). We have used fingerprinting analysis to confirm this fact and the genetic identity of the cells/DNAs used in our experiments (Table 1). | Undefined | F1 |
| Ehlers, V.J. (1998) [153] | 1. USA | Politics (2) | ‘All of us have had the privilege of receiving a unique combination of genes from our parents. We delight in the knowledge that we have our own genetic identity. However, a child cloned from an adult would be denied this experience. Instead, the child would live in the shadow and expectations of his predecessor.’ | Undefined | C1  C3 |
| Elhauge, E. (2013) [154] | 1. USA | Law (1) | ‘Likewise, if in some future world, parents could order up whatever genes they want their children to have, wouldn’t they have a duty to equip their children with the best genes they could? In such a world, I do not think it would be a defense for a parent to say, ‘Yes, I did a terrible job picking your genes, but you are better off than if I had not created you at all, so I did not really harm you, and you would not have the genetic identity you have now without the terrible genes I gave you.’ In other words, I think a world where pre-birth enhancements can manipulate the genes our children carry problematizes the baseline notions about the relation between identity and genetic makeup that drive Cohen’s non-identity argument.’ | Undefined | C3  B2 |
| Ellioth, C. & Brodwin, P. (2002) [155] | 1. USA | Bioethics (3) | ‘Tracing genetic identity can lead to resolution of uncertainty but can cause more problems than it solves. Will establishment of genetic identity be cohesive or divisive?’ | Undefined | A3i |
| Ephross, J.N. (1992) [156] | 1. USA | Law (1) | ‘Current jurisprudence does not support the concept of treating the frozen embryo as a person. However, as with abortion, the legality of an action may depend on whether an interest cognizant of protection comes into being at conception, implantation, viability, or birth.’ It may be that the concept of a person involves a ‘cluster of features’ relating to an individual’s biological and psychological makeup, reasoning capacity, and intrinsic nature, and an embryo ‘lies in the penumbra region where our concept of a person is not so simple.’ An embryo may have a unique genetic identity, but lack the ‘cluster of features’ associated with a person; or, lack sufficient potential for development to be cognizant of independent protection. | Undefined | C2 |
| Esmaili, S. (2007) [157] | 1. USA | Law (1) | ‘As the District Court for the Eastern District of New York properly recognized, ‘[a] saliva sample can provide a significant amount of genetic identity information and it is generally not an item in the public domain.’ The host of medical and biological information contained within a DNA sample represents information that individuals typically seek to keep from the public sphere (...) DNA, unlike a fingerprint, does not merely contain a record of an 157individual’s identity but vast amounts of personal information including physical health and behavioral characteristics, among others. According to the Human Genome Project, ‘DNA can provide insights into many intimate aspects of a person and their families including susceptibility to particular diseases, legitimacy of birth, and perhaps predispositions to certain behaviors and sexual orientation.’ The nature of the information DNA provides ‘increases the potential for genetic discrimination by government, insurers, employers, schools, banks, and others.’ | Undefined | D1 |
| Falliers, C.J. et al. (1971) [158] | 1. USA | Medicine (4) | ‘The main purpose of the present communication is to document the differences of monozygotic twins in relation to the clinical and laboratory manifestations of asthma. The procedures employed established with a high degree of probability the genetic identity of these pairs. It seemed impractical—and an added imposition on our volunteer subjects—to extend the study to include tissue transplantations, etc., in order to establish monozygosity with absolute certainty.’ | Undefined | A2i |
| Fallone, E.A. (2011) [159] | 1. USA | Law (1) | ‘The National Association of Evangelicals has issued the following policy statement explaining its opposition to embryonic stem cell research: *All humans, male and female, are made in the image of God (Genesis 1:27) and, therefore, have intrinsic dignity that should be respected and honored. Indeed, the breath of life in all human beings is a gift from God (Genesis 2:7) and thus inherently holy. The NAE has pledged to protect the sanctity of human life and to safeguard its nature. Thus, the NAE opposes all human cloning, including cloning human embryos for laboratory experimentation, as well as discrimination based on genetic identities.’* | Undefined | D1  C3 |
| Fergus, V.L. (1995) [160] | 1. USA | Law (1) | ‘Proponents argue that the hereditary link, the genetic identity, the cohesiveness of the family unit, and the resemblance of family members support the basis for giving parental status to the genetic parents.’ | Undefined | A1  A2ii |
| Ferragut, J.F. et al. (2015) [161] | 3. Spain | Health science and genetics (4) | ‘In the present work, we studied 109 unrelated Chueta individuals aiming to characterize their maternal gene pool composition. The entire mtDNA control region (1121 bp) was sequenced. The highest frequencies were found for haplogroups R0a and H, indicating a remarkable signature of Middle Eastern ancestry along with some degree of European admixture. These data confirm that Chuetas have been able to maintain some ancestral genetic identity.’ | Undefined | A3i  E3 |
| Ferre, F. (2000) [162] | 1. USA | Philosophy (3) | ‘This brings us to the second major area of usefulness offered by this  book: namely, its grinding into dust the popular cynicism one hears about the ‘selfishness’ of genes. Rolston goes after the purveyors of this value-laden invitation to psychogenetic egoism, people like Richard Dawkins and E.O. Wilson, and is merciless in his dissection of their views. (…) Intellectual honesty does not require that we see the living world just as a single-focused struggle to maximize genetic identity. If that were the case, sexuality, which rapidly dilutes every individual’s genetic information to insignificance across the generations, would be the ultimate paradox. Cloning, not mating, would express genetic selfishness far more effectively. Evolution should never have ventured beyond the asexual bacteria. But in fact sexually reproducing species vastly outnumber asexual replicators in plants as well as animals. Evolution must have other interests to serve besides genetically selfish ones.’ | Undefined | C3 |
| Feirreira, N. (2016) [163] | 2. UK | Law (1) | ‘At the end, as it is his aim, Khaitan is effective in offering some ‘principled criteria’ – if not clear-cut solutions – to determine claims made from dis- crimination law, such as that the law should also prohibit discrimination on grounds of weight, physical appearance and genetic identity (p. 50).’ | Undefined | D1 |
| Feuer, J. (2011) [164] | 5. Israel | Law (1) | ‘Paternal bloodlines, the genetic identity of one’s father is deemed very important in Judaism, Christianity, and Islam. Since the 1990s, to avoid problems of consanguinity, married Sunni Muslim couples are allowed assisted reproduction, but only with their own gametes. Egg donations are permitted from wives of the same husband, but sperm donation is prohibited. In contrast, Shiite Muslims are permitted to use donated gametes. The Catholic Church approves infertility treatments that do not interfere with the natural sexual relations between the husband and wife.’ | Undefined | A1  B3 |
| Forman, D.L. (1994) [165] | 1. USA | Law (1) | ‘Genetics also plays a key role in the formation of families. James Nelson suggests that rather than view families from polar perspectives, as ‘naturally’ genetic-driven entities on the one hand or purely ‘social artifacts’ on the other, we consider the possibility that genetic links offer a ‘narrative connection’ to the past that gives depth and meaning to our lives. Nelson’s theory may help explain the search by some adoptees for their genetic parents and the movement for open adoption. Empirical research on adopted children seems to confirm that the need for genetic identity and connection is an important one. | Undefined | B1  A1 |
| Fraga, M.G. et al. (2009) [166] | 6. Argentina | Genetics (4) | ‘To analyze the distribution of different haplotypes of mitochondrial control region DNA in a large sample of argentine population who assisted to the Banco Nacional de Datos Geneticos (BNDG) looking for their identity in cases of civil state suppression during the dictatorial government (1976–1983). From 1168 haplotypes analyzed, 794 were unique. Haplotype frequencies were estimated by haplotype counting. Values of Genetic Identity (P) and Genetic Diversity (h) were calculated, being 3.3939 x 10^-3^ and 0.9974 respectively.’ | Undefined | A2i |
| Franciosi, L.M. & Guarneri, A. (2008) [167] | 3. Italy | Law (1) | ‘Within this context, we find not only the analysis of the collection of different possible violations (intrusions), but also a list of entities capable of executing them, an array of legislative, doctrinal and jurisprudential sources that are used to protect the genetic identity of the individual, and an initial panel of solutions to the problems encountered so far. The reference to privacy rights in particular deserves to be highlighted because of the strong arguments supporting the idea that a violation of an individual’s genetic information could be deemed a violation of their right to privacy.’ | Undefined | D1 |
| Frankel, A. (2000) [168] | 1. USA | Law (1) | ‘Religious opponents also assert that human cloning is a violation of human dignity. Cloning violates human dignity because it deprives the clone from individuality and a unique identity. Although some cite the occurrence of identical twins as the counter-argument, opponents maintain that cloning is highly distinguishable because cloning is deliberate, while other forces dictate the occurrence of twins. In the case of identical twins, neither twin is the creator of the other, whereas cloning, by its volitional nature, subjects humans to the whims and manipulations of others. In the case of a clone, critics exclaim that they are ‘deliberately infused with a predetermined genetic identity... [and] saddled with a genotype that has already lived.’’ | Undefined | C1  C3 |
| Franklin, S. (2003) [169] | 2. UK | Anthropology | ‘The question remains as to whether ‘the medicalization of kinship’ and ‘the hegemony of the gene’ actually have the effect of reinforcing consanguineal ties, de-emphasizing conjugal and affinal ties, and deepening the significance of biological ties to distant kin, as Finkler claims (2000: 208), or whether this effect is somewhat undermined by her emphasis on the extent to which these ‘postmodern’ kinship ties are also profoundly alienating. ‘By assigning shared identities to people who may have little in common in the past or in the present’ and by linking them to ‘people with whom they have shared few experiences’, the ideology of genetic inheritance, in Finkler’s view, ‘distanc[es] the person from his or her being’, opening up a gap between their experience and their genetic identity (…). ‘ | Undefined | A2ii  B1 |
| Frazer, K.A. et al. (2007) [170] | 1. USA | Genetics, medicine (4) | ‘We show that 10–30% of pairs of individuals within a population share at least one region of extended genetic identity arising from recent ancestry.’ | Undefined | A3i  A2ii |
| Fredlake, C.P. et al. (2006) [171] | 1. USA | Biological engineering (4) | ‘Although a finished human genome reference sequence is now available, the ability to sequence large, complex genomes remains critically important for researchers in the biological sciences, and in particular, continued human genomic sequence determination will ultimately help to realize the promise of medical care tailored to an individual’s unique genetic identity.’ | Undefined | E6 |
| Frith, L. (2001) [172] | 2. UK | Medicine (4) | ‘… in systems where non-anonymous donation is practiced, an understanding of the proclaimed right of donor offspring to know their genetic identity is one composed of two different rights – the right to know the circumstances of their conception and the right to information identifying the gamete donor.’ | Undefined | A1  D1 |
| Gabel, J.D. (2010) [173] | 1. USA | Law (1) | ‘(…) a Fourth Amendment analysis seems appropriate where law enforcement requests the identity of a pivot after a partial or familial match has been found. As its name suggests, a familial search involves a search of the genetic identity and profile of a pivot, but is this any different from the search of a license plate or fingerprint database? Legally speaking, it is a tenuous argument to make when examined through an objective lens that views DNA database searches as nothing more than the combing through of available data. Even though courts routinely find that offenders have minimal interest in their genetic information after conviction, the situation here has nothing to do with the reasons surrounding the offender’s conviction. The disclosure of the pivot person’s identity to investigate a crime that he is innocent of should require a showing of probable cause (or something close to it) such that there is a likelihood that the pivot is related to the source.’ | Undefined | D1 |
| Gagnon, A. et al. (2005) [174] | 1. Canada | Sociology (2) | ‘The concept of genetic identity-by-descent (IBD) has markedly advanced our understanding of the genetic similarity among relatives and triggered a number of developments in epidemiological genetics.’ ‘When two genes at a given locus are inherited from a common ancestor, they are said to be identical-by-descent. While parent and offspring exactly share 50% of their genes on autosomal loci, two siblings share the same proportion on average. At any locus, they may share no alleles (if both parents transmitted different alleles), one allele (if one parent transmitted the same allele to each offspring), or 2 alleles (if each parent transmitted the same allele to each offspring).’ | Undefined | A2ii |
| Gamble, J. (2017) [175] | 1. USA | Philosophy (3) | ‘One such mechanism, the creation of new exogenous relay circuits bridging the injury, has been explored extensively, revealing serious impediments to its optimization and adoption for clinical settings. Our collaborator, the Sakiyama-Elbert group, has spent years addressing the first limitation, the variability of cellular graft composition, by perfecting protocols to generate embryonic stem cell (ESC)-derived populations of neurons with pre-determined genetic identity.’ | Undefined | F1 |
| Gandi, H. (2015) [176] | 1. USA | Law (1) | ‘While these tests can be used to pull previously unusable samples to potentially convict the culprit or exonerate the innocent, the technology itself presents potential problems for Fourth Amendment analysis. As these tests not only reflect the genetic identity of the person whose DNA is collected and analyzed, but also the genetic identity of certain members of their family, these tests can allow the government to circumvent consent or the need for a warrant.’ | Undefined | D1 |
| Gardner-Hopkins, J.D.K. (2001) [177] | 4. New Zealand | Law (1) | ‘Genetic discrimination is presently a minimal problem, but the risk of genetic discrimination may be exacerbated by the constant developments in genetic research. It is important for the law to provide clear guidance and ensure that science is not used to perpetuate discrimination and support social inequality. This requires lawyers, judges, policymakers, and politicians to have a sound understanding of genetics and ensure that all decisions are based on good science, which recognizes that undue emphasis and reliance must not be placed on a person’s genetic identity because genetic information is probabilistic and inexact.’ | Undefined | D1 |
| Gargiulo, P. & Nesi, G. (2000) [178] | 3. Italy | Law (1) | ‘*Those limits obviously entail the complete rejection of experiments that might lead to forms of cloning that cause modification in the so-called genetic identity of the human embryo. They also involve the rejection of every use of human embryos (...).’* | Undefined | C3 |
| Garstka, K. (2017) [179] | 2. UK | Law (1) | ‘Following physiological identity, art. 4(1) moves on to elevate one’s genetic identity in a similar fashion. Definition of genetic data from art. 4(13), as complemented by recital 34 of the Regulation, is that of ‘personal data relating to the inherited or acquired genetic characteristics of a natural person which result from the analysis of a biological sample from the natural person in question.’ Assuming that the Sensen tech would allow for the digitization of memories without the inclusion of any biological material from the brain, such memories would not automatically point towards the genetic identity criteria. As for the content of memories, in contrast to many instances previously discussed in this section, it would most likely be exceedingly difficult to find memories containing genetic data.’ | Undefined | D1 |
| Geisler, N.L. (1990) [180] | 1. USA | Theology & philosophy (3) | ‘According to Ford, ‘it is necessary to distinguish between the concept of  genetic and ontological individuality or identity’ (p.117). Genetic identity is established at fertilization. This is not, however, ‘speaking philosophically about the concept of a continuing ontological individual’ (ibid.). The ‘establishment of the new genetic programme at the completion of fertilization is a necessary, but not a sufficient, condition, for the actualization or coming into being of the new human individual at the embryonic stage of existence’ (p. 118). | Undefined | C2 |
| Gerards, J.H. & Janssen, H.L. (2006) [181] | 2. Netherlands | Law (1) | ‘Greece revised its Constitution by including protection for the ‘genetic identity’ and by strengthening the protection of personal data. The Swiss Constitution encompasses protection of human genetic material and data since 2002. The Greek and Swiss constitutional provisions provide protection which is oriented towards genetic privacy approach on a more abstract level. The Swiss Constitution explicitly mentions the right to informed consent. The Greek approach aims to include a concept of ‘genetic identity’. Protection, enforcement and exceptions have to be arranged by statutory law. Austria, Belgium, Germany, France, the Netherlands and the United Kingdom have no constitutional provisions explicitly referring to genetic information or to a ‘genetic identity’.’ | Undefined | D1 |
| Ghiggeri, G.M. et al. (2006) [182] | 3. Italy | Medicine (4) | ‘There is enormous potential for research on genetics of complex traits and on multifactorial mechanisms by studying identical twins, requiring efforts at describing single families and establishing twin registers (…) Also, the basic molecular approach was limited to human leukocyte antigens (HLA) polymorphisms that do not furnish an exhaustive picture of genetic identity, 25% of dizygotic twins being expected to share the same HLA haplotypes.’ | Undefined | A2i  F1 |
| Gianoli, E. & Valladares, F. (2011) [183] | 6. Chile | Biology (4) | ‘Phenotypic plasticity is traditionally defined as the capacity of a given genotype to render alternative phenotypes under different environmental conditions. Some studies focus on the individual genotype to study ‘true’ phenotypic plasticity, regardless of the level of ecological organization involved in each particular study. We argue that, depending on the research question and the scale, there are advantages of looking beyond the genetic identity of each individual phenotype when addressing phenotypic plasticity.’ | Undefined | B6 |
| Giddings, K.J. (1980) [184] | 1. USA | Law (1) | ‘This genetic identity crisis, or ‘genealogical bewilderment, primarily affects the individual’s development of self-concept. Successful identity development provides a positive self-image, enabling an individual to function effectively and confidently in society. Completion of this process occurs when the individual achieves emotional independence from the parents.’ | Undefined | B1  A1 |
| Godfrey, J.P. (1995) [185] | 2. UK | Human ecology (2) | ‘The Pope accepts the misconception that there is an instant when fertilization happens. The process of fertilization is complex, taking about two days. It can involve more than one sperm nucleus, at least for a time. During this period genetic identity is yet to be established. There is a rapid and irreversible change at the activation of the egg. Activation, though rapid, is not instantaneous. Its very speed is probably an evolutionary adaptation of eggs to inhibit fertilization by many sperms. (…) A more severe difficulty is that activation, essential though it is to further development, precedes the chromosomal events that establish the genetic identity of the zygote. So an new individual cannot have her or his origin at activation. For about the first four days, all the genetically determined properties of the fertilized egg are maternal. Only after this do paternal genes begin to act, with gene expression characterizing the new individual.’ | Undefined | C2 |
| Goodwin, J.A. (1997) [186] | 7. South Africa | Biochemistry and Microbiology (4) | ‘This requires a knowledge of the frequency with which the alleles represented occur within a population. This calculation is of particular importance, as DNA typing cannot claim to prove without doubt the genetic identity of two samples. Typing evidence, with the aid of population genetics statistics, can merely indicate that the two samples are unlikely to have originated from different persons.’ | Undefined | D1  A2i |
| Gorney, R. (1968) [187] | 1. USA | Medicine (4) | ‘We saw that it might be possible someday to recreate a genetically identical copy of a living man from a living cell, or a copy of one long dead from the specifications left in his mummified genes. And we remember that these resurrections, like all clones, could not be the same ‘person’ because neither could possess the experiences or knowledge of the original. Now, RNA is a much less stable and preservable substance than DNA. Furthermore, whereas theoretically one could reconstitute the individual’s *genetic* identity from the intact genes of only one cell, in order to reconstitute the *acquired* information in his RNA (and related substances), it would be theoretically necessary to have intact and in proper relation to each other all the twelve billion nerve cells of his brain (…).’  ‘But, as we have just seen, there is no possibility of narrowing the generation gap this way because there is no possibility of genetic identity between parents and sexually reproduced children. And what other kinds are there?’ | Undefined | E1  A2i |
| Goswami, S. (2012) [188] | 1. USA | Law (1) | ‘Aside from patenting the genetic identity associated with the disease, companies may also be interested in patenting the algorithm that predicts the propensity for metastasis of tumors or the interaction of multiple genes.’ | Undefined | D3  B2 |
| Graham, K.A. et al. (1986) [189] | 1. Canada | Medical biophysics (4) | ‘ To establish if these differences are due to a lack of genetic identity between current MCF-7 passages and the original cell line, we have performed karyological and restriction fragment length polymorphism (RFLP) analyses. (…) Using these criteria and karyotypic analysis it can be shown that a sample of MCF-7 obtained from the American Type Culture Collection (ATCC) was derived from a different individual than was the original MCF-7 cell line.’ | Undefined | F1 |
| Graham, K.T. (2008) [190] | 1. USA | Law (1) | ‘She becomes pregnant via artificial insemination and gives birth; her husband is regarded as the natural and legal father of the child. This is true despite the fact that the child’s genetic identity is unrelated to her husband.’ | Undefined | A1 |
| Greely, H.T. (2006) [191] | 1. USA | Law (1) | ‘Identity issues include, prominently, the forensic uses of DNA but also the possible use of DNA for establishing ‘ethnic identity’ and the controversy over human reproductive cloning, a form of assisted reproduction of particular interest only because of the almost complete genetic identity between the cloned and the clone.’ | Undefined | A2i  C3 |
| Greenfield, D.L. (2006) [192] | 1. USA | Law (1) | ‘The patenting of human genes proves problematic for indigenous peoples who do not subscribe to Western notions of property, but instead view genetic identity as intrinsic to all peoples.’ ‘Where unjust enrichment filled gaps in the tort laws cited in Greenberg, it is potentially available to remedy the harms created, but not addressed by intellectual property law, where the appropriation of one’s genetic identity violates cultural, spiritual, or religious mores and freedoms.’  ‘Just as Native Americans were dispossessed of their lands, ‘bioprospecting’ and patenting genes from isolated, indigenous populations strips those populations of their dignity and their genetic identity. As such ‘[s]ome indigenous groups have become suspicious of cell line prospecting and consider it a form of Western thievery of third world resources.’’ | Undefined | D3 |
| Greenlee, S. (2000) [193] | 1. USA | Law (1) | ‘The resolution relies on fundamental human rights as the authority for condemning the practice of human cloning: ‘[human cloning]… is unethical, morally repugnant, contrary to respect for the person and a grave violation of fundamental human rights which cannot under any circumstance be justified or accepted.’ The specific fundamental human right that the Resolution relies on is a person’s right to his own genetic identity.’  ‘We have heard of the right to determine one’s own genetic identity, the right to personal autonomy, and the right to choose. ‘ | Undefined | C1  C3 |
| Griffiths, R.C. (1980) [194] | 1. USA | Population genetics (4) | ‘Inequalities and approximations are found for Nei’s genetic identity at one locus when mutation rates vary, and also for the identity across loci when the overall mutation rates per locus vary. Genetic identity at the molecular level is considered and a probability generating function found for the number of segregating sites between two randomly chosen gametes from two divergent populations, under various models.’ | Undefined | A3ii |
| Grimm, D.J. (2007) [195] | 1. USA | Law (1) | ‘Instead, as a symptom of biology, relatives and future generations risk being stigmatized for their mere genetic similarity to a previously convicted defendant. Assuming possible criminality based on genetic identity replaces the wrongdoing requirement with what might be described as wrongbeing. Using biological identity as a proxy for wrongdoing is at odds with the Fourth Amendment (…).’ ‘Inferring the possibility of wrongdoing through genetic identity will stigmatize some groups more than others. (...) it follows that African Americans and Hispanics will face the sting of stigmatization far more often than others. Such a result creates the possibility of entrenching stereotypes that correlate race and ethnicity with criminality.’  ‘A more insidious scenario involves efforts to uncover race or ethnicity-based genetic differences among criminals, which could ‘permit the criminal law not only to be reactive, but predictive, by identifying would-be offenders on the basis of their genetic make-up. If realized, the correlation between genetic identity and race or ethnicity carries startling consequences.’ | Undefined | D1  A3i |
| Grosman, P.G. & Herrera M. (2005) [196] | 6. Argentina | Law (1) | ‘As we can see, the judgment considered that the conduct of the defendants, leading to a false attribution of paternity, harmed the husband’s right to identity, including his right to truth in relation to his position in family relationships (…) the court recognized that the right to identity is not confined to children, but belongs also to adults. It is a right ranking at the level of the Constitution (…) Genetic identity affects all who are linked through a particular bond of parentage.’ | Undefined | A1 |
| Gross, A. (2010) [197] | 5. Israel | Law (1) | ‘When, then, does fraud arise with regard to the actor’s identity? In its sentencing judgment, the court set a requirement for Alkobi and others like him to declare at a certain point their ‘biological-genetic identity’ and to reveal the fact that they are ‘male’ or ‘female’ in the narrow sense currently accepted in society. This ruling rests on the premise that somehow, despite everything stated, genitalia are the determinative factor, at least in the framework of social-intimate relations, and anyone who lives in a gender identity that deviates from the one designated by his or her genitalia must state this at some stage in the development of a romantic-intimate-erotic relationship.’ | Undefined | B4 |
| Gross, J.A. (2008) [198] | 1. USA | Law (1) | ‘Solid organ transplants first became a viable clinical option in the 1950s, but generally only between identical twins. Allocating organs according to genetic identity left little room for value judgments. From the start, transplant surgeons pushed the bounds of this narrow conception of the acceptable match.’ | Undefined | A2i |
| Gross, M. (2012) [199] | 2. Germany | Sociology (2) | ‘The heated debates (…) around the legal right not to (have to) know one’s own genetic identity are certainly a part of this. (…) Knowing that you do not know when (or whether) you may fall seriously ill once a probability has been established, is almost certain to make you feel afraid. The right not to know becomes relevant when, in the process of genetic testing, relatives are also informed (without their consent) about their genetic risk. (…) The right to nonknowledge is therefore intended to ensure that no one can be forced to acquire knowledge of their genetic characteristics.’ | Undefined | B2 |
| Gurnham, D. (2005) [200] | 2. UK | Law (1) | ‘But why should this be so important? I would agree that much of the concern arises from the idea that it disrespects a person to determine his genetic identity in advance. However, also at stake here is the fear that there will be an altering of normal family relationships. (…) On the possibility that a woman could use her own somatic cell nuclei and fuse them with one of her own enucleated eggs, the Catholic Truth Society states: ‘the choices made by the single woman will deliberately deprive the clone child of both a genetic and a social father, thereby distorting that child’s relations with the male sex’ (McCarthy, 2003: 48).’ | Undefined | C1  C3  B1 |
| Gurnham, D. & Miola, J. (2012) [201] | 2. UK | Law (1) | ‘In Rose v Secretary of State for Health two donor-conceived offspring each sought information about their biological fathers. Scott-Baker J in the Queen’s Bench Division found that Article 8 of the ECHR, which guarantees respect for privacy, was engaged by a person’s desire to gain information about their own genetic identity. He cited the European Court of Human Rights authority of Mikulic v Croatia, in which it was held that ‘respect for private life requires that everyone should be able to establish details of their identity as individual human beings and that an individual’s entitlement to such information is of importance because of its formative implications for his or her personality’. Scott-Baker J regarded that since the information related to ‘the very heart of [a person’s] identity, and to their make-up as people’ there arose an ‘interest [in seeking] information about him’. (…) In support of the former, John Eekelaar has argued that knowing the ‘physical truth’ about one’s own genetic heritage is necessary for children to ‘confront the world as it is *on his own terms*, and that to obscure it with ‘legal truths’ means that ‘society’s new members are doomed to manipulation’.’ | Undefined | A1 |
| Haag, L.J. (1994) [202] | 1. USA | Theology (3) | ‘I have argued that genetic identity cannot be advanced as the sole criterion of individuated personhood. Is there any event in the development of the conceptus which can be said to be determinative of the existence of individuated personhood? Viability outside of the uterine environment, currently possible at the twenty-fourth week of gestation, would hardly seem a significant milestone since physiological dependence or independence would seem to be irrelevant to personhood. One would not suggest, for example, that confinement to an oxygen tent vitiates personhood.’ | Undefined | C2 |
| Hackenberg, R. et al. (1994) [203] | 2. Germany | Medicine (4) | ‘DNA analyses confirmed the genetic identity between the MFE-296 cells and the patient, from whom the cell line was derived.’ | Undefined | F1 |
| Hagmann, W. et al. (1996) [204] | 1. USA | Medicine (4) | ‘Our RT-PCR data in combination with cDNA sequencing of amplified fragments provide novel and distinct evidence for the presence of the platelet-type 12-LOX isoform in A431 cells and define it as closely related or identical with the platelet enzyme. This characterization of 12-LOX is further supported by its antigenic reactivity, since the antiserum used in Western blots preferentially recognizes platelet-type 12-LOX. This is the first report of the genetic identity of a 12-LOX isoform from human solid tumor cells. Further, we found no evidence for the concomitant expression of leukocyte-type 12-LOX in A431 cells.’ | Undefined | F1 |
| Halliday, S. (2004) [205] | 2. UK | Law (1) | ‘In its abortion decisions the Bundesverfassungsgericht skillfully avoided the question of when life begins, however it did mphasize that at least from the time of implantation there could be an individual, no longer divisible, life with its own genetic identity and that from this point the entity could be protected by both Article1 I GG and Article 2 II GG.98 Whilst the court expressly stated that it was only concerned with the question of abortion, it is likely that this conception of a life capable of protection beginning at implantation/the formation of the primitive streak would also apply to situations other than abortion, including embryo research.’ | Undefined | C2 |
| Hallowell, N. et al. (2006) [206] | 2. UK | Public Health sciences (4) | ‘This study investigated high risk men’s responses to BRCA1/2 predictive genetic testing. Seventeen in-depth interviews were undertaken with carrier ( n = 5) and noncarrier men ( n = 12). All men described genetic testing as a familial duty. It is observed that carriers and non-carriers mphasize differing explanations about their role in the aetiology of risk. It is noted that men engage in a form of narrative reconstruction in which they draw upon discourses of guilt and blame or fate and predestiny in an effort to present themselves as morally responsible or blameless. It is argued that narrative reconstruction enables these men to reconcile their genetic identity, self and family.’ | Undefined | B2 |
| Hamblin, T. (1997) [207] | 2. UK | Medicine (4) | ‘Although thrombopoietin (TPO) has now been cloned, many pretenders have auditioned for the role. It is clear that interleukin-3, interleukin-6, interleukin-l l, stem cell factor, leukaemia inhibitory factor and oncostatin-M all influence the proliferation and maturation of megakaryocytes. This review details the nature of these non-TPO cytokines, giving their genetic identity, molecular structure, receptor, mode of action and range of activities. Their effect on platelet production in preclinical and clinical studies is reported and their potential as agents for thrombopoietic support is discussed.’ | Undefined | F1 |
| Hansen, C. (2006) [208] | 1. USA | Journalism (2) | ‘State Rep. Roger Hunt, the bill’s chief advocate, knows the Supreme Court math doesn’t look good now. But he believes the Court will be swayed by scientific evidence not available in 1973. Hunt argues that research shows unborn babies have a different genetic identity from their mothers and life begins at conception. Thus, he says, children should be protected separately under the law. He’s not alone in his hope that the Court will reconsider Roe in the next few years.’ | Undefined | C2 |
| Hari Kumar, K.V.S & Modi, K.D. (2014) [209] | 5. India | Medicine (4) | ‘Dizygotic twins are similar to two siblings and have different genetic information. In contrary, monozygotic twins have a similar genetic identity and provide a unique opportunity to evaluate the contribution of genetic and environmental factors of the disease’. | Undefined | A2ii  B6 |
| Harris, J. (1999) [210] | 2. UK | Bioethics (3) | ‘Rights to genetic privacy and genetic identity are often asserted by amateurs of popular science who lack awareness of the incoherence and mutual incompatibility of these alleged rights. Genetic privacy has to do with the privacy or secrecy of genetic information. Genetic identity is often confused with genetic uniqueness (the right to an exclusive genome). Genetic uniqueness cannot be a fundamental or basic human right because of the frequency with which it is violated without any harmful effects by the existence of monozygotic twins. Genetic identity, as opposed to genetic uniqueness, has to do with the extent to which our genes are shared with others. To put it crudely perhaps, the Hapsburg nose has to do with genetic identity, but is essentially public. It is therefore unclear how information about such genetic features could be the subject of privacy rights any more than could the identity of one’s progenitors.’ | Defined | A2ii  C1  D1 |
| Harrison, V. (1993) [211] | 1. USA | Religion (3) | ‘According to most physiological ideas current in patristic times, the father’s seed is the sole source of a child’s life while the mother only provides a receptacle in which it can grow. The Theotokos turns this upside down. Since she is the sole source of Christ’s human life, she shows that every mother is also a true source of life for her child. Of course, this is well known to modern science, which recognizes egg and sperm equally as life-principles, from whose union the child’s genetic identity is constituted.’ | Undefined | A1  C2 |
| Harris-Short, S. (2004) [212] | 2. UK | Law (1) | ‘As Van Bueren argues, ‘Article 8(1) specifically includes family relations as a fundamental element of identity and as such genetic and biological identity cannot be excluded. (…)This concern with the child’s right to know the truth about his/her genetic heritage clearly accords more closely with the interpretation of Article 8 advanced by Professor Van Bueren and, most importantly, alongside the express recognition afforded to the importance of family relations in Article 8, would, by necessary implication, seem to provide the desired protection under international law for the basic integrity of a child’s genetic identity. ‘  ‘However, whether the fundamental right at stake is articulated as a right to a unique and random genetic identity or, more generally, as a right not to be subjected to reproductive cloning, is really immaterial. The important point is that both rights, secured through a total prohibition on reproductive cloning, are derived from a common concern with the basic human dignity of the child.’ | Undefined | A1  C1  B1  C3 |
| Hartman, R.G. (2008) [213] | 1. USA | Law (1) | ‘The state courts ceded to parents’ decisions about compelling seven-year-old Margaret Hart and six-year-old Sydney Cowan to have their bodies surgically invaded and tissue extracted to benefit their sisters. Insofar as the best odds of success in transplantation are concerned, identical twins share a complete genetic identity.’ | Undefined | A2i |
| Hartshorne, C. (1972) [214] | 1. USA | Philosophy (3) | ‘Hence those who suppose that genetic identity is strict identity are contradicting the meaning of ‘genetic.’ It cannot be identity which explicates ‘time’s arrow,’ but only some way of being identical and nonidentical; or, to remove the contradiction, of being only partially identical.’  ‘Genetic identity, which has only a relatively definite meaning, involves (1) some ‘defining characteristic’ reappearing in each member of a sequence or family of occasions; (2) direct inheritance by appreciably positive prehensions of this character from previous members.’ | Defined | A2ii |
| Hawkins, A. (2001) [215] | 1. USA | Law (1) | ‘Another concern regarding the use of cloning for reproductive purposes is the alteration of the normal genetic relations in families. The cloned individual would contain the complete genetic identity of one parent and virtually no genetic connection to the other parent.’ ‘The purpose of this type of cloning is not to have a child with a genetic link to a parent, but rather to produce an individual with a specific genetic identity. Through the use of nuclear transfer, parents could make a child-to-order by selecting those traits that they desire for cosmetic, aesthetic, intellectual, and physical purposes.’ | Undefined | C3  B1  A1 |
| Heagle, K.A. (1998) [216] | 1. USA | Law (1) | ‘The European Parliament suggested that all funding for cloning research should be stopped immediately, and that penal sanctions should result from any violation of the ban. Parliament further stressed in its resolution that every individual was entitled to his or her own genetic identity without interference from cloning.’ | Undefined | C1  C3 |
| Hefner, P. (2002) [217] | 1. USA | Theology (3) | ‘Gattaca is an especially vivid example: Two brothers are in competition—one perfectly engineered, the other, a defective love child conceived in a moment of his parents’ backseat passion. The defective brother successfully hides his genetic identity and finally surpasses his brother when he saves him from drowning and is later selected to be an astronaut. It is as if these movies are repudiating denial and finding deeper value in finitude and mortality. There is at least one world religion that also suggests that a man who was defeated and executed on a cross turned out, finally, to be the victor. Or perhaps these movies are a sturdy witness to the belief that mortal, finite creatures have intrinsic worth of their own. In my Lutheran tradition, we call this Justification by Grace or, as it is known more technically, ‘It’s okay to be mortal.’’ | Undefined | D1 |
| Henaghan, M. & Ballantyne, R. (2013) [218] | 4. New Zealand | Law (1) | ‘It is also essential that a child’s right to know his or her identity, particularly his or her genetic identity, is preserved. This becomes even more important in the future where genetic medicine may become the dominant diagnostic tool in many healthcare systems.’ | Undefined | A1 |
| Hendricks, J.S. (2011) [219] | 1. USA | Law (1) | ‘Epigenetics does not displace the chromosome as the primary mechanism of biological inheritance. It does, however, challenge genetic determinism and the dichotomy between genes and environment. In the context of pregnancy and gestation, it challenges the dichotomy between form and matter, between genetic identity and the supportive environment of the womb.’ | Undefined | B6  B5 |
| Hendricks, J.S. (2016) [220] | 1. USA | Law (1) | ‘Sellers might take a few extra days, weeks or years to think about whether they want to provide sperm at all. These changes would pervasively alter the ultimate genetic identities of the resulting children. Different genetic people, in different numbers, and at different times would come into existence.’ ‘Guaranteeing existence also allows us to acknowledge the importance of genetic identity without taking genes to be definitive of the self. One could, for example, think of the conversants in the original position as souls awaiting genetic identity in order to be born.’ ‘The expected value solution, however, shows how we can logically take future children’s interests into account when choosing policies that will affect their genetic identities.’ | Undefined | B3  C3 |
| Hernandez, E.G. et al. (2015) [221] | 6. Mexico | Medicine (4) | ‘Amebiasis is an endemic disease and a public health problem throughout Mexico, although the incidence rates of amebic liver abscess (ALA) vary among the geographic regions of the country. (…) Therefore, we studied the association of the HLA-DRB1 and HLA-DQB1 alleles with resistance or susceptibility to ALA in two Mexican populations, one each from Mexico City and Sonora. Ninety ALA patients were clinically diagnosed by serology and sonography. Genomic DNA was extracted from peripheral blood mononuclear cells. To establish the genetic identity of both populations, 15 short tandem repeats (STRs) were analyzed with multiplexed PCR, and the allelic frequencies of HLA were studied by PCR-SSO using LUMINEX technology.’ | Undefined | A3i |
| Heuvel, K. Vanden (1999) [222] | 1. USA | Law (1) | ‘Jonathan also suggested that science has a partial answer: genetic typing to identify people by their individual DNA. He predicts (…) that genetic typing will increasingly be used to confirm the identity of computer users. Passwords, pin numbers, picture IDs, will disappear. This same genetic research, however, has also created the tool that eventually will negate genetic identity – the ability to replicate human beings. Thus, although genetic material may be a tool to verify identity, it will also undermine genetic typing as source of individual identity. Once an individual’s genetic material can easily be replicated to make a new individual, it will be human experience, as it is played out at different points along the time line, that will differentiate one individual from another.’ | Undefined | C1  D1 |
| Heyd, D. (1999) [223] | 5. Israel | Philosophy (3) | ‘Indeed, such a pre-embryo is a potential human being, but so are gametes before conception. It is true that conception creates the genetic individuation of humans, but this does not mean that it creates dignity or absolute worth. Dignity, as we have learnt from Kant, requires more than sheer genetic identity.’  ‘ (…) Analyzing man does not reduce the individual’s self-control once born. And of course human beings’ absence of self-control over their own genetic identity characterizes natural reproduction no less than the artificially regulated alternative. It is the logic of genethical decisions that the created has no autonomy over its own genetic makeup.’ | Undefined | C2  C1 |
| Heyd, D. (2001) [224] | 5. Israel | Philosophy (3) | ‘Recognizing the validity of wrongful identity claims may prove logically bizarre and legally disastrous, but with the growing control over the genetic identity of our children the issue of defending the alleged right of people to be born with ‘optimal identity’ will probably become more and more pressing.’ ‘They can in full logical coherence state that identity x is better for their child than identity y. And this applies both to genetic identity, sex, timing of birth, etc.’ | Undefined | C3 |
| Heyd, D. (2002) [225] | 5. Israel | Philosophy (3) | ‘The genetic identity, indeed the very existence of human beings, has been throughout history a matter on which human beings wielded only little power. Religious belief held it to be a matter of divine creation and design; scientific theory saw it as a matter of natural selection and evolution. Now, for the first time, human beings have achieved an impressive degree of impact over the genetic identity of plants, animals and potentially of human beings.’ ‘ It is logically incoherent to ground the opposition to radical changes in genetic identity of either individuals or groups of people on the rights of future people or on their good, since these may be different for them than they are for us. (…) In the absence of a rational argument for ethical constraints on the genetic manipulation of future people, we are left with a baffling permissive situation, according to which we may decide genetic issues purely on the basis of our own interests and values.’ | Undefined | C3 |
| Hibbert, M. (1999) [226] | 1. USA | Law (1) | ‘Iowa law defines ‘DNA profiling’ as ‘the procedure established.. for determining a person’s genetic identity.’’ | Undefined | D1 |
| Hicks, S.C. (1992) [227] | 1. USA | Law (1) | ‘But it is clear that genetic identity is not necessarily fixed by the mere penetration of a single sperm, because more than one may enter, and because even then some genetic material may be rejected. In fact, it is only at the completion of the process of fertilization with syngamy, that is, when the genetic material of the sperm and the egg have condensed into chromosomes to form a new genotype –a single cell- that genetic uniqueness can possibly be fixed.’ ‘Thus, the genotype of an individual may be different than that formed at fertilization, and genetic identity cannot be equated with the beginning or the end of individuality.’ | Undefined | C2 |
| Higginson, D.M. & Pitnick, S. (2010) [228] | 1. USA | Biology (4) | ‘Sperm are often considered to be individuals, in part because of their unique genetic identities produced as a result of synapsis during meiosis, and in part due to their unique ecology, being ejected away from the soma to continue their existence in a foreign environment.’ | Undefined | C1 |
| Hill, J.L. (1991) [229] | 1. USA | Law (1) | ‘It is beyond dispute that an important aspect of parenthood is the experience of creating another in one’s ‘own likeness.’ Part of what makes parenthood meaningful is the parent’s ability to see the child grow and develop and see oneself in the process of this growth. Through this process, the parent views himself or herself as a creative agent in nature. This genetic identity accords the parent a kind of limited, genetic immortality, which one commentator has called ‘the sense of living on through and in one’s sons and daughters and their sons and daughters.’’ | Undefined | A1  A2ii |
| Hill, W.G. (1993) [230] | 2. UK | Population Biology (4) | ‘Rasmuson (1993) computed the standard deviation (s.d.) of the genetic identity of relatives, i.e. of the proportion of the genome which is identical by descent.’ | Defined | A3ii |
| Holman, C.M. (2007) [231] | 1. USA | Law (1) | ‘The sixth genetics testing case, Promega v. Lifecodes, arguably does not involve human genes, since the patents do not cover protein encoding sequences, but rather specific genomic sequences useful in genetic identification, essentially ‘DNA fingerprints’ useful in forensics and paternity testing. Nevertheless, although some might characterize the patented sequences as ‘junk DNA,’ they are actually quite useful in genetic identity testing because they include variable number of tandem repeat (VNTR) sequences. Essentially, these regions contain a genetic sequence that is repeated multiple times, with the number of repeats varying between individuals. VNTR regions reside throughout the human genome, and by measuring the number of repeats at a number of different VNTR regions, it is possible to identify a specific individual with a high degree of certainty.’ | Undefined | D1  E6 |
| Holmes, R. (1997) [232] | 1. USA | Biology (4) | ‘The evolutionary biologist E. O. Wilson (Wilson 1975) (…) he has shown that each pinnacle is based on fundamentally different mechanisms. Human sociability and human altruism are based, not on genetic identity and chemical exchange (as in the colonial invertebrates), not on genetic relatedness and genetically programmed behaviors (as with the insects and their caste systems), nor merely on milk, animal communication, and memory (as with the other mammals), but rather on uniquely human speech and history. This means that human beings are connected more by story, and not so much by genes reproducing themselves.’ | Undefined | B1  A2ii |
| Honabach, D.R. (2003) [233] | 1. USA | Law (1) | ‘According to Dean Partlett, judicial recognition of a fiduciary duty running to the individual whose genetic identity is being ascertained will encourage the production of important genetic information because the individual will continue to be confident that genetic information garnered about her will not be exploited for ulterior purposes. Under Dean Partlett’s system, an individual would be able to bring a tort action for breach of confidence for the professional’s breach of her duty if the professional inappropriately discloses the individual’s genetic information.’ | Undefined | D1 |
| Hondius, F.W. (1997) [234] | 2. France | Politics (2) | ‘DNA analysis and testing enables the obtaining of complete and accurate knowledge of the human genome, I e the genetic identity of people including children yet to be born — and the biological relationship between them, as well as of the presence of defective genes and genetic risks.’ | Defined | E6  A2ii  B2 |
| Housman, D.E. (1995) [235] | 1. USA | Biology (4) | ‘The power of DNA fingerprinting has rarely been questioned when the presence of disparate VNTR bands effectively rules out a match between a suspect and the forensic specimen. It is the presence of matching bands that continues to elicit a swirl of controversy in the courtroom over the precise statistical power of the inferred genetic identity between the forensic sample and the accused person. Nevertheless, many geneticists would be more comfortable with an identification based on DNA fingerprinting than with one based on the recollection of a direct eyewitness.’ | Undefined | A2i  D1 |
| Hovenberg, H.W. et al. (1996) [236] | 2. Sweden | Molecular Biology (4) | ‘Likewise, no studies have given the gel-forming, subunit-based respiratory mucins a genetic identity, although a preliminary study has suggested that MUC5AC is one candidate. The aim of this investigation is to determine whether MUG2 and/or MUC5AC are present as large, subunit-based glycoproteins in airway secretions from individuals with healthy airways and patients with chronic bronchitis.’ | Undefined | F1 |
| Howett, M.K. (1994) [237] | 1. USA | Microbiology (4) | ‘Member of the Audience: For the genetic identity test, the RFLP test, are there standard sequences that are always cut out of the same chromosomes and compared for the same people?’ | Undefined | E1  D1 |
| Hoxha, E. (2015) [238] | 1. USA | Law (1) | ‘As explained here, gene expression is more complex than pure reliance on DNA sequence. Thus genetic identity and DNA sequence identity are distinct concepts. Reliance on DNA sequence identity alone as a test for determining the patentability of inventions is a gross oversimplification and an inaccurate interpretation of molecular biology.’ ‘DNA sequence is only one component of genetic identity.’ | Undefined | B6  E1 |
| Hsieh, A. (2004) [239] | 1. USA | Law (1) | ‘As the first population genetics project that will span multiple countries for the collection of DNA, the International HapMap has the potential to become an influential standard for the protection of population genetic information. The analysis highlights issues of the HapMap project and the national databases that raise ethical, social and legal concerns for the future. This Part provides various recommendations for such protections that account for not only the genetic identities of individual donors, but also community interests that will be implicated.’ | Undefined | D1  A3i |
| Hsu, C.C. et al. (2008) [240] | 5. China | Medicine (4) | ‘We found different genotypes for the complete hydatidiform mole (CHM), placenta and co-existing fetus derived from a single in vitro fertilized human oocyte by the analysis of short tandem repeat (STR) DNA markers. The molar tissue was found to be heterozygously androgenetic. The fetus and placenta contained identical maternal, but different paternal genomes. Two models were proposed to account for the identification of triple genetic identities in a single fertilized oocyte. (…) was fertilized by two haploid sperms, followed by tripolar spindle formation. Whatever is the mechanism, this case provides direct evidence that CHM can be derived from an oocyte containing an intact maternal genome.’ | Undefined | E6 |
| Hubins, J. (2001) [241] | 1. USA | Law (1) | ‘Defining gender based on chromosomal composition permits same-sex marriages to the extent that the public, not knowing of Christie’s ‘true’ genetic identity as a man, perceives Christie as a woman.’ | Undefined | B4 |
| Huddle, L.N. (2012) [242] | 1. USA | Medicine (4) | ‘This is the first report of a rare example of intracranial intraventricular twin fetuses in fetu for which a genome-wide single nucleotide polymorphism assay has confirmed their genetic identity.’ | Undefined | A2i |
| Ijiri, T.W. et al. (2012) [243] | 5. Japan | Molecular bioscience (4) | ‘In sexual reproduction, two gamete cells (i.e., egg and sperm) fuse (fertilization) to create a newborn with a genetic identity distinct from those of the parents. In the course of these developmental processes, a variety of signal transduction events occur simultan- eously in each of the two gametes, as well as in the fertilized egg/zygote/early embryo.’ | Undefined | C2 |
| Jablonski, N.G. (2004) [244] | 1. USA | Anthropology (2) | ‘Humans skin is the most visible aspect of the human phenotype. It is distinguished mainly by its naked appearance, greatly enhanced abilities to dissipate body heat through sweating, and the great range of genetically determined skin colors present within a single species. Many aspects of the evolution of human skin and skin color can be reconstructed using comparative anatomy, physiology, and genomics. (…) Because of its evolutionary lability, skin color phenotype is useless as a unique marker of genetic identity.’ | Undefined | A3i  A2ii |
| Jackson, E. (2008) [245] | 2. UK | Law (1) | ‘The fetus and the pregnant woman are not separate beings, but until birth the fetus is part of her body. It may have a distinct genetic identity, but that does not necessarily convert it into a separate rights holder. If it ever becomes possible, ectogenesis will mean that the artificially gestated fetus is an entity separate from its mother, and difficult questions about its legal status will have to be addressed. But, for a variety of reasons, we can be confident that most women will continue to gestate their fetuses inside their bodies, and for these women, the existence of a parallel method of gestation does not alter the basic biological fact that fetal life is so intimately connected to the pregnant woman’s body that decisions about what she does with her body should, as they are when she is in a non-pregnant state, be for her alone.’ | Undefined | C2 |
| Jackson, F.L.C. (2000) [246] | 1. USA | Anthropology (2) | ‘Today, as the post-genome era approaches, arbitrary and often capricious evaluations of Africans and African Americans still abound, as Du Bois had predicted. New paradigms such as ethnogenetic layering are proposed to disentangle cultural identity from genetic identity and provide an alternative to static, stereotype-dependent racial models of traditional anthropology and biology. Ethnogenetic layering has been applied in ecological risk assessment studies and has already revealed significant regional biodiversity (genetic substructuring) among African Americans.’ | Undefined | A3i |
| Jacobs, M.B. (2004) [247] | 1. USA | Law (1) | ‘Moreover, while there are compelling reasons for a child to know her or his genetic identity, that knowledge does not mean that it is in the child’s best interests to have an intact parent-child relationship terminated in the mere hopes of establishing a new one predicated on biology.’ | Undefined | A1  B1 |
| Jacobs, M.B. (2006) [248] | 1. USA | Law (1) | ‘Another reason proffered to base paternity on biology is a child’s entitlement to her genetic identity. Genetic identity forms a part of a child’s history. It is important, too, that a child know her genetic identity for medical history purposes.’ | Undefined | A1 |
| Jacobs, M.B. (2007) [249] | 1. USA | Law (1) | ‘Multiple parenthood permits children to benefit from maintaining relationships with long-term or intended caregivers and to have the ability to know their genetic identity and background.’ | Undefined | A1  B1 |
| Jaeger, C. (2017) [250] | 1. USA | Law (1) | ‘The biological, or genetic-identity, approach determines parentage  based upon who the biological parents of the child are or who is genetically related to the child.’ | Undefined | A1 |
| Jasanoff, S. (2006) [251] | 1. USA | Science and technology studies (5) | ‘Some U.S. courts have denied prisoners’ requests for post-conviction DNA testing on the ground that this would violate the law’s interest in finality, allowing technological progress to undermine legal settlements. (…) The social truth of what constitutes a family and what amounts to justice in the eyes of the law operates in these cases independently of scientific truths concerning human reproduction or genetic identity.’ | Undefined | D1  B1 |
| Javitt, G.H. (2003) [252] | 1. USA | Law (1) | ‘The author addresses legal issues arising from the technique of ‘blastomere separation.’ The author considers whether children created from this procedure have property rights over the other cloned embryos by virtue of their shared genetic identity and whether these rights could permit them to prevent the use of the remaining cloned embryos. She concludes that children created through this procedure do have property rights in their DNA code, and that these rights should permit the exclusion of others from using the remaining cloned embryos to produce additional children.’ | Undefined | D3  A2ii  C3 |
| Jenkins, N. et al. (2013) [253] | 2. UK | Sociology (2) | ‘Through these accounts, we develop the concept of the family corpus in order to highlight the role body networks play in shaping lay constructions of genetic identity and a familial disease biography.’ ‘Specifically, it has been suggested that the rise of genetic medicine has facilitated more somatic (Novas and Rose 2000) and interdependent (Kenen 1994) constructions of selfhood amongst those genetically at risk of disease. Thus, it has been argued that genetic identity is mphasizedon by a sense of embodied obligation (Howson 1998) or genetic responsibility towards others, where one’s own selfhood is defined in close relation to the selves of others (Hallowell 1999).’ ‘As such, we hope that the concept of the family corpus may advance future discussion of genetic identity, genetic responsibility and selves-in-relation by considering the role of bodies-in-relation, as well as how illness and self-identity may be experienced in ways that transcend individual bodies.’ | Undefined | B2  B1 |
| Jeong, K.H et al. (2015) [254] | 5. Korea | Orthodontics (4) | ‘The prevalence of hypodontia and microdontia was 3.55% and 3.00%, respectively. MZ had the highest PCR and RRR (13.0–15.3). The PCR and RRR values for both anomalies were much higher for DZ (5.0–11.9) than for siblings (1.4–2.6), despite the fact that DZ pairs and sibling pairs share 50% genetic identity.’ | Undefined | A2ii |
| Jiang, L. (2015) [255] | 2. UK | Law (1) | ‘Genon’s patent application contained human nuclei materials that possessed the characteristics of human cells. As claimed in the patent application, the invention is primarily used for the purpose of tissue or organ transplantation. If so, the invention could not exclude the possibility of developing into a human being. However, the committee did not ignore the possibility that the embryonic cells could exhibit the characteristics of an animal. In that situation, the method still violates public morality because it changes the genetic identity of a human germ line.’ | Undefined | C3  D3  E5 |
| Jiawen, E.L. (2016) [256] | 2. UK | Law (1) | ‘Identity can include genetic identity, encompassing the characteristics we get through our genetic make-up and how we perceive ourselves. Then there is qualitative identity, which includes our physical and personality traits. They concluded that the presence or absence of mitochondrial disorders could affect multiple layers of one’s identity but that none of these were unique to the treatments. For example, in IVF with donor eggs, there are recent cases where the resulting child struggles emotionally to come to terms with the fact that she looks nothing like her birth mother and will never be able to find out who her egg donor is. As no identifying information on mitochondria donors will be given under the amended legislation, commentators worry that such children will face similar issues. In the rush to raise concerns about mitochondrial transfers it should also be noted that a person’s identity is influenced by a plethora of factors other than their conception and their genetic link to their parents.’ | Undefined | B1  A1  A2ii |
| Jivraj, S. & Herman, D. (2009) [257] | 2. UK | Law (1) | ‘Here, Wilson J, also decouples race/ethnicity and religion. He decides that the boy, a child of a Muslim mother and a Sikh father, should have his Sikh names changed to Muslim ones, but not by deed poll. He should be brought up as a Muslim, as that is the residential carer’s (the mother’s) community, and that also requires his circumcision. Wilson confidently states that:  ‘A child cannot be brought up in two faiths simultaneously so, admirable though Sikhism is, he cannot be brought up as a Sikh. That however in no way precludes his becoming aware of his Sikh identity.’ This ‘half-Sikh identity’, Wilson J concludes, is a ‘genetic identity’ which the mother was attempting to ‘re-write’. So, like Ward LJ’s judgment in Re P, the child in Re S is seemingly racially or ethnically Sikh through carrying his father’s ‘Sikh genes’ (as N was a Jew in Re P), but he can and should become a Muslim by faith, like his mother.’ | Undefined | B3 |
| Joerden, J.C. (2008) [258] | 2. Germany | Law (1) | ‘Chimbrids related activities may take place in many different forms and for  many different purposes. It is clear that they may in individual cases concern a wide spectrum of interests and values that are generally considered to deserve judicial protection. These include freedom of research and the improvement of scientific knowledge, protection of public health and safety, protection of the person (autonomy, privacy/ personal integrity etc), human dignity and the genetic identity and heritage of future human generations.’ | Undefined | C3  D1  B7 |
| Joh, E.E. (2006) [259] | 1. USA | Law (1) | ‘We leave traces —skin, saliva, hair, and blood- of our genetic identity nearly everywhere we go. Should the police be permitted, without restriction, to target us and to collect the DNA that we leave behind? In a growing number of instances, the police, unburdened by criminal procedure rules, seek this ‘abandoned DNA’ from criminal suspects in hopes of resolving otherwise unsolvable cases. Successful DNA matches of identity are virtually conclusive of guilt.’ | Undefined | D1 |
| Joh, E.E. (2011) [260] | 1. USA | Law (1) | ‘Criminals might also steal DNA to use the genetic identity of their victim, either to deflect police suspicion or simply to assume the victim’s genetic identity for illegal purposes.’ | Undefined | D1  D3 |
| Johns, R. (2013) [261] | 1. USA | Law (1) | ‘These stories demonstrate that at least some children conceived with sperm from an anonymous donor have a pull, or inexplicable desire, to be in touch with the other half of their genetic identity.’ | Undefined | A1 |
| Johnson, M. (1995) [262] | 1. USA | Philosophy (3) | ‘The distinction between genetic and developmental individuality highlights some rock-bottom facts about embryonic development: at successful fertilization, the newly conceived zygote contains a genetic code distinct from that both of the mother and of the father, but it can, early in its development, become many (i.e. non-individual) (…)The genetic identity of the zygote is no guarantee that a single organism will develop, and since the traditional account of God’s infusion of the rational soul requires it to be the form of a single, determined body, it seems that the soul’s infusion cannot take place until the preembryo is irreversibly individual, around day 14 after conception.’ | Undefined | C2 |
| Johnston, J. (2003) [263] | 1. USA | Bioethics (3) | ‘Resisting a Genetic Identity: The Black Seminoles and Genetic Tests of Ancestry’ | Undefined | A3i |
| Jones, D & Wakely, J. (2008) [264] | 1. USA | Evolutionary Biology (4) | ‘We investigate the probabilities of identity-by-descent at three loci in order to find a signature which differentiates between the two types of crossing over events: recombination and gene conversion. (…) Although recombination and gene conversion do make different predictions, the differences are not likely to be useful in distinguishing between them using three locus patterns between pairs of DNA sequences. This implies that measures of genetic identity in larger samples will be needed to distinguish between gene conversion and recombination. | Undefined | A3ii |
| Jones, E.M. (1969) [265] | 1. USA | Law (1) | ‘And even in the case of identical twins there is no doubt, as I shall have occasion to develop later, that genetic identity does not make for identity of individuality, because, in fact, genetic traits, contrary to what people assume, do not determine what a person will become in the course of his life. Genetic endowments only act in such a manner as governing, conditioning the responses of that person to the environmental forces that infringe on him in the course of his development. And since environmental forces are never the same for two persons, even identical twins turn out to be different persons.’ | Undefined | B6  B1  C1 |
| Jordaan, D.W. (2002) [266] | 7. South Africa | Law (1) | ‘Intra-generational cloning is when the individuals who share the same genetic identity are born in the same generation, while inter-generational cloning refers to the situation where such individuals are born in different generations.’  ‘Does there exist a personal right to genetic uniqueness? Or, formulated in the words of the European Parliament quoted above, does each person have a right to his or her own genetic identity?’ ‘This brings me to the assumption on which the putative right to a unique genetic identity is based, namely that genetic uniqueness is an essential part of individuality. Studies of monozygotic twins clearly demonstrate that while people who share the same genetic identity may be very similar in many respects, they nevertheless differ sufficiently to leave no doubt about their individual identity.’ | Undefined | C1  A2i  B6  B1  C3 |
| Jordaan, D.W. (2005) [267] | 7. South Africa | Law (1) | ‘*While the egg and sperm are alive as cells, something new and alive in a different sense comes into being with fertilization ... there exists a new individual with its unique genetic identity, fully potent for the self-initiated development into a mature human being ... Any honest biologist must be impressed with these facts*.’  ‘Especially in the debate surrounding human cloning, much emphasis is placed on unique genetic identity. I must remark that the high value attached to unique genetic identity by some is evidence of genetic reductionist tendencies – a danger to a clear understanding of the human personality, which includes concepts like identity and individuality. (…) if an embryo has a monozygotic twin, it, simply put, *lacks* a unique genotype. (…) Since all pre-embryos do not possess a unique genotype, it cannot serve as a general criterion to differentiate between gametes and pre-embryos.’ | Undefined | C2  B5  C1  C3 |
| Juengst, E.T. (1999) [268] | 1. USA | Bioethics (3) | ‘Moreover, if access to the database is opened as widely as the range of research authorized in Alabama suggests, there is another risk from which sample anonymity offers no protection: the risk of being discovered to be a convicted criminal by any ‘researcher’ who already knows your genetic identity and finds you in the database.’ | Undefined | D1 |
| Juras, A. et al. (2017) [269] | 3. Poland | Anthropology (2) | ‘While having been extensively studied by archaeology, very little is known about their genetic identity*.* To fill this gap, we analyzed ancient mitochondrial DNA (mtDNA) from Scythians of the North Pontic Region (NPR) and successfully retrieved 19 whole mtDNA genomes. We have identified three potential mtDNA lineage ancestries of the NPR Scythians tracing back to huntergatherer and nomadic populations of east and west Eurasia as well as the Neolithic farming expansion into Europe. One third of all mt lineages in our dataset belonged to subdivisions of mt haplogroup U5.’ | Undefined | A3i  E3 |
| Kahn, J. (2003) [270] | 1. USA | Law (1) | ‘As genetics discourse expands, genetic identity is emerging as a new manner for assigning legal rights and duties. ‘ ‘And yet, even to the extent that this model values the human as a consumer of genetic products, it constructs genetic identity and status primarily as a function of defects to be corrected. The identity of the genetic citizen-consumer is by definition imperfect, incomplete, or defective. It needs a genetic product to be better, whole, or normal. Moreover, it is implicitly the duty or role of genetic citizen-consumers to buy and employ a ‘useful’ genetic product that targets them. To refrain from doing so would both undermine the value imparted to the work of the scientists and businessmen producing the product and to accept, embrace, and be relegated to a status as somehow genetically inadequate. This model teaches that a person’s relationship to her genes matters primarily as a function of defects or pathology to be corrected through the consumption of genetic commodities.’ ‘Ironically, these arguments also locate genetic identity in the pre-political realm by asserting a relation between genetic material and the human subject that is broad and general-much like any other tie to nature. Such dignitary arguments seek to encumber genes with a natural human identity but fail to engage questions of the social or political relations between a person and her genes.’  ‘In her construction of genetic identity, ownership is resisted not only in the name of the human subjects who supply the genetic material but also in the name of all generations, past and future, who are implicated, and hence somehow present in the germline.’ | Defined | B2  B1  B5  D3  D1  E5 |
| Kahn, J. (2014) [271] | 1. USA | Law (1) | ‘Ironically, even as these models involve genetic identities forming the basis for new communal ties, they reproduce classically liberal conceptions of citizens as atomized individual consumers-making demands for particular goods and services.’ ‘The conditions under which such goods are provided are irrelevant for them. In contrast to the model of activist groups, such as the Genetic Alliance, who use genetic identities to enlist individuals into groups that can make claims on the state (or on corporations), Emanuel et al. use biological identity (as broadly conceived by Rose and Novas) to make claims on the individual.’ | Undefined | A2ii |
| Kalinksy, K. et al. (2011) [272] | 1. USA | Medicine (4) | ‘Available paired tissue samples from breast tumors known to harbor mutations underwent massARRAY genotyping (n = 70) to identify PIK3CA and AKT1(E17K) mutations. Cores were macro-dissected from matched tissue, including normal breast, benign lymph nodes (LN), ductal carcinoma in situ, regional LN metastases, and distant metastases. Matched samples underwent genetic fingerprinting by multiple SNP genotyping to confirm genetic identity.’ | Undefined | F1 |
| Kamm, F. M. (2005) [273] | 1. USA | Philosophy (3) | ‘Suppose that (contrary to fact) the only way we could ensure holistic phenotypic nonidentity were by ensuring genetic identity. Then (if genetic identity did not occur naturally) cloning would be the preferred mode of reproduction, if all we were obliged to be concerned about was holistic identity differentiation. (…) Suppose that we have all been misled, and all our genetic makeups are already, in fact, naturally identical. If our phenotypic differences remain as they are, we would not worry about losing a holistic sense of identity differentiation.’ | Undefined | C1  B6  A2i  C3 |
| Kaneko, Y. Et al (2003) [274] | 5. Japan | Medicine (4) | ‘The major macromolecular components of mucus are mucin glycoproteins (mucins), which are large, highly glycosylated macromolecules with protein backbones encoded by MUC genes. (…) To our knowledge, however, the genetic identities of mucins secreted in the airways of patients with diffuse panbronchiolitis (DPB) have not been previously investigated, although hypersecretion is a common feature of this disease.’ | Undefined | F1 |
| Karakaxas, D. et al (2014) [275] | 3. Greece | Medicine (4) | ‘Pancreatic neuroendocrine tumors (PNETs) share a unique genetic identity, functional behavior, and clinical course. (…) The aim of this review is to summarize all recent advances of genetic research and new drug development in terms of PNETs, especially their genetic identity and subsequent alterations, leading to the development of near or total malignant behavior, and the new medical treatment strategies of this potentially curable disease on the basis of therapeutical agents acting where possible at the genetic level.’ | Undefined | F1 |
| Karpin, I. (2006) [276] | 4. Australia | Law (1) | ‘(…) why a new and unique genetic identity is key to embryonic status? It seems clear that genetic identity is here standing in for the individual. In this moment then a particular scientific account of selfhood as synonymous with DNA is being privileged over all others. This focus on the uniqueness of genetic identity accords with the observation by Dorothy Nelkin and Susan Lindee that DNA is ‘today taking on the social and cultural functions of the soul.’  ‘(…) In Australia being human in embryonic form means having a new and unique genetic identity. This moment of becoming embryonic necessarily evokes the further development of the foetus and, if brought to term, the child. (…) the embryo (now synonymous with a ‘new and unique genetic entity’) stands for all that it might become. Unhinged from the all-encompassing female body, and equipped with its own genetic identity, it attains an individuality that prefigures its birth.’ | Undefined | C2  B5 |
| Karpin, I. & O’Connell, K. (2002) [277] | 4. Australia | Law (1) | ‘Novas and Rose would (…) argue for instance that biomedical identity is just one amongst many identity claims. Identities they argue are ‘plural and multiple’ and ‘genetic identity is rarely hegemonic’. They suggest that the social relation of the family is another web of connection that feeds into a larger array of interconnections linking us with others. *New connections are traced in terms of the genetic threads that connected one person with another. Genetic identity is revealed and established only within a web of genetic connectedness, which is overlaid upon a web of family bonds and family memories, with their burden of mutual obligations and caring commitments, and with all the ethical dilemmas they entail.’ ‘With the emergence of the genetically at risk person, genes themselves have been constituted as what Foucault (1982) might term an ‘ethical substance’ that one works upon in relation to the self (genetic identity, reproduction, health) and in relation to others (siblings, kin, marriage, children). Rather than seeing these practices of genetic subjectification in isolation, we suggest that they intersect with, and become allied to, contemporary norms of selfhood that stress autonomy, self-actualization, prudence, responsibility and choice.’* | Undefined | B1  B2  A2ii |
| Kass, A.A. & Kass, L.R. (1995) [278] | 1. USA | Literature, history (3) | ‘This leaves only the hard question: shall it be his family name or hers? A little reflection will show why, as a general rule, it should be his. Although we know from modern biology the equal contributions both parents make to the genetic identity of a child, it is still true to say that the mother is the ‘more natural’ parent, that is, the parent by birth.’ | Undefined | A1 |
| Kass L.R. (1997) [279] | 1. USA | Bioethics (3) | ‘Troubled psychic identity (distinctiveness), based on all-too-evident genetic identity (sameness), will be made much worse by the utter confusion of social identity and kinship ties. For, as already noted, cloning radically confounds lineage and social relations, for ‘offspring’ as for ‘parents.’’ | Undefined | A2ii  A1  B1  C3 |
| Katz, K.D. (1993) [280] | 1. USA | Law (1) | ‘Others have criticized the determination of legal motherhood on the basis of genetic identity on the ground that an emphasis on the ‘seed’ is an ancient male-conceit.’ | Undefined | A1 |
| Katz, K.D. (1997) [281] | 1. USA | Law (1) | ‘Many of these children are born using anonymous donor artificial insemination and have no way of learning the identity of their biological father. While many people believe that children born of artificial insemination have not been harmed by this gap in their self-knowledge and genetic identity, their voices and their bewilderment at what has been denied them are beginning to be heard.’ | Undefined | A1 |
| Keating, R. (2014) [282] | 2. Ireland | Law (1) | ‘Genetic identity in the wider scientific field of study is tied to nDNA. MtDNA gives the crucial ability of every cell to function by providing energy and although mtDNA is central in the good health of an individual, it is not decisive in terms of inheritable characteristics. Thus, an mtDNA contribution arguably does not have the ‘deterministic’ factor that the court spoke of.’ | Undefined | E2  E3  A2ii |
| Kelly, F. (2017) [283] | 4. Australia | Law (1) | ‘They have argued that donor anonymity denies them access to vital information about their genetic identity and medical history.’ ‘Thus, while we must continue to resist the erasure of the ‘kin-making work of gestation, labour, and family practices’ and the concomitant ‘glorification of genetic connections’, genetic essentialism is not endorsed by validating the assertion of some offspring that their genetic identity is an important component of their overall sense of self. In fact, the adoption of open disclosure laws is perhaps the best way in which to enable offspring to form their own understanding of genetic relatedness and to make their own choices about the role genetic information plays in the formation of identity.’ | Undefined | A1  B5  B1 |
| Kelly, M.B. (1991) [284] | 1. USA | Law (1) | ‘Further, fixation on genetic identity creates a paradox. We can identify the rightful position —a healthy, younger child in the family. We also know the rightful position did not come to pass because of the genetic mphasize’s negligence. The difference between the rightful position and the existing state of affairs is substantial and reasonably ascertainable. Yet we cannot assess the damages against the negligent party because we cannot find the right recipient. One child would be unjustly enriched and the other simply does not exist.’ | Undefined | C3 |
| Keough, W.J. (2003) [285] | 4. Australia | Law (1) | ‘*All of us have had the privilege of receiving a unique combination of genes from our parents. We delight in the knowledge that we have our own genetic identity. However, a child cloned from an adult would be denied this experience. Instead, the child would live in the shadow and expectations of his [or her] predecessor.’* | Undefined | C1  C3 |
| Keren, B. (2014) [286] | 2. France | Molecular genetics (4) | ‘SNP arrays can detect long contiguous stretches of heterozygosity (LCSH). LCSH have 2 main interests: 1) they can detect uniparental isodisomies (UPD); 2) they can detect genetic identity by descent.’ | Undefined | A2ii |
| Khan, F. & Kessler, L. (2013) [287] | 1. USA | Law (1) | ‘The challenges to the patents-in-suit raise questions of difficult legal dimensions concerning constitutional protections over the information that serves as our genetic identities and the need to adopt policies that promote scientific innovation in biomedical research.’ | Undefined | D1 |
| Khankhanian, P. et al. (2010) [288] | 1. USA | Medicine (4) | ‘(…) we report validated genome-wide relationships between genetic identity and human mate choice in 930 couples of European ancestry.’ ‘The existence of ancestral or ethnic stratification with characteristic HLA types may influence the degree of genetic identity between couples.’ | Undefined | A2ii  A3i |
| Kimel, C.W. (2013) [289] | 1. USA | Law (1) | ‘The nature and form of DNA further bolsters one’s reasonable expectation of privacy in the information contained in her genetic material, as opposed to on the surface of her fingertips. A person’s DNA is hidden inside her body’s tissue, encrypted in a code that scientists only comparatively recently began to crack. Indeed, a person’s genetic identity is so subtly expressed that one cannot know the contents of her own DNA profile without the benefit of scientific testing. Obviously, then, the embedded, encrypted nature of a person’s DNA differs dramatically from the familiar swirls that are visible on that same person’s fingertips.’ | Undefined | D1  E1 |
| King, P.A. (1978) [290] | 1. USA | Law (1) | ‘The third justification for criminal abortion statutes, the state’s interest in protecting prenatal life, was potentially the most complex. The medical and scientific data before the Court were inconclusive on all the details of fetal development, except for general consensus that a fetus has a separate genetic identity at or soon after conception.’ | Undefined | C2 |
| Kirby, M. (1994) [291] | 4. Australia | Law (1) | ‘Among the specific topics examined in Bilbao were the following implications of genomic research: (…) The identification of human beings by genetic testing and the legal aspects of using the genetic identity as a unique and universal human identifier, (…)’ | Undefined | D1 |
| Kiruki, J.K. (2005) [292] | 7. Kenya | Philosophy (3) | ‘The positive argument for conception as the decisive moment of humanization is that at conception, the new being receives a genetic code which differs from its mother’s; an indication of a unique and distinct ontological being. It is this genetic identity which determines his/her characteristics, which are the biological carrier of the possibility of human knowledge and wisdom, which makes him/ her a self-evolving distinct being.’ | Undefined | C2 |
| Kishore, R.R. (2003) [293] | 5. India | Medicine (4) | ‘We have so far been synonymizing parentage with origin without mphasize that parentage is only a social and emotional expression. Advancing biotechnology mandates separation of this component from the biological element which is only confined to genetic linkage. Our failure to reconcile with the emerging reality has led to the ethical miscarriage of concealment of genetic identity of the babies born out of donated gametes.’ | Undefined | A1  B1 |
| Klipstein, S. (2017) [294] | 1. USA | Medicine (4) | ‘This yearning to maintain the mysteries surrounding reproduction will likely temper the development of strategies to alter our genome and affect the *genetic identities* of our offspring.’ | Undefined | C3 |
| Klitzman, R. (2009) [295] | 1. USA | Medicine (4) | ‘Because genetic markers have been identified for diseases far more than for ancestry, genetic identities related to disease can potentially shed light on how individuals integrate genetic data into their views of themselves more broadly as well. Hence, this article focuses on markers related to disease, not ancestry per se.’  ‘Individuals have to decide not only whether to incorporate their condition into their sense of themselves, but also how— with what moral valence (i.e., as positive or negative). They wrestle to gauge whether to view this genetic identity as negative or neutral—and to what degree to do so. A few saw themselves as ‘mutants,’ ‘evolutionary errors,’ ‘mistakes,’ or ‘freaks of nature.’ They felt that they had a ‘bad gene’ or ‘flaw,’ and struggled to understand it, stumbling at times in seeking appropriate terms. The fact that a mutation can be viewed as tainted can impede construction or embrace of a genetic identity.’ | Undefined | B2 |
| Kluge, E.W. (2001) [296] | 1. Canada | Philosophy (3) | ‘Deliberately bringing about genetic identity can be ethically reprehensible if and only if, by being made genetically identical, the persons who are thus born are deprived of something that is their right: namely, the right to genetic uniqueness. As has already been indicated, the claim that there is such a right is of dubious validity.’ | Undefined | A2i  C1 |
| Knoppers, B.M. (1993) [297] | 1. Canada | Law (1) | ‘This stands in contrast to the possible use of genetic information by economic third parties or, even, that of the state with respect to the DNA profiling of the genetic identity of criminals ‘ ‘The proposed legislation seems to be based on the assumption that the ‘gene is the person’, that is, constitutive of genetic integrity while legislation on DNA forensics by the state relies on the myth that ‘gene denotes genetic identity’.’ | Undefined | D1  B5 |
| Knoppers, B.M. & Lebris, S. (1991) [298] | 1. Canada | Law (1) | ‘The few reports that discuss human genetics would permit somatic cell therapy. As mentioned, however, these reports are unanimous in prohibiting any germ line alteration (considering the actual state of the science). The reasons range from the need to protect the genetic patrimony from any alteration that would be transmitted to following generations, to the need to protect the perceived uniqueness of individual genetic identity.’ | Undefined | C1  C3  A2ii |
| Kogan, T.S. (2004) [299] | 1. USA | Law (1) | ‘For the harmonization requirement recognizes the preeminence of the transsexual’s sexual identity in determining the individual’s sex for marriage purposes. The requirement takes seriously the conclusion of medical evidence that a transsexual’s psychological sense of his or her sexual identity is unchangeable, and may override the individual’s genetic identity.’ | Undefined | B4 |
| Kohm, L.M. (2011) [300] | 1.USA | Law (1) | ‘Consider the three surviving children of Michael Jackson, who are each the product of ART (…) Children like the Jacksons have a cemented social identity but have little understanding of their own genetic identity.’ | Undefined | A1  B1 |
| Kolb, R. (1999) [301] | 1. USA | Theology (3) | ‘Parents establish the essential identity of their children; God has made them ‘responsible’ for these children in ways that no sibling or other caretaker—by definition—can be. Parents give the gift of life and determine the genetic identity of their progeny. They shape and form the life of these offspring through their loving care in ways that psychologists perceive to be unique.’ | Undefined | A1 |
| Kording, N.D. (2004) [302] | 1. USA | Law (1) | ‘However, because genetic testing will be mandated in so many paternity determinations and made available to cohabitating and marital fathers at the time of the child’s birth, the child’s sense of his genetic identity will usually coincide with his sense of identity from his parental relationships.’ | Undefined | A1  B1 |
| Kramer, T.R. (2015) [303] | 1. USA | Law (1) | ‘Supporters of genetic tests to determine parentage often argue that genetic identity, hereditary links, and physical resemblance to family members are important reasons to give legal parentage to those who are the genetic parents of the child.’ | Undefined | A1 |
| Kriari-Catranis, I. (1997) [304] | 3. Greece | Law (1) | ‘The theoretic basis of the prohibition varies: The need to preserve the uniqueness of individual genetic identity, (and, therefore, to protecting human autonomy), the inadmissible use of human embryos as reseach vehicle and the risks inherent in these kinds of experiments have been named as principal reasons for that attitude.’ | Undefined | C1 |
| Kriari-Catranis, I. (2002) [305] | 3. Greece | Law (1) | ‘The ‘nasciturus’ as well as the extracorporeal embryo enjoy the right to genetic identity, incorporated into the Constitution during the recent constitutional revision.’  ‘The genetic identity is to be understood as the genetic constitution of the individual, the inherited genetic pattern. The constitutional protection of genetic identity has the following consequences:  a. The protection of genetic identity in conjunction with the principle of equality forbids any form of discrimination, based on the genetic characteristics of the individual. The principle of non-discrimination is expressly foreseen in article 11 of the Oviedo Convention.  b. The protection of genetic identity safeguards the genetic unicity and the genetic integrity. | Undefined | D1  C1  C2  A2ii |
| Kriari-Catranis, I. (2003) [306] | 3. Greece | Law (1) | ‘The genetic identity is to be understood as the genetic constitution of the individual, the inherited genetic pattern.’ | Undefined | A2ii |
| Kriari-Catranis, I. (2003) [307] | 3. Greece | Law (1) | ‘The genetic identity is to be understood as the genetic constitution of  the individual, the inherited genetic pattern. The constitutional protection of genetic identity has the following consequences:  (1) The protection of genetic identity in conjunction with the principle of equality forbids any form of discrimination, based on the genetic characteristics of the individual. The principle of non- discrimination is expressly foreseen in article 11 of the Oviedo Convention.  (2) The genetic identity is to be protected against any intervention, aiming to limit the individual autonomy. The new provision safeguards genetic unicity and genetic integrity.  ‘If the intervention via xenotransplantation into the genetic identity serves therapeutic goals, then it should be allowed, in the frame to be defined by law.’ | Undefined | A2ii  D1  C1  C3  B2 |
| Kumar, S. (2013) [308] | 1. USA | Law (1) | ‘In 1997, the European Parliament issued a Resolution on Cloning, in which it ‘[s]tresse[d] that each individual has a right to his or her own genetic identity. The Vatican has likewise stressed the importance of genetic identity. In arguing against the genetic modification of humans, the International Theological Commission stated that ‘[t]he uniqueness of each human person, in part constituted by his biogenetic characteristics and developed through nurture and growth, belongs intrinsically to him.’’ | Undefined | C1  C3  B1 |
| Kunich, J.C. (2002) [309] | 1. USA | Law (1) | ‘Obviously, the new child would have a new brain, with no recollection of any experiences from the life of the first child, but the genetic identity of the two would be a powerful factor moving grief-stricken parents toward the cloning decision.’ ‘Because of the genetic identity of donor and child of cloning, there should be drastically diminished risks of rejection of the transplanted organ in these situations.’ | Undefined | A2i  C3 |
| Kurtz, P.M. (1996) [310] | 1. USA | Law (1) | ‘Details and critiques emerging ‘right to be born with a sound mind and body’ for undue ethical and legal burdens it places on parents. Proposes instead ‘right to familial attachment’ which is right to conceive and bear children with their given genetic identity without intrusion by state.’ | Undefined | C3  C1 |
| Lahti, R. (1991) [311] | 2. Finland | Law (1) | ‘Alongside the question, for example, of when human life begins, or of the  status of the foetus, it would seem that consideration is now being given to how we can protect human identity and integrity (including genetic identity or integrity) or human dignity from violations that may arise through the new technology and its applications.’  ‘When the concept of a human individual or a human person is closely tied to the concept of personal identity, we approach the borderline of genetic identity and the recognition of the fact that genetic manipulation endangers this. The technology of genetic engineering with human beings is significant from the point of view of the quality of life and not so much from the aspect of the existence of life, and insofar some forms of gene technology affect future generations.’ | Undefined | C2  B7  C3 |
| Landau, R. (1998) [312] | 5. Israel | Social Work (2) | ‘Donor assisted conception provides new opportunities for achieving parenthood but at the same time raises issues of secrecy, anonymity, and the management of the offspring’s genetic origins. As with adoption, the child’s right to a genetic identity is at stake.’ | Undefined | A1 |
| Langlaude, S. (2014) [313] | 2. UK | Law (1) | ‘However there was no benefit for the child in his names being formally changed by deed poll as this would contribute to a comprehensive elimination of his half Sikh identity and would be an attempt at rewriting his genetic identity. The court also accepted that the boy was going to be brought up in a Muslim environment and although the father did not wish him to be circumcised, the court would allow the mother to do so, the court saying that he could always be encouraged to respect his Sikh faith.’ | Undefined | B3  B1 |
| LaPorte, J.L. (2013) [314] | 1. USA | Law (1) | ‘Adoption, the court stated, ‘permits in one setting a resolution of the rights of all putative parents, the child’s right to be raised by fit parents, and the child’s right to complete information about his genetic identity, including the consequences to her of the medical history of the genetic parents.’’ | Undefined | A1 |
| Latham, M. (1997) [315] | 2. UK | Law (1) | ‘Article 2 of the 1994 Statute on donation and assisted conception therefore goes further than the English law and ensures complete anonymity to donors. This means that children born as a result of donated gametes would never be able to know their genetic identity or their natural parents.’ | Undefined | A1  D1 |
| Laufer-Ukeles, P. (2014) [316] | 1. USA | Law (1) | ‘The right to know one’s genetic identity has been included in the U.N. Convention on the Rights of the Child: ‘States Parties undertake to respect the right of the child to preserve his or her identity, including nationality, name and family relations.’’ ‘Psychologists and sociologists have attested to the importance of knowing one’s genetic identity.’ | Undefined | A1 |
| Lavoie, J. (1989) [317] | 1. USA | Law (1) | ‘While there are many varieties of cancers, the morphological changes that occur in the cells themselves change the genetic identity of the cell such that the body’s immune system no longer recognizes the cancerous cells as ‘self.’’  ‘The disclosed information should consist of simple, layman descriptions of how long the tissue will be kept ‘alive’ and of any plans to use the genetic information either in recombinant DNA work or in the creation of an immortal cell line. This disclosure not only protects people with religious or idiosyncratic concerns over the continued existence of their genetic identity after their death (…)’ | Undefined | F1  D1  D2 |
| Laybourn, S. et al. (2016) [318] | 2. UK | Human genomics (4) | ‘Indian subpopulations (Chenchu, Koya, and Lobana Sikh) were analyzed at the genetic level for 12 Alu polymorphisms. These markers were then utilized to establish levels of genetic identity between the Indian populations and more widely between the Indian populations and a European population.’ | Undefined | A2ii  A3i |
| Laycock, D. (2016) [319] | 1. USA | Law (1) | ‘I do not share the Greens’ view of these matters (…) But the Greens’ view is perfectly logical within its premises: A new and unique genetic identity is created at the moment of fertilization, not later, and the FDA-approved labels say that emergency contraception may sometimes work by preventing the fertilized egg from implanting in the uterus.’ | Undefined | C2 |
| Leat, N. et al. (2004) [320] | 7. South Africa | Biotechnology (4) | ‘Our research focuses on the development and implementation of genetic identity testing systems for use in sexual assault cases. (…) In sexual assault cases it is often difficult to separate the female victim’s profile from the rapist’s profile. Analysis of Y-chromosome markers overcomes this by generating male specific profiles.’ | Undefined | D1  E4 |
| Leckey, R. (2015) [321] | 1. Canada | Law (1) | ‘Despite the judgment’s dominant framing of the Pratten claim as a quest for genetic information, the identity, relational, and genealogical aspects-which are social, not merely genetic – are salient in the testimony. Elements in Pratten offer credence to a scholar’s judgment that genetic identity is ‘a meaningless concept,’ since it ‘does not describe what is missing if a donor’s name is not known’; it is not ‘DNA alphabet soup’ that ‘donor offspring are perceived to lack. Instead, donor offspring may want to know ‘information about the means of conception and the other people involved and their actions and motives,’ which has ‘nothing to do with genetics at all.’ Similarly, curiosity and desire for information cast ‘in terms of the child not knowing his or her genetic origins’ often bears more on ‘notions of the donor as a person.’ | Undefined | A1  B1  B5 |
| Lee, H.Y. et al. (2004) [322] | 5. Korea | Forensic medicine (4) | ‘(…) The increased knowledge concerning mtDNA obtained in this study is believed to offer a useful means of determining genetic identity due to increased mitochondrial DNA haplotype diversity, by allowing mtDNAs to be classified into several types of peak patterns.’ | Undefined | E3 |
| Lee, J.H. (2011) [323] | 1. USA | Neuroscience (4) | ‘Recent development of the optogenetic functional magnetic resonance imaging (ofMRI) provides a new impetus for the study of brain circuits by enabling causal tracing of activities arising from defined cell types and firing patterns across the whole brain. Brain circuit elements can be selectively triggered based on their genetic identity, cell body location, and/or their axonal projection target with temporal precision while the resulting network response is monitored non-invasively with unprecedented spatial and temporal accuracy.’ | Undefined | F1 |
| Lee, J.H. (2012) [324] | 1. USA | Neuroscience (4) | ‘Optogenetic functional magnetic resonance imaging (ofMRI) is a novel approach that combines optogenetic control of neural circuits with high-field functional MRI. (…) By combining optogenetic control with fMRI readout, neural activity arising from specific circuit elements defined by genetic identity, cell body location, and axonal projection targets can be monitored in vivo across the whole brain.’ | Undefined | F1 |
| Lee, J.H. et al. (2010) [325] | 1. USA | Neuroscience (4) | ‘We also show that optogenetic fMRI (ofMRI) allows visualization of the causal effects of specific cell types defined not only by genetic identity and cell body location, but also by axonal projection target.’ | Undefined | F1 |
| Lenoir, N. (1993) [326] | 2. France | Law (1) | ‘(…) that the prohibiting children who will be born as a result of in vitro fertilization with a third-party donor from discovering their genetic identity and their biological parents is an attack on the right to health of the child and to the full development of his personality; (…)’ | Undefined | A1 |
| Lewis, J.L. (2008) [327] | 1. USA | Law (1) | ‘Other scholars note the artificiality of creating life through cloning and frame their ethical arguments as a matter of human autonomy. They argue that cloning imposes a genetic identity through the deliberate, artificial creation of life in violation of human autonomy, defined as freedom from the arbitrary action of others. In other words, the clone is denied the randomness of genetic reshuffling and, thus, is denied a true identity.’ | Undefined | C1  C3 |
| Lewis, J.W. & Pollak, E. (1982) [328] | 1. USA | Statistics (5) | ‘Moments of the steady state frequency spectrum (probabilities of identity of samples of genes) are obtained for a subdivided population by using standard recursive identity by state calculations. These moments are used to obtain variances for some measures of genetic identity, including Nei’s normalized genetic identity (I) and genetic distance (-log,I).’ | Undefined | A3ii |
| Li, W.H. (1979) [329] | 1. USA | Population genetics (4) | ‘It is often more interesting to consider the correlation of heterozygosity and the (normalized) genetic identity simultaneously alone (Ohta, 1976; Chakraborty, than to consider either of them Fuerst and Nei, 1977).’ | Undefined | A3ii |
| Little, J.W. (1969) [330] | 1. USA | Law (1) | ‘Genetic identity is not enough, as Dr. Dubos reminds us, for even identical twins mature to be distinct personalities as a consequence of the different environment experienced by each of the maturing individuals. Therefore, recreating an identical copy of an adult human being would require not only duplicating the genotype but also duplicating the environment that shaped him, beginning at the moment of conception.’ | Undefined | A2i  B6  C3 |
| Liu, D. (2005) [331] | 2. UK | Law (1) | ‘China possesses unique genetic resources. There are 56 minority groups, many of which live in the remote isolated border regions such as Tibet and XinJiang. Geographical isolation has allowed each group to maintain its cultural and genetic identity over thousands of years. Inbreeding has resulted in a homogeneous genetic makeup within each group and a lack of emigration/immigration makes it easy to construct large family pedigrees. This unique resource offers huge potential for the study of genetic diseases through linkage analysis in large numbers of families.’ | Undefined | A3i |
| LoBionde, A.R. (1991) [332] | 1. USA | Law (1) | *‘(…) should consist of simple, layman descriptions of how long the tissue will be kept ‘alive’ and of any plans to use the genetic information either in recombinant DNA work or in the creation of an immortal cell line. This disclosure not only protects people with religious or idiosyncratic concerns over the continued existence of their genetic identity after their death, but also will give people a heightened feeling of being included in all aspects of their medical treatment*.’ | Undefined | D1  D2 |
| Lombardo, P.A. (1996) [333] | 1. USA | Law (1) | ‘How does the existence of a patient’s genetic identity as a part of the medical record fit into this tradition of confidentiality? Physicians also perceive as novel the risks to medical privacy that the new genetics exacerbates.’  ‘The more recent history of genetic discrimination is exemplified by the XYY  controversy, where a chromosomal abnormality formed a basis for designating certain men ‘congenital criminals’ whose ‘supermale’ genetic identity supposedly marked them as prone to crime, and screening programs to deter criminality were proposed.’ | Undefined | D1  B4 |
| Long, L.L. (1982) [334] | 1. USA | Law (1) | ‘Should he initiate a paternity petition in a separate proceeding on the child’s behalf (…) all with the purpose of procuring essential child support, a psychological and genetic identity for the child, and another potential custodial parent?’ | Undefined | A1 |
| Lorenzi, P.L. et al. (2009) [335] | 1. USA | Genomics and bioinformatics (4) | ‘The National Cancer Institute’s NCI-60 cell line panel, the most extensively characterized set of cells in existence and a public resource, is frequently used as a screening tool for drug discovery. Because many laboratories around the world rely on data I NCI-60 cells, confirmation of their genetic identities represents an essential step in validating results from them. Given the consequences of cell line contamination or misidentification, quality control measures should routinely include DNA fingerprinting. We have, therefore, used standard DNA microsatellite short tandemrepeats to profile the NCI-60, and the resulting DNA fingerprints are provided here as a reference.’ | Undefined | F1 |
| Loureiro, J.C.S.G. (1998) [336] | 3. Portugal | Law (1) | ‘Article n. 1 guarantees the protection of the ‘human being in its dignity and identity’ (…) Also the protection of identity can be invoked for the prohibition of cloning. It is true that the person is much more than his genes and that genetic identity does not imply a personal identity, but that one is an important base contribution for this one.’ | Undefined | B1  C3 |
| Love, S.H. (1993) [337] | 1. USA | Law (1) | ‘Even with the present technology, however, the donor has an interest in the content of the DNA analysis because DNA test results provide evidence of identity. Moreover, even present day genetic identity testing represents a greater intrusion into personal privacy than do the alcohol and drug-use testing at issue in the Supreme Court cases creating and applying the ‘special needs’ test.’ | Undefined | D1 |
| Low, W.C. et al. (2007) [338] | 2. UK | Neuropathology (4) | ‘Several hereditary small vessel diseases (SVDs) of the brain have been reported in recent years (…). These disorders have invariably been suggested to be cerebral autosomal dominant arteriopathy with subcortical infarcts and leucoencephalopathy (CADASIL) but their genetic identities remain unknown. We used molecular, radiological and neuropathological methods to characterize these disorders. Direct DNA sequencing unexpectedly confirmed that affected members of the English family carried the R141C mutation in the NOTCH3 gene diagnostic of CADASIL.’ | Undefined | F1 |
| Lugosi, C.I. (2004) [339] | 1. USA | Law (1) | ‘The following list represents many of these artificial boundaries, which correspond to physical, psychic and social development of the unborn at various stages of human development. They include:  1. Moment of conception (assignment of genetic identity)…’ | Undefined | C2 |
| Lugosi, C.I. (2006) [340] | 1. USA | Law (1) | ‘The following list represents many of these artificial boundaries, which correspond to physical, psychic and social development of the human organism at various stages of development. They include:  1. Moment of conception (assignment of genetic identity)…’ | Undefined | C2 |
| Lupton, M. (2017) [341] | 4. Australia | Law (1) | ‘The prevalence of identity theft is a growing problem which might discourage many from reducing their ‘genetic identity’ to a computer file which could be stolen and misused. For example, such theft could result in unauthorized testing or disclosures that may lead to stigma and discrimination. The information on the file could also be used to determine identity or kinship relationships that the owner would prefer not to reveal in order to avoid personal embarrassment or legal actions.’ | Undefined | D1  D3 |
| Macgregor, C. (2012) [342] | 4. Australia | Anthropology (2) | ‘One way to engage with genetic knowledge as expressed in these two books is to consider the social and political context in which genetics are enacted as biosocial practices. Both studies illuminate that genetic identities are far from being centred on a stable bounded biological body, but rather concern a biosocial body surrounded by a plurality of genetic practices and discourses that are being reconfigured in social relations.’ | Undefined | B1 |
| Machulla, H.K.G. et al. (2002) [343] | 2. Germany | Medical immunology (4) | ‘Allele frequencies were obtained by direct counting. Hardy– Weinberg equilibrium, observed and expected homozygosity, Wright’s FIS for heterozygosity excess or deficit, Ewens-Watterson’s F statistics (for neutrality), Nei’s unbiased measure of genetic identity/distance (11) and Burrow’s two-locus linkage disequilibrium (LD) measures were estimated on PopGene v. 1.31 freely available on the Internet.’ | Undefined | A3ii |
| Macintosh, K.L. (2013) [344] | 1. USA | Law (1) | ‘Like the authors of Donum Vitae, Senator Brownback believes that the history begins at conception because that is the moment at which the unique genetic identity of an individual is created.’ | Undefined | C2 |
| Mack et al. (2002) [345] | 1. USA | Medicine (4) | ‘Like other first-degree relatives, dizygotic co-twins of breast cancer cases are at higher than usual risk (standardised incidence ratio (SIR)=1.7, CI=1.1 – 2.6), but the additional cases among monozygotic co-twins of cases are much more numerous (…) than the 100% genetic identity would predict.’ | Undefined | A2i  B2  B6 |
| MacKellar, C. (2017) [346] | 2. UK | Human Bioethics (3) | ‘Had the EU Commission brought the UK 2015 Regulations concerning MST and PNT to the European Court of Justice, however, it would have been interesting to see what would have resulted. Indeed, this would have clarified the following questions: (…)  4) Can the use of MST and PNT be considered as gene therapy taking place on a subject since MST and PNT are being used to bring into existence a subject and not to modify an already existing subject’s germline genetic identity?  5) What is the exact definition of a ‘subject’s germ line genetic identity’?’ | Undefined | C3  E5 |
| MacLeod, R.A.F. et al. (1997) [347] | 2. Germany | Cell cultures (4) | ‘During routine authentication checks, we noticed untoward karyotypic similarities between late-passage stocks of the Dami megakaryocyte and HEL erythroleukemia cell lines. Genetic identity of Dami with HEL was demonstrated by DNA fingerprinting with a (gtg)5 multilocus probe and confirmed for earlier passages of Dami deposited by its originators with the ATCC.’ | Undefined | F1 |
| Madden, D. (1999) [348] | 2. Ireland | Law (1) | ‘The psychological concerns which touch on the child’s need to know its father and its genetic identity (which arise in relation to donor insemination) would not arise here as the child would not need to question its identity, having access to the same information as a child conceived through normal sexual intercourse to a married couple.’ | Undefined | A1 |
| Maitland, N.J. et al (2001) [349] | 2. UK | Cancer research (4) | ‘To augment the currently available models of human prostate cancer in vitro, we have established extended life-span epithelial cultures from biopsies of well-differentiated prostate cancers. The genetic identity of the target cells was assessed by allelotyping, using microsatellites located on chromosome 8p, and microdissection of tissues and primary cell cultures.’ | Undefined | F1 |
| Malek, J. (2008) [350] | 1. USA | Medical Humanities (3) | ‘ In other words, when a potential parent chooses to use RGTs to conceive a child, that decision changes the genetic identity of the child conceived. The child who would otherwise have been conceived, therefore, is no better off as a result of the potential parent’s choice because, due to that decision, he or she does not exist. Even if one is persuaded by this argument, the Strong Claim can still be defended on the grounds that it promotes the well-being of future children. Rather than claiming that the use of RGTs promotes a particular child’s well-being, it can be claimed that the use of these technologies promotes the well-being of future children as a group . Understood this way, potential parents can make the class of future children better off by using RGTs to prevent disability.’ | Undefined | C3 |
| Manning, P.J. (2004) [351] | 1. USA | Law (1) | ‘As a result, not only have intended children been deprived access to information about their origin, they have also been deprived of fit parents, stable determinations of parentage, and information about their medical history and genetic identity.’  ‘Adoptees’ rights to genetic identity have only recently been recognized, and sperm donor children have only recently begun to demand those same rights.’ | Undefined | A1 |
| Marks, S.P. (2002) [352] | 1. USA | Law (1) | ‘Evelyn Shuster supports the existential sense of identity when she affirms that cloning threatens ‘rights to personal identity, individuality, and uniqueness.’ *Genetic independence comes... from a unique genetic identity. Because it makes impossible the child’s genetic independence, cloning holds that child genetic prisoner of another person’s genome. The child is robbed of the freedom to become who he/she is... the one unique person who lives and dies .... In short, cloning violates the child’s right to an open future.’* | Undefined | C1  C3 |
| Marques, C.L. (2000) [353] | 6. Brazil | Law (1) | ‘The Brazilian Constitution of 1988 offers greater protection for the rights of privacy and identity. I can think of nothing more fundamentally private and deserving of protection than the historical and genetic identity of a child. Historically, however, Brazilian family law has considered the secrecy of the origin of the child as an important element for the integration of the child in the new family. As a consequence, Brazilian adoption law generally does not provide for the adoptive child’s right to know his or her own origin.’ | Undefined | D1  A1 |
| Marteau, T.M. & Weinman, J. (2005) [354] | 2. UK | Health psychology (2) | ‘Since the cognitive representation of a threat activates coping procedures that fit with that representation, we also explore the proposition that cognitive representations of a threat that has a genetic identity are less likely to activate coping procedures that include risk-reducing behaviours. For example, using DNA risk information to assess an inherited predisposition to heart disease increased the extent to which the condition was seen as caused by genes, which in turn reduced the expectation that a behavioural means of coping would be effective (eating a low fat diet), but increased the expectation that a biological means was effective (taking lipid lowering medication).’ | Undefined | B2  B1 |
| Martin, P.A. & Lagod, M.L. (1998) [355] | 1. USA | Law (1) | ‘The tools of biotechnology have made it possible to isolate and maintain cell cultures in a laboratory, to fuse different kinds of cells, and to alter and then clone cells’ genetic material. New commercial products can be made with these methods. However, difficult issues arise when human cells or genes are involved. From the perspective of the medical patient or research subject, these issues concern the individual rights affected by the commercial exploitation of an individual’s unique genetic identity.’ | Undefined | D3 |
| Martin, P.A. & Lagod, M.L. (1990) [356] | 1. USA | Law (1) | ‘Reproduction begins with fertilization. Over a period of at least twenty-four hours, the sperm penetrates the egg and ‘two cells fuse to become one.’ The resulting one-cell ‘zygote’ has a unique genetic identity or ‘genome’ derived from the genetic material of egg and sperm.’  ‘The preembryo may have a unique genetic identity but it lacks the more developed ‘cluster of features’ which we associate with persons.’ ‘Therefore, Grobstein argues that a preembryo is entitled to ‘special concern’ if it has a chance to realize its highest potential as a full person. Further, even if that potential does not exist, Grobstein maintains that ‘the value of the preembryo as a member of the human community should still be recognized and conserved.’ Given its genetic identity, the preembryo has ‘inherent kinship relationships’ and is part of the ‘hereditary web of the human family.’ | Undefined | C2  B7 |
| Martinez-Gonzales, L.J. et al (2016) [357] | 3. Spain | Genetics (4) | ‘Currently, the Guatemalan population comprises genetically isolated groups due to geographic, linguistic and cultural factors. For example, Mayan groups within the Guatemala population have preserved their own language, culture and religion. These practices have limited genetic admixture and have maintained the genetic identity of Mayan populations.’ | Undefined | A3i |
| McConkie-Rosell, A. & DeVellis, B.M. (2000) [358] | 1. USA | Medical genetics (4) | ‘Sylvia Schild coined the term genetic identity to describe that dimension of  self-concept that develops from the individual’s perception of his or her inherited endowment (Schild, 1966). Schild hypothesized that satisfaction with one’s genetic endowment could lead to an enhanced self-concept, but discovering an inherited problem in the family could lead to a diminished self-concept. Schild (1981) also suggested that learning new information regarding genetic identity could have negative connotations resulting from the perception of one’s carrier status or that of others as being ‘inconvenient, discomforting, restrictive, or demanding in nature’ (p. 137). Because of an altered genetic identity, Schild (1984) asserted that the diagnosis of genetic disorder could ‘compromise seriously the sense of self-adequacy and ego identity needed to make a positive psychological adaptation’ to the diagnosis (p. 108).’  ‘Despite the fact that the term genetic identity was coined over 30 years ago, a literature review did not reveal any studies measuring this construct. However, several studies did measure self-concept or self-perception as the major outcome variable. The conclusions I research have been conflicting.’ | Defined | B1  B2 |
| McConkie-Rosell, A. et al. (2008) [359] | 1. USA | Medical genetics (4) | ‘The term, genetic identity was first used by Schild [1981] to describe a shattered self-adequacy syndrome caused by the knowledge that one possesses a defective gene. Findings from this study did not support the concept that knowledge of an altered gene was experienced as a threat to the total person resulting in a compromised sense of self. We did, however, find support for the concept that ‘‘genetic identity’’ exists. The girls who are carriers as well as the noncarriers do not appear to be experiencing their genetic identity as ‘‘I’m defective.’’ Rather, they are able to compartmentalize it as only a small part of their total self. (…) Our findings suggest that girls who are carriers and noncarriers are able to incorporate their genetic identity in a positive manner. These girls also acknowledge that their genetic status is uncontrollable. The recognition that genetic status is not controllable and thus not a personally accountable action, seems, for some of the girls, to be protective. This incorporation of the genetic identity and strategy of compartmentalization was not found in the girls who are at-risk.’ | Defined | B2  B1 |
| McCoy, J. (1993) [360] | 1. USA | Law (1) | ‘It also considered the ethical issue of patenting human beings. It recommended that the Proposed Directive clearly exclude inventions relating to the human being as such and which could modify the genetic identity of the individual as a whole.’  ‘In Article 2(3)(b), 139 the Commission revised Parliament’s Amendments to ensure that inventions that modify the genetic identity of human beings and that improve the lives of persons suffering from serious illness would be patentable.’ | Undefined | D3  C3 |
| McDaniel, M.J. (1998) [361] | 1. USA | Law (1) | ‘By definition, a clone would be related as an identical sibling to the individual who shares his or her genetic identity. This is true regardless of whether the clone is reared together with, apart from, or as a child of the individual who receives the clone’s tissue. If the convention’s language were interpreted based upon genetic identity, using a clone’s regenerative tissue to assist any individual who shares the clone’s genome could not be prohibited as a per se violation of human dignity.’ | Undefined | A2i  C3 |
| McDonald, E.S. (2003) [362] | 1. USA | Law (1) | ‘The embryo is a unique genetic individual. Fertilization determines his sex and genetic identity. Because the embryo contains ‘the entire genetic code of the individual,’ he is a distinct human being with a distinct identity. With predispositions to certain conditions such as heart disease, the embryo is ‘destined for a specific life.’ Fertilization sets the embryo ‘on a predetermined pathway of life.’’ | Undefined | C2 |
| McHughen, A. (2009) [363] | 1. USA | Philosophy (3) | ‘We’re all aware of the scourge of identity theft ravaging personal credit ratings and bank accounts. But a new, even more frightening, variant has evolved—genetic identity theft, with consequences perhaps even more dramatic and unsettling than financial losses because of the personal and intimate violation.’ | Undefined | D1  D3 |
| McKenna, A.T. (2013) [364] | 1. USA | Law (1) | ‘For example, in King, the police used a buccal swab inside a person’s cheek to obtain a DNA sample. While the method of retrieving the information is ‘quick and painless,’ the type of information collected is very intrusive, providing the government with access to one’s genetic identity, genetic markers, and family genetic history.’ | Undefined | D1 |
| McWhinnie, A. (1998) [365] | 2. UK | Social Work (2) | ‘The relevant sections of the 1989 UN Convention on the Rights of the  Child are Articles 7 and 8. (…) The relevant phrases in Article 7 are, ‘as far as possible the right to know...his or her parents’ and in Article 8, ‘...the right of the child to preserve his or her identity’, if one were to include in the definition of identity genetic identity or genetic information.’ | Undefined | A1 |
| Meilaender, G. (1998) [366] | 1. USA | Theological ethics (3) | ‘Well worth one’s pondering is the fact that, as he puts it, by contrast with sexual reproduction, cloning ‘is essentially a conservative process.’ That is, sexual reproduction is always open to continuous innovation. Sex and death are closely related. We wither and die so that others—who are like us, yet different—can replace us in the cycle of life. By contrast, ‘plants that reproduce by cloning are more or less immortal.’? Looked at from this perspective, one might wonder whether cloning would reflect a deep unwillingness to live in faith and hope, a desire to take the future entirely into our hands and be its guarantor.’ | Undefined | C3  D2 |
| Meintjes-van der Walt, L. (2001) [367] | 7. South Africa | Law (1) | ‘This is called the match probability and describes the statistical probability with which a randomly selected person will have a DNA profile that matches the crime sample. This obviously requires knowledge of the frequency with which the alleles represented occur within a population. This calculation is of particular importance, as DNA typing cannot claim to prove without doubt the genetic identity of two samples.’ | Undefined | D1  A2i |
| Meireles, A.M. et al. (2007) [368] | 3. Portugal | Pathology and molecular immunology (4) | ‘Objective: Our aim was to characterize the molecular and genotypic profile of eight thyroid carcinoma–derived cell lines—TPG1, FB2, B-CPAP, K1, XTC-1, C643, 8505C, and Hth74—in order to use them as in vitro models of thyroid carcinogenesis. (…) To determine the genetic identity of the cell lines, we performed genotypic analysis. Main outcome: The panel of cell lines we have studied displayed activation of several oncogenes (BRAF, RAS, RET=PTC) and inactivation of tumor suppressor genes (TP53) known to be important for thyroid carcinogenesis. Two of the cell lines—TPG1 and FB2—shared the same genotypic profile, probably representing clones of an ancestor cell line (TPG1).’ | Undefined | F1 |
| Mejido, J.A. (2011) [369] | 1. USA | Law (1) | ‘Informed consent merely informs future participants of genomic studies that misuse of their genomic information is possible. (… ) A potential participant may fully understand all possible misuses of genomic information after submission, but decide that the benefits outweigh the costs. By contrast, a potential genomic study participant may look at the drawbacks and potentially disturbing misuses of his or her genetic identity and decide that the costs outweigh the benefits of submitting his or her DNA sample.’ | Undefined | D1 |
| Melanson, L. (1995) [370] | 1. Canada | Law (1) | ‘Opponents of this framework object to viewing the fetus as a tissue specimen of the mother. They assert that the fetus is not merely a mass of cells which form a part of a pregnant woman’s body, but is a separate human being with its own gender and genetic identity. Kathleen Nolan, for example, states that ‘[t]reating fetal cadavers under a model that approximates routine salvage cannot help but depreciate and objectify them’; a ‘fetus isn’t a kidney, even when we act as if it is ...’’ | Undefined | C2 |
| Merrill, R.A. & Rose, B.J. (2001) [371] | 1. USA | Law (1) | ‘A clone might feel his individuality or sense of self-worth diminished. Awareness of the life choices made by his genetic progenitor could seriously constrict the clone’s sense of freedom and convince him that he was fated for a certain destiny. If his progenitor was particularly successful, the clone could feel pressure to live up to that standard, a feeling experienced by many younger siblings but magnified in this instance by genetic identity.’ | Undefined | C1  A2i  C3 |
| Meyerson, D. (1999) [372] | 7. South Africa | Law (1) | ‘Let us begin with the scientific evidence. Although there is no precise moment of conception –fertilization is a process, not a moment- it is certainly true that within approximately twenty-four hours after the sperm has penetrated the ovum the forty-six chromosomes which go to make up a person’s unique genetic identity are present. But does the start of a unique genetic identity mark the start of the Constitution’s protections? That is the question with which the court was faced in the Christian Lawyers Association case, and McCreath J rightly pointed out that biological facts do not help us with its answer.’ | Undefined | C2  E1 |
| Might, C. (2001) [373] | 1. USA | Law (1) | ‘The DNA profile is an individual’s blueprint of his own genetic makeup, arguably more private than one’s social security number, and would seem to clearly constitute privacy information. (…) Even if no known genetic defects were discovered in the interpretation of the individual’s DNA profile, DNA technology is evolving rapidly so that the information which can be obtained and interpreted from a profile tomorrow will be very different from the information that can be gathered today, making the harm less speculative. There is a ‘substantial probability’ that the release of an individual’s DNA profile would ‘cause an interference with personal privacy.’ This interference is more than merely speculative and much more than simply a ‘piece of a puzzle’ of an individual’s identity. It is the individual’s genetic identity.’ | Undefined | D1  B2 |
| Millbank, J. (2012) [374] | 4. Australia | Law (1) | ‘ Indeed one MP evidenced the importance of genetic identity and the issue of ‘genealogical bewilderment’ through reading into the parliamentary record the entirety of one of her children’s favourite books, ‘Are You my Mother?’ In this light, it is a fair inference that members of parliament were informed about surrogacy to a considerable degree through media representations and popular or ‘folk’ understandings, rather than research findings.’ | Undefined | A1 |
| Miller, P.D. (2000) [375] | 1. USA | Theology (3) | ‘That our individuality cannot be reduced to a matter of genetic code, however, is evident from the exception to the rule, that is, the genetic makeup of identical twins. The shared genetic identity of identical twins does not mean two persons who are simply clones of one another. Personality, mind, spirit, and affects are shaped by many other things as well, and the significance of the genetic makeup must not be allowed to obscure the many other aspects of the human—both as a species and in our particularity—that belong to the work of the Creator and the answer to the psalmist’s question. Whether or not he views the matter at all theologically, Craig Ventnor, one of the two project directors in the genome decipherment race, said at the White House announcement that he learned out of his experience as a medic in Vietnam that the human spirit transcended the physiology that is controlled by the genome.’ | Undefined | A2ii  B6  B1  B3 |
| Modell, S. M. et al. (2014) [376] | 1. USA | Public health (4) | ‘Religious academicians have probed deeply into the lines of ownership, leaving some room for human and divine sanctity and room as well for arguments favoring gene patenting to promote health. Baruch Brody drew a dividing line respected by multiple sets of authors (Hanson 2002; Resnik 2001). Under the general principle of respect for human dignity, a procedure that co-opts or commodifies human identity is to be avoided. Though human identity is not equivalent to genetic identity (some sociobiologists might differ on this point), the entire human genome encroaches far enough on fundamental human identity to make it unethical to patent, Brody contends. Patenting parts of the genome (genes or shorter DNA sequences) would remain under consideration, though.’ | Undefined | D3  B7  E1 |
| Molhoek, B. (2012) [377] | 1. USA | Theology (3) | ‘In addition, cloning is viewed as illicit because it ‘would impose on the resulting individual a predetermined genetic identity, subjecting him—as has been stated—to a form of biological slavery, from which it would be difficult to free himself. The fact that someone would arrogate to himself the right to determine arbitrarily the genetic characteristics of another person represents a grave offense to the dignity of that person as well as to the fundamental equality of all people.’’ | Undefined | C1  C3 |
| Moore, A. (2003) [378] | 1. USA | Law (1) | ‘As the success rate of pregnancy through in vitro fertilization (IVF) rises, the question emerges as to the true genetic identity of these children. An increasing number of couples adopt gestational surrogacy as an option to conceive when they are unable to do so on their own.’ | Undefined | A1 |
| Moore, P. (2000) [379] | 2. UK | Medicine (4) | ‘In her study, all of the parents who had used donor insemination had opted not to tell their children about the treatment ‘Parents found the deception easy while the child was small but all ran into difficulties later on,’ she said. She pointed  out that people who discovered later in life that their parents had kept half of their genetic identity a secret were often extremely angry.’ | Undefined | A1 |
| Moreteau, O. (2008) [380] | 1. USA | Law (1) | ‘Dr. Laura Franciosi and Professor Attilio Guarneri, both of Bocconi University in Milan, raise the delicate question of the protection of genetic identity, at a time where so much information can be found in human DNA. Huge benefits may be derived for human society, yet at the expense of individual interests. The paper explores ways of protecting individual interests, using privacy (a technic that pertains to the person) as well as property paradigms. Though primarily based on American materials, this paper shows that taxonomy alone does not solve complex issues.’ | Undefined | D1  D3 |
| Morgan, D. & Nielsen, L. (1993) [381] | 2. Denmark | Law (1) | ‘It seems that the protective function concerning both children’s rights and ethical concerns leads to rather restrictive legislation in Scandinavian contrast to England (and USA/Canada). Thus the child is to some extent secured from birth:  - a right to have both a father and a mother;  - a right to be fostered by the genetic father and mother;  - a right to be born by the fostering mother;  - a right to know its origin (Sweden).  The ethical concerns are outlined in the reports from the Danish Council of Ethics, the Norwegian report NOU 1991:6 ‘Mennesker og bioteknolologi’ (Humans and biotechnology) and in the Swedish report ‘Genetisk integritet’ (Genetic identity). | Undefined | A1 |
| Moses, L.B. (2007) [382] | 2. UK | Law (1) | ‘Consider the example of ‘mother.’ Prior to the use of in vitro fertilization, a mother (other than in cases of adoption) was the woman who bore a child and contributed to its genetic identity. Following the introduction of in vitro fertilization, it became possible for the concept of ‘mother’ to fragment into at least two people: the woman contributing an ovum, and the woman gestating and delivering the child. Thus a legal rule giving custody of a child to its ‘mother,’ without further definition, becomes uncertain.’ | Undefined | A1 |
| Moyer, T.J. & Anway, S.P. (2007) [383] | 1. USA | Law (1) | ‘Four nitrogenous bases are found in DNA: adenine, cytosine, guanine, and thymine. It is the sequence of these bases in DNA that determines genetic identity; the sequence differs somewhat between individual members of a species and much more between members of distinct species.’ | Undefined | E1  A2ii |
| Mukherjee, M.B. et al (2009) [384] | 5. India | Hemato-genetics (4) | ‘The present study was undertaken to determine the extent of diversity at 12 microsatellite short tandem repeat (STR) loci in seven primitive tribal populations of India with diverse linguistic and geographic backgrounds. DNA samples of 160 unrelated individuals were analyzed for 12 STR loci by multiplex polymerase chain reaction (PCR). Gene diversity analysis suggested that the average heterozygosity was uniformly high ( >0.7) in these groups and varied from 0.705 to 0.794. The Hardy-Weinberg equilibrium analysis revealed that these populations were in genetic equilibrium at almost all the loci. (…) The cluster analysis and multidimensional scaling of genetic distances reveal two broad clusters of populations, besides Moolu Kurumba maintaining their distinct genetic identity vis-à-vis other populations.’ | Undefined | A3i |
| Muller, G.B. & Wagner, G.P. (1996) [385] | 1. USA | Biology (4) | ‘Genetic identity can indicate morphological identity in some cases, but several examples show that gene expression patterns and regulatory systems of development may be highly conserved while morphological characters undergo dramatic evolutionary innovation. This indicates some independence of structural homology from its genetic and developmental makeup. It is proposed that phenotypic evolution depends strongly on the epigenetic context in which genetic redundancy becomes available for the control of new developmental interactions.’ | Undefined | B6  A2ii |
| Mulligan, A. (2014) [386] | 2. Ireland | Law (1) | These cases demonstrate that genetic identity is a key element of the court’s understanding of identity. This is clear from Mikulic and from subsequent cases that deal with the establishment of paternity. The court’s focus was not on the existence of a social relationship between the plaintiffs and the biological father, but simply on the biological truth of the plaintiffs’ paternity and the importance of that to their personal development.’ ‘As such, the right is not related to the intent to rear, or to the social relationship between mother and child. It is solely about the importance of knowing one’s genetic identity as a part of the development one’s personality.’ | Undefined | A1  B1 |
| Munoz, H.S. & Fiodorova, A. (2014) [387] | 2. Netherlands | Law (1) | ‘In conclusion, it could be said that only ‘the right to privacy and the consistent rights to informative self-determination and genetic identity could be affected by the collection of DNA samples’. As these rights are closely related to data protection, they will be touched upon in Section 4.’ | Undefined | D1 |
| Murphy, E. (2016) [388] | 1. USA | Law (1) | ‘In an oft-quoted passage in Watson, the Court reasoned: Given that law enforcement ha[s] long been authorized to obtain the name and biographical information of the driver, the use of DNA to merely confirm the accuracy and completeness of that information poses no meaningful intrusion on liberty under the Fourth Amendment. A driver who wishes to protect his or her genetic identity can always choose to avail him or herself of alternative means of transportation or, more simply, abide by the traffic laws.’ | Undefined | D1 |
| Murphy, T.F. (2013) [389] | 1. USA | Medical education / Medicine (4) | ‘Changes at the genetic level will not always, either, alienate people from their own sense of self. On the contrary, genetic modifications can help amplify choice, enrich lives and consolidate identities. Ultimately, there is no moral requirement that people value their contingent genetic endowment to the exclusion of changes important to them in their future genetic identities. Through weighing risks and benefits, adults also have the power to consent to—and assume the risks of—genetic modifications for themselves in a way not possible in prenatal genetic interventions.’ | Undefined | C3  C1 |
| Nagai, A. et al. (2006) [390] | 5. Japan | Legal medicine / medicine (4) | ‘Sequence polymorphism of the hypervariable regions HVI, HVII and HVIII of mitochondrial DNA (mtDNA) was analyzed in a sample of 400 unrelated Japanese individuals living in Gifu Prefecture (central region of Japan) by PCR amplification and direct sequencing. A total of 308 different haplotypes resulting from 187 polymorphic positions was found in our Japanese population sample. The most common haplotype was shared by 10 individuals. The genetic diversity and the genetic identity were calculated to be 0.9975 and 0.0050, respectively.’ | Undefined | A2i  A3i  E3 |
| Nagai, A. et al. (2003) [391] | 5. Japan | Legal medicine / medicine (4) | ‘Sequence polymorphisms of the hypervariable region HV1 in mitochondrial DNA (mtDNA) were analyzed in a sample of 137 unrelated Japanese individuals living in Gifu Prefecture (central region ofJapan) using polymerase chain reaction amplification and direct sequencing. Eighty-two different haplotypes resulting from 81 variable sites were found in the mtDNAHV1 region between positions 16061and 16450. The most frequent haplotype (16223T, 16362C) was shared by ten individuals. The genetic diversity and the genetic identity were 0.985 and 0.022, respectively. | Undefined | A2i  A3i  E3 |
| Naglyaki, T. (1984) [392] | 1. USA | Biophysics and theoretical biology (4) | ‘A model for the evolution of the probabilities of genetic identity within and between loci of a multigene family in a finite population is formulated and investigated.’ | Undefined | A3ii |
| Naglyaki, T. (1988) [393] | 1. USA | Molecular genetics (4) | ‘The evolution of the probabilities of genetic identity within and between the loci of a multigene family is investigated. Unbiased gene conversion, equal crossing over, random genetic drift, and mutation to new alleles are incorporated.’ | Undefined | A3ii |
| Naglyaki, T. (1990) [394] | 1. USA | Ecology and Evolution (4) | ‘The evolution of the probabilities of genetic identity within and between the loci of a multigene family dispersed among multiple chromosomes is investigated.’ | Undefined | A3ii |
| Naglyaki, T. & Barton, N. (1986) [395] | 1. USA | Molecular genetics and cell biology (4) | ‘The evolution of the probabilities of genetic identity within and between tandemly repeated loci of a multigene family is investigated analytically and numerically. Unbiased intrachromosomal gene conversion, equal crossing over, random genetic drift, and mutation to new alleles are incorporated.’ | Undefined | A3ii |
| Nash, D. (2002) [396] | 2. UK | Law (1) | ‘However, human cloning is also certain to have novel and profound legal ramifications on many non-medical aspects of society. These include the uncertain rights of cloned individuals, genetic donors, and donors of ova, expansion of the wrongful birth doctrine, as well as the emergence of the new crime and possible tort of ‘genetic identity theft.’’ | Undefined | D3 C3 |
| Nathan, A. (2002) [397] | 1. USA | Rhetoric (3) | ‘It is by the enumeration of this list of types of individuals that Boisselier affirms the right of the individual to exist, as it were infinitely, even though the reproduction of these individuals through cloning does not in itself constitute their immortality on her view – she regards each human being as a unique individual regardless of its genetic identity with another human being, and does not consider the death of an individual who has first been cloned to be any less of a death. In this sense the reproductive argument is for Boisselier not at all an instrumental argument but a moral, or a legal-philosophical, one. It involves the affirmation of an absolute right of the individual to make use of his or her own life and science to an unlimited extent.’ | Undefined | C1  C3  A2i |
| Neal, M.A. (1980) [398] | 1. USA | Religion (3) | ‘Altruism, the ideal of world religions—the disinterested love of the other, the ideal on which humanists have focused the classic tragedies—has potential to be realized only as it relates to the survival of the species. In this analysis, service to others or kindness toward them is limited to two functions—kin selection to extend genetic identity through the survival of the genetically related group, and reciprocity mechanisms involving collaboration with useful others in a quid pro quo exchange.’ | Undefined | A2ii |
| Nei, M. (1978) [399] | 1. USA | Population genetics (4) | ‘Theoretical works on Nei’s genetic distance and its extensions are discussed (…) Formulae for the *genetic identity* of genes at the electrophoretic level when the mutation rate varies from locus to locus are also presented.’ ‘(…) is the normalized identity of genes (or genetic identity) between X and Y.’ | Undefined | A3ii |
| Nelson, J.R. (1988) [400] | 1. USA | Theology (3) | ‘It is possible, then, to maintain a biblical view of human freedom and responsibility while acknowledging the power and significance of genetic coding. When scientists announce that the chemistry of DNA is so certain, universal and uniform that all forms of life on earth are essentially the same, a credulous public jumps to the conclusion that traditional claims for the uniqueness of the human species have been nullified. But the discovery of genetic identities between human cells and those of most other organisms does not negate the distinctiveness of human beings. Rather, it shows how the infinite creative power has used these marvelous mechanisms to fashion us as we are. Such knowledge also shows, to our sorrow, how dysfunctions, diseases and physical malformations occur within the scheme of life that the Creator intends as good.’ | Undefined | A2i  B7  B3 |
| Nelson, J.S. (2000) [401] | 1. USA | Theology (3) | ‘Religion enters into the center of the debate for Burhoe regarding how altruistic behavior is to be understood and accounted for. It is noted that whereas genetically related species exhibit a high degree of cooperation, the less genetic identity obtaining between individuals leads to a diminishment of cooperative activity. The scientific validity of religion rests on its being able to explain how altruistic behavior is possible given the significant lack of kin relationships in which cooperative behavior is evident. It is the function of religion as a value system to explain and motivate for cooperative behavior left unexplained by lack of family relatedness.’ | Undefined | A2ii  B1  B3 |
| New, J.G. (2006) [402] | 1. USA | Law (1) | ‘The genetic identity of K.M. as a parent was not in dispute, thus establishing her as a ‘biological parent.’’ Furthermore, like Lisa, K.M. had arguably met the standard of paternity by taking the child into her home and holding it out to the world as her own.’ | Undefined | A1 |
| Nishimaki, Y. et al. (1999) [403] | 5. Japan | Legal medicine (4) | ‘Usefulness of mtDNA sequencing for individual identification is estimated by indexes of genetic diversity and genetic identity based on the distribution of mtDNA sequence type. Genetic identity, which represents the probability of two randomly selected individuals having an identical mtDNA type, is more important for forensic examination.’ | Defined | D1  E3  A3ii |
| Nixon, C.S. (2002) [404] | 1. USA | Law (1) | ‘Reproductive cloning uses the donor’s cells with the intent to implant the resulting embryo in a uterus to create an individual with the same genetic identity as the donor. This method of reproduction might be favored by couples who are unable to conceive genetically related children or who are carriers of recessive diseases, such as Tay-Sachs, sickle-cell anemia, or cystic fibrosis. Reproductive cloning may also be used to create a child with preferred genetic traits like intellect or physical appearance.’ | Undefined | A2i  C3 |
| Noble-Allgire, A.M. (1999) [405] | 1. USA | Law (1) | ‘The arguments generally fall into a ‘nature’ versus ‘nurture’ debate: those who favor the genetic mother focus on the bonds of nature, i.e., the concept of genetic identity, while those who favor the gestational mother focus primarily on the social bonding that occurs between the child and mother during pre-natal and post-natal nurturing.’ ‘ A child’s genetic identity obviously is based upon the genetic code received from the egg and sperm providers. Thus, the genetic mother shares half of the genes that dictate the child’s physical attributes, including a predisposition to certain medical conditions, and perhaps certain mental or psychological attributes as well.’  ‘The importance of this genetic identity is illustrated by the desire of some adopted children to find their biological parents or separated siblings. (…) Some commentators have suggested that genetics is of ‘compelling importance’ to an individual’s sense of identity while the gestational mother plays only a minor role in developing the child’s identity.’ | Undefined | A1  B1  B2 |
| Nora, L.M. & Mahowald, M.B. (1996) [406] | 1. USA | Medicine (4) | ‘Noonan argues that a right to life begins at fertilization through conception by human parents. He says that genetic identity and species membership as human confers personhood. He does not distinguish between persons and humans. Noonan says that being human confers the same status and protection as that of a person, either because persons and humans are the same thing or because potential persons merit the same protection as actual persons.’ | Undefined | C2  B7 |
| Norton, V.G. (1994) [407] | 1. USA | Law (1) | ‘In making the distinction between the pre-embryo and the embryo, the Davis court rejected an argument that there is no distinction between the pre-embryo and embryo because the genetic identity of the embryo is determined at conception. The Davis court and other commentators have based such a distinction in part on the conclusion that the early pre-embryo is not genetically unique. At conception, when the sperm and ovum fuse, the resulting one-celled zygote contains the forty-six chromosomes which contain all the information necessary to direct the formation of a human being. Although the resulting pre-embryo may later split-while the cells are still pluripotent-to form genetically identical twins, the original embryo is still genetically unique in the sense that it will be the source of any genetically identical human beings. The chances against another individual independently possessing the identical genome are at least seventy billion to one.’ | Undefined | C2 |
| O’Donovan, K. (1988) [408] | 2. UK | Law (1) | ‘The issue of identifying genetic parents poses difficulties. This article has argued that it is not always possible to protect the interest that persons have in their genetic identity. However, the argument for the extension to the children of donation of rights enjoyed by the children of adoption is powerful. At the same time we should confront the contradictions of a culture which constructs identity on genes and yet permits children to be brought up on a separate, social basis. The separation of genetic and psychological identity puts individuals at risk in such a culture.’ | Undefined | A1  B1  D1 |
| O’Donovan, K. (2002) [409] | 2. UK | Law (1) | ‘We know from research on adopted children that genetic identity is important to some, whether for medical, psychological, or material reasons.’ ‘Open adoption policies contained in legislation in many jurisdictions show acceptance of blood ties as important, yet this investigation has not found that genetic identity is central in the discourses concerning abandonment under present investigation.’ | Undefined | A1 |
| Ohm, P. (2015) [410] | 1. USA | Law (1) | ‘Perhaps the fastest growing category of protected health information includes evidence of propensity to disease or disability, especially through genetic information. In 2008, Congress passed GINA, which disallowed discrimination on the basis of genetic information with respect to employment and health insurance. In passing GINA, lawmakers recognized that ‘discrimination based on a person’s genetic identity is just as unacceptable as discrimination on the basis of race or religion.’ The law was based on the notion that ‘[a] person’s unique genetic code contains the most personal aspects of their identity,’ and the law was a response to Americans’ ‘legitimate fears about how this deeply private information will be used.’’ | Undefined | D1  B2 |
| Onyango, G. (2010) [411] | 1. USA | Law (1) | ‘The right to be unique is already alluded to above, but by way of emphasis, it may be added that opponents claim that human cloning takes away the sense of uniqueness; that the clone is likely to be saddled with the genetic identity of its makers or ‘parents’ and that this may result in an identity crisis. But proponents of cloning are adamant that identity crisis has never been a problem with twins and they, therefore, wonder why it should be with clones. They say that though genes are the building blocks, what matters most is the environment in which one is brought up. They assert that it is the environment that shapes individuals.’ | Undefined | C1  C3  A2ii  B6  B1 |
| Orentlicher, D. (1999) [412] | 1. USA | Law (1) | ‘Other commentators invoked (4) the interest or even the right of people to not be deprived of their unique genetic identity and (5) concerns about the psychological effects on children of cloning from not having a biological father and mother in the way everyone else does.’ | Undefined | C1  A1  C3 |
| Papastathis, C.K. (2001) [413] | 3. Greece | Law (1) | ‘She admires the achievement, supports the thirst for new knowledge and deeply sympathises with the anticipation of revolutionary progress in diagnostic, preventive and therapeutic medicine. She glorifies the omniscient God for this gift, and prays and hopes that a more profound knowledge of our biological and genetic identity will facilitate the course toward spiritual self-knowledge, as well as the knowledge of God.’ | Undefined | B2  B3 |
| Park, D. (2010) [414] | 1. USA | Law (1) | ‘Relying on the established principle that courts ‘have declined to determine the economic value of the life of a person with disabilities as compared to having no life at all,’ the court found no legally cognizable claim because any attempt to ‘bring that child into the position in which she would have been had the act causing her genetic disorder not taken place, her genetic identity would be different, so the plaintiff would not exist.’ | Undefined | C3 |
| Park, H.G. & Carmel, J.B. (2016) [415] | 1. USA | Medicine (4) | ‘To achieve circuit specific manipulation, genetic tools have been applied using two general approaches. The first is to use the genetic identity of a neuron to drive expression of a gene that can alter its function. This identity can be a transcription factor unique to a circuit; often, transcription factors instruct circuit formation in the first place and some of them retain circuit-specificity in adulthood.’  ‘Genetic identity. Definition: Use of a promoter region of an endogenous gene that defines a neuronal subtype to drive an exogenous gene’ | Defined | F1 |
| Park, Y. et al. (2016) [416] | 5. Korea | Medicine (4) | ‘GVHD remains one of the significant adverse events after HSCT. There have been many studies on the genetic polymorphisms associated with the risk of developing GVHD. However, proxy markers for the identification of genetic identity between HSC donor and recipient pairs and for prediction of the probability developing GVHD have not been well-assessed. The C4 gene belongs to the MHC class III (gamma block of MHC), which is located between the MHC class I and II domains, and is a possible marker for identifying the degree of genetic identity between a recipient and a donor.’ | Undefined | A2i |
| Parsi, K. (1999) [417] | 1. USA | Ethics (3) | ‘He explains for example that: *In a minimalist view, the nonviable fetus is little more than a form of the pregnant woman’s bodily tissue: it is part of the woman without separate identity or status. This image of the fetus as tissue de-emphasizes the importance of the fetus’s separate genetic identity and is most tenable in the early gestational stages .... Fetal remains are most commonly discarded in the same manner as other by-products of surgery .... With no moral status, they are simply thrown away.’* | Undefined | C2 |
| Penchaszadeh (2015) [418] | 6. Argentina | Genetics (4) | ‘Genetics should never again be used to justify abuses such as racism, stigmatization … and should promote actions that benefit humankind while avoiding the trap of genetic reductionism, acknowledging that genetic identity is only one dimension of personal identity.’ | Undefined | D1  B1  B5 |
| Penchaszadeh, V.B. & Schuler-Faccini, L. (2014) [419] | 6. Argentina | Health sciences (4) | ‘Identified children and young adults reacted to the revelation of their true genetic identity in many different ways.’  ‘The search, localization, identification and restoration of the true genetic identity of the children of the disappeared is based on the notion that forced disappearance and suppression of identity are crimes against humanity (United Nations, 2007) and that the state has a legal responsibility for the actions of previous governments and is subject to the international law of human rights.’  ‘At the same time, one should avoid the trap of genetic reductionism and acknowledge that genetic identity is only one dimension of personal identity, which is a complex phenomenon resulting from many factors, including those that are genetic, social, emotional, educational and political in nature.’ | Undefined | D1  A1  B1  B5 |
| Pereira, A.L.D. (2011) [420] | 3. Portugal | Law (1) | ‘On the other hand, looking inside Mankind, genetics make possible a deeper knowledge of human beings. More than fingerprints, DNA seems like a serial code, unique and unrepeatable. Genetic identity is not only a right (such as concerning paternity investigation or the right to natural instead of selected DNA) but also a duty (as for criminal research purposes).’ | Undefined | A1  D1  C1 |
| Perepechina, I. (2017) [421] | 3. Russia | Forensic medicine (4) | ‘The adoption of the standard of DNA identification procedure will allow both the experts and the court to avoid subjectivity in making the decision with regards to the genetic identity in every particular case under consideration. The potential of the available STR-identification systems permits to choose a solid and reliable standard.’ | Undefined | D1 |
| Perepechina, I.O. (2017) [422] | 3. Russia | Forensic medicine (4) | ‘Apart from assessing the objective statistical value for DNA identification reliability, choosing the criterion to establish the genetic identity inevitably invokes the subjective factor. The crucial point of decision-making regarding the identity threshold is unavoidable given any degree the DNA identification reliability.’ | Undefined | D1 |
| Pergament, D (1999) [423] | 1. USA | Law (1) | ‘Specifically, hair has become a nonliving biologic material that, unlike other regenerative tissue or cellular structure, can be used to reveal genetic identity with minimum physical intrusion. Unfortunately, judicial decisions and administrative regulations offer individuals only limited protection from state or institutional intrusion into the information revealed by genetic hair analysis.’ | Undefined | D1 |
| Perlin, M.W. et al. (2009) [424] | 1. USA | Mathematics (5) | ‘There can be conceptual utility in forming and preserving the genotype Q and its probability function q(x). The genotype is a natural representation of genetic identity since it corresponds directly to an individual’s DNA type. Also, its probability distribution captures our knowledge (and uncertainty) about unknown allele pair values. Some people find it helpful to visualize genotype value combinations (e.g. DNA mixtures) and compare these patterns with the observed data.’ | Undefined | E1  A3ii |
| Perry, A. (2012) [425] | 1. USA | Philosophy (3) | ‘Communities are beginning to form around genetic identity, and while it is exciting when new interventions become live options for clinicians, these options also commonly add to bioethical dilemmas. Some deaf parents want to choose to have deaf children (Shaw, 2008), and parents can now select or screen for particular genes through assisted reproductive techniques.’ ‘If talking about this sort of genetic identity raises difficult questions for ethicists, this is particularly true in cases of behavioral genetics such as autism.’ | Undefined | B2  B1 |
| Peters, P.G (2006) [426] | 1. USA | Law (1) | ‘As John Harris has observed, however, it is just as reasonable to claim that a person’s individual life history begins when the gametes that produced her were created or when those gametes were selected and placed in a petri dish together. (…) In ICSI, a single sperm is preselected for manual injection into a single preselected egg. Thus, the complete genetic identity of the resulting embryo is fixed before fertilization. ICSI procedure illustrates that genetic identity can be determined well before conception. As a result, genetic individuality does not capture exactly what we mean when we talk about a person’s conception.’  ‘While assembly of the embryonic genome fixes the genetic identity of any future children, it does not fix their number. Because each twin or triplet is surely a separate individual, individuality is not fixed before the period for twinning is over.’ | Undefined | C2  C1 |
| Pillay, R. (2010) [427] | 7. South Africa | Law (1) | ‘(…) advances in medical science and technology have had a profound impact on the areas of human reproduction, pregnancy and foetology, transforming understanding of the foetus, as an individual with a separate genetic identity from its mother. This has intensified debate on the crucial question of the status of the human being, at its various stages of development. (…) The inertia of courts seems to result from judicial ambivalence and anxiety, rendering our law increasingly out of touch with medical science and technology in defining and developing the conception of human personhood.’ | Undefined | C2  C1 |
| Pizzulli, F.C (1974) [428] | 1. USA | Law (1) | ‘The discernment of privacy and autonomy values brings into focus concerns about both the cloned individual and society: (a) privacy and autonomy might be severely attenuated in one known by himself or others to have a predetermined genetic identity; and (b) irrespective of personal and/or public knowledge of one’s clonal origins, the technology of cloning might have macro-effects upon society by eroding the concept of individuality which is at the core of our notions of privacy and autonomy.’  ‘(…) a clonant’s genetic identity not only deprives him of a unique genotype but also has a detrimental impact upon his ability to experience a unique ‘social environment’ (i.e., physical and psychological stimuli that interact with his genotype subsequent to conception).’  ‘Psychological ramifications in having a ‘second-hand’ genetic identity are largely contingent upon the clonant and/or the persons in his social environment having knowledge of this.’  ‘The psychological functions of privacy that are furthered by constitutional protection of an individual’s choice to withhold personal information underlie any discussion of the invasion of privacy incurred by disclosure of a clonant’s genetic identity.’  ‘The concept of genetic bondage is grounded upon two basic elements. One element is the deliberateness with which one is infused with a predetermined identity.’ ‘The second element of genetic bondage is the degree of predetermination of one’s genetic identity.’  ‘Eugenic sterilization has been one area in which the courts have approved preventing the creation of children on the grounds that nonexistence would be preferable to existence with a handicapped genetic identity.’ | Undefined | C1  B1  D1  C3 |
| Polanco, K.A. (2005) [429] | 1. USA | Law (1) | ‘CODIS targets these repeat offenders by having their genetic identity permanently included in the database for future comparison with DNA left at crimes scenes. CODIS’s ability to identify recidivist offenders furthers the government’s interest in crime prevention.’ | Undefined | D1 |
| Poole, C.A.C. (2013) [430] | 1. USA | Law (1) | ‘B. Mahendra also argued that the most effective way to battle a disproportionate (and thus seemingly discriminatory) amount of minorities in the NDNAD was to institute a national DNA database and to incorporate national genetic identity cards. Mahendra brushed off ‘slippery slope’ type arguments, saying that to give credence to such arguments is to remain ‘blind to the workings of the modern world.’’ | Undefined | A3i  D1 |
| Poole, J. (2010) [431] | 2. UK | Psychology (2) | ‘Genetic Identity (GI)’ ‘OT replaces the biomedical concept of ‘genetic flaw’ with one of healthy, GI. This represents the innate, hard-wired thinking and/or learning style of the individual. OT, unlike current, major theories, does not therefore pathologise or stigmatise the individual on the basis of this difference. It draws into the framework much empirically based and successful educational practice such as that developed from Steinberg’s (1997) multiple intelligences which is important as, even given the emphasis on multisensory teaching in dyslexia, this work has remained outside of the major theories. This is despite established work by Stanovich (1986) on individual differences and a potential ‘Mathew Effect’. The GI/innate thinking style of the child must however be taken into account when teaching literacy because it is this underlying brain wiring upon which literacy is to be mapped, bringing with it specific needs for their development and education.’ | Undefined | B2 |
| Posner, E.A. & Posner, R.A. (1999) [432] | 1. USA | Law (1) | ‘Setting to one side biological uncertainties that we assume will eventually be dispelled, the clone will be a perfectly normal human being, as normal as an identical twin. But the vertical relation of genetic identity has different implications from the horizontal relation. Take the case in which a married couple decides to have two clones, one of each spouse, rather than producing children sexually. If the clones then clone themselves, the original husband and wife will have the same genetic relationship to their grandchildren as to their children, while their children will have no genetic relationship to each other and also their grandchildren no genetic relationship to each other.’ | Undefined | A2i  C3 |
| Prascina, E. (2016) [433] | 3. Italy | Law (1) | ‘Although the principal objective of the legislation continues to be that of offering couples a solution to reproductive disorders, the prohibition on the donation of gametes ensures that each child can be certain about his or her biological origins (right to genetic identity) and excludes the psychological risk that could result from differences between the status of his or her parents and a relationship with a parent not based on a blood relationship.’ | Undefined | A1 |
| Qadeer, I. & Arathi, P.M. (2016) [434] | 5. India | Social Development (2) | ‘The article argues that in contemporary India, the sections where surrogates come from see surrogacy in different ways, and that has to be the starting point for analysis but not the end. Indian lawmakers take advantage of this confusion about the nature of surrogacy. It buries the non-exploitative potential of procreation under the ‘compensation for agreeing’ to be a surrogate, denies her rights and undermines the value of gestational motherhood as compared to genetic identity—thus, killing several birds with one stone.’ | Undefined | A1 |
| Ram, N. (2009) [435] | 1. USA | Law (1) | ‘Second, incorporating fair utilization in an informational property system begins to grapple with the tensions that shared genetic identity present for traditional notions of private property. As suggested above, a commons-centered approach to human genetic information may in many ways appear attractive because even non-related individuals differ at the genetic level only by tenths of a percent. Fair utilization would open access to material for purposes of researching those portions of DNA that are shared among all humans – essentially creating a limited genetics common.’ | Undefined | D3  A2ii |
| Rankin, J.C. (1988) [436] | 1. USA | Ethics (3) | ‘At the moment of conception the sperm fertilizes the egg, and a dramatic  and instantaneous change occurs. These two complementary haploid cells form a diploid cell, which is otherwise known biologically as a whole body. This diploid life has a genetic uniqueness, completeness and wholeness as a one-celled zygote. Such a zygote formed at conception has all the genetic identity and programming to mature into an embryo, a fetus, a baby, a child, a teenager, an adult.’ | Undefined | C2 |
| Rao, R. (2002) [437] | 1. USA | Law (1) | ‘Cloning consequently results in the replication of an existing genome; it does not involve the random recombination of genes, which in sexual reproduction results in a child with a new and unique genetic identity.’  ‘Second, as a consequence of the first feature, cloning also provides the awesome power to engage in a form of genetic selection, to choose the child’s entire genome, and to predetermine its genetic identity. Instead of risking the randomness of the genetic lottery to create a child with a completely unpredictable mix of genes, prospective parents can use cloning to simply duplicate an existing person, a person with a known genetic identity.’ | Undefined | C3  C1  E6 |
| Rao, R. (2006) [438] | 1. USA | Law (1) | ‘(…) advances in genetics and technology threaten privacy by making it possible to perform genetic tests to reveal each person’s entire genetic identity easily and precisely.’ ‘In one way, we are all genetically sui generis, so that there is never any real basis for drawing a comparison. Yet discrimination based upon genetic identity faces both the problem of no precise basis for comparison and too many possible or plausible bases for comparison. While we are each genetically distinct and unique individuals, we also possess multitudes of common genes for a vast number of traits.’ ‘Further, technological developments and social practices may effectively eviscerate a right of genetic privacy that rests upon reasonable expectations, for it would be unreasonable for individuals to expect their genetic identities to remain private in the face of pervasive genetic testing.’ | Undefined | D1 |
| Rasmuson, M. (1993) [439] | 2. Sweden | Genetics (4) | ‘Genetic identity, which may be important for kin recognition, is the fraction of the genome that is identical by descent. It is, except for the parent—offspring relation, governed by probability and its variance depends on the number of segregating units during meiosis. Using the recombination index as an approximation of this number the variance for genetic identity has been estimated for different kinds of kinship.’ | Defined | A3ii |
| Raspberry, K. & Skinner, D. (2007) [440] | 6. Argentina | Anthropology (2) | ‘Our findings reveal that for many engaged in clinical genetics, a genetic identity is one part of the lived experience of the genetic body. We emphasize, however, that this notion of the genetic body does not imply an essentialized, static sense of being, but rather is a particular way of experiencing one’s health and identity as predefined and familial.’  ‘In several respects, these parents’ understandings of genes and the body can be interpreted in terms of genetic determinism and essentialism, yet it would be inaccurate to characterize their beliefs exclusively in this way. Coexisting alongside their understandings of health as predetermined and their incorporations of a genetic identity as part of the self are beliefs in their child’s potential for developmental progress and achievement, and their practices that confirm this optimism, such as searching for educational, therapeutic, and medical services that they believe can help their child.’  ‘While incorporating some sense of the child’s genetic composition into their understanding of the child, parents do so within a holistic context. Although an important part of who or she is, parents see their child as more than just the disorder. Genetic identity is one of many identities, one of many ways of defining and understanding oneself and progeny.’ | Defined | B1  B5  B2 |
| Rath, N. & Olson, M.F. (2016) [441] | 2. UK | Molecular cell biology (4) | ‘Pancreatic ductal adenocarcinoma (PDAC) is characterized by an extensive stromal component that hinders treatment. A new study shows how the genetic identity of pancreatic tumors might influence the physical properties of the associated stroma to promote tumor progression.’ | Undefined | F1 |
| Ravitsky, V. (2010) [442] | 1. Canada | Bioethics (3) | ‘The need to understand ‘where you came from’ thus creates a need to have access to information that relates to the donor’s narrative identity, not just biological or genetic identity. Donor-conceived individuals speak of their need to  hear stories, to know biographical facts, and to see pictures of the donor.’ | Undefined | A1 |
| Reilly, C. (1994) [443] | 1. USA | Law (1) | ‘The oocytes are then harvested by a minor surgical technique and placed in a laboratory dish containing sperm. Fertilization, or the penetration of an oocyte by a single sperm, may take up to twenty-four hours. The resulting single-cell organism, or zygote, now contains the pairing of chromosomes necessary to create a unique genetic identity. The zygote begins to subdivide into a multi-cell, yet undifferentiated, organism labeled a ‘preembryo. ‘ The preembryos are placed into the uterus in the four-to-eight-cell stage, usually two to three days after the harvest of oocytes.’ | Undefined | C2 |
| Resnik, D.B. (2001) [444] | 1. USA | Bioethics (3) | ‘But even though the genome exhibits a great deal of influence over human physiology and behavior, equating genetic identity with personal identity amounts to a I and dangerous form of genetic reductionism. To address this briefly, I would say there are two basic approaches that have received some acceptance. The most popular approach, the psychological approach, equates the person with his or her various psychosocial and behavioral traits, such as personality, intelligence, and memory. The physiological approach, on the other hand, focuses on physical traits that can be used by other people to identify the person, such as fingerprints, height, and facial characteristics. Modern science tells us that human physiology, behavior, and psychology result from both nature (the genes) and nurture (development and the environment); the genome alone does not determine physiology, psychology, or behavior.’  ‘In his essay, Brody also considers whether DNA patents used to alter the human genome would violate human dignity. He notes that the European Commission has proposed that processes or objects used to modify genetic identity for non-therapeutic purposes be deemed to violate human dignity. The concern here is with DNA patents that enable people to genetically engineer human beings for eugenics purposes. While DNA patents for inventions designed to alter the human germ-line create a wide range of moral problems, they only threaten human dignity as long as they do not pertain to a whole genome; if this is the case, they do not violate human dignity in and of themselves.’ | Undefined | B5  B1  B6  C3  D3  E6 |
| Resnik, D.B. (2010) [445] | 1. USA | Bioethics (3) | Moreover, the idea of linking personal identity to genetic identity is highly problematic, since genes do not determine behavior, neurophysiology, or other traits necessary for personhood. Identical twins have the same genome, but they are different people, with different personalities. Koepsell seems to appreciate this important point, but he still makes the philosophical mistake of equating genes and persons. | Undefined | B1  B5  E6 |
| Revel, M. (2003) [446] | 5. Israel | Bioethics (3) | ‘These are all illusions of genetic identity, based on extreme genetic determinism, and indeed should be a priori condemned and never permitted. First, because it is not true that two individuals with the same genetic make-up (twins) would have identical lives and behavior (or even beauty, which is in great part subjective and subject to a desire to be beautiful and to a discipline to achieve this goal, not to speak about intelligence which as shown by the IQ debate is in great part due to environment and education). The human soul cannot be cloned.’ | Undefined | B5  B6  B3  C3  A2i |
| Ricci, U. et al. (2004) [447] | 3. Italy | Genetics (4) | ‘The MZ twins are considered genetically identical and the reduced phenotypic, genetic or chromosomal concordance represents a very important challenge for geneticists. (…) We utilized a protocol based on the CODIS polymorphic markers battery, by using an infrared LI-COR 4200 instrument (LICOR, Nebraska, USA), and we associated the statistical analysis method with formulae derived by the DNA-View’s Kinship Module to better evaluate the genetic identity in a group of MZ twins.’ | Undefined | A2i |
| Rice, A.E. (2006) [448] | 1. USA | Law (1) | ‘Silvers and Stein, on the other hand, advocate what they call ‘an equalityparadigm,’ which would entail a broader prohibition of discrimination on the basis of ‘genetic identity.’ This model is subject to the criticism of overinclusiveness discussed above. Nonetheless, it provides more complete coverage for asymptomatic and presymptomatic individuals than does Greely’s model because coverage does not depend on the source of genetic information.’ | Undefined | D1 |
| Richards, V. (1969) [449] | 1. USA | Medicine (4) | ‘The fourth source of kidneys for transplantation is the living donor. Transplantation of kidneys between identical twins was accomplished in the late 1950’s after studies demonstrated that genetic identity between the donor and recipient invariably permitted the successful transplantation of a kidney.’ | Undefined | A2i |
| Riley, M.F. & Merrill, R.A. (2004) [450] | 1. USA | Law (1) | ‘The moral issues addressed by HERP are at the crux of the controversy surrounding embryo research. HERP examined two competing frameworks for assessing the moral status of the embryo. The first holds that there is a single criterion of moral personhood. Those who meet that criterion are entitled to full moral respect as humans; those who do not have a lesser status. According to the Panel’s account of single criterion views, humanness occurs at a defined point. For some, humanness is determined by a distinctive human genetic identity. For some others, an embryo is human at the moment of conception. Or, humanness may be dependent on reaching a point of human potential; a point reached at or near conception-either at the time of syngamy, when the chromosomes of the male and female gametes join, or at the four to eight cell stage when gene expression begins. Others fix upon a later point in development, sentience or the beginning of brain activity.’ | Undefined | C2  B7 |
| Rimmer, M. (2007) [451] | 4. Australia | Law (1) | ‘The Project describes itself in the following terms:  Our genes allow us to chart the ancient human migrations from Africa across the continents. Through one path, we can see living evidence of an ancient African trek, through India, to populate even isolated Australia. But to fully complete the picture we must greatly expand the pool of genetic samples available from around the world. Time is short. In a shrinking world, mixing populations are scrambling genetic signals. The key to this puzzle is acquiring genetic samples from the world’s remaining indigenous and traditional peoples whose ethnic and genetic identities are isolated. But such distinct peoples, languages, and cultures are quickly vanishing into a 21^st^ century global melting pot.’ | Undefined | A3i |
| Robert, E. (2017) [452] | 1. USA | Religious studies, philosophy (3) | ‘The legacy of race and ethnic nationalism bleeds into contemporary literature around the genetics of Jews. That is, despite the use of sophisticated technological and scientific systems in accounts of genetics, this discourse remains conceptually bound to essentialist notions of race and nationalism. Baker deftly undermines the veneer of objectivity around scientific attempts to secure identity. Not only are these attempts inevitably driven by present needs and desires, readily limiting who counts as what in order to justify assorted agendas, but even disinterested sounding terms like ‘genetic identity’ presuppose specific notions of ancestry and racial purity. It comes as no surprise, then, that studies about the genetics of various populations are often used to justify and contest territorial claims. Indeed, resonating with her discussion about the problems gender poses to ethnicity as a marker of ancient Jewishness, Baker draws attention to the significant presence of European women (at least 80%), incorporated into the community through conversion, marriage, and motherhood, in Ashkenazic Jewish genetics (109). | Undefined | A3i |
| Roberts, D.E. (1995) [453] | 1. USA | Law (1) | ‘We often perceive a special relationship created by a shared genetic identity. When a new baby enters a family, one of the first responses is to figure out whom she resembles. Most parents probably feel great satisfaction in having children who ‘take after’ them. Bringing into the world children who bear their likenesses gives many people both the joy of creating another life and the comfort of achieving a form of immortality passed down through the generations.’ | Undefined | A2ii |
| Roberts, J.L. (2011) [454] | 1. USA | Law (1) | ‘For example, prior to GINA, characteristics receiving antidis- crimination protection constituted socially recognized groups with associated identities and the potential to become objects of stigma. By contrast, genetic information-at least at present-does not comprise a lived social category in the same way as race, sex, or disability. While much has been written on the subjects of racial identity, gender identity, and disability identity, genetic identity still remains largely a mystery. Because genetic science is still in its infancy, genetic information is too young as a social category to have developed a recognized identity group or stigma based on group membership. Furthermore, acquiring genetic information requires testing, whereas other groups tend to rely on social and cultural signals and morphological traits as indicators of group membership. Even if people were actively seeking genetic testing, the parameters of our genetic identities would still be unclear by virtue of the fact that science is currently unable to decipher the exact meaning of much of that information. At present, we can only speculate about the type and number of identity groups that might arise with respect to genetic information or who will be considered a member.’ | Undefined | D1  B1 |
| Roberts, J.L. (2015) [455] | 1. USA | Law (1) | ‘Conversely, Silvers and Stein argued in favor of antidiscrimination protection on the basis of genetic identity as an alternative to the privacy paradigm.’  ‘(…) as genetic technology continues to advance, and as people begin to develop senses of genetic identity, the need for promoting genetic diversity could very well soon follow. (…) Thus, privacy protections to further antidiscrimination goals should also permit exceptions when the employer wishes to obtain the relevant information to promote diversity.’ | Undefined | D1 |
| Roberts, J.L (2016) [456] | 1. USA | Law (1) | ‘Research indicates that individuals who take genetic tests will incorporate the results of their tests into their self-concept, creating a sense of genetic identity. In fact, some individuals report strong negative affect regarding their level of genetic risk. People with heightened genetic risk have described themselves as ‘flawed,’ ‘dirty,’ and vulnerable. Those individuals-much like women, members of racial minorities, or older people-might be reminded of their perceived inferiority and therefore perform worse when an employer inquires into their genetic profiles. Furthermore, even if the individual person does not see her status as negative, she might be aware of her employer’s perception that she may constitute a liability, which could likewise have a harmful effect. Genetic information could then also be a basis for stereotype threat, albeit an untraditional one.’ | Undefined | B2  D1 |
| Roberts, M.A. (1996) [457] | 1. USA | Philosophy & religion (3) | ‘In particular, I argue that cloning harms its offspring by violating the interest we all share in retaining, where we reasonably can, control over our own genetic identity.’  ‘Thus, Robertson suggests that the life of the offspring, even if diminished in certain respects as a consequence of sharing a complete genetic identity with one or more individuals who may or may not be of same age and may or may not be raised in the same family, is a life worth living or, in his words, not a life that ‘never should have occurred.’’ | Undefined | C1  C3  A2ii |
| Roberts, M.A. (1999) [458] | 1. USA | Philosophy & Religion (3) | ‘But for this very reason, if anything, the consent requirement may well be more important in the case of cloning. We eschew the loss of control over our genetic identity. We do not want a situation in which we would exist as one of perhaps many genetically identical individuals rather than as one of one being thrust upon us without our consent.’ | Undefined | C1  C3 |
| Roberts, S.C. et al. (2005) [459] | 2. UK | Medicine (4) | ‘The idea that body odor reveals information about both genetic identity and genetic similarity is most readily tested by examining odor in twin pairs. ‘ | Undefined | A2i |
| Robertson, J.A. (1986) [460] | 1. USA | Law (1) | ‘Thirdly, the preimplantation embryo is substantially different in physiology and development from an implanted embryo and later fetus. Development after fertilization involves passage through a succession of developmental stages, all of which contribute in various ways to the emergence of a fully formed newborn infant. Fertilization marks genetic identity. Implantation, however, marks the development of biologic individuality, as well as the first emergence of the rudiments of the nervous system. The embryonic disc, axis and primitive streak, which begin to emerge at or after implantation, are the precursors of embryonic and fetal structures of feeling, suffering and sentience. Until they emerge there is no possibility of feeling or experience of any sort. (…) Indeed, until the formation of the embryonic axis, developmental individuality is not even established.’ | Undefined | C2 |
| Robertson, J.A. (1987) [461] | 1. USA | Law (1) | ‘Personhood is an evaluative term. Ordinarily the term connotes a certain level of cognitive and relational ability. (…) It is difficult to argue that fertilized eggs, embryos and early fetuses have that capacity, when their neurological capacity is so limited. Neither genetic identity nor the potential to develop great cognitive ability is equivalent to already having attained the requisite stage of development.’ | Undefined | C2 |
| Robertson, J.A. (1988) [462] | 1. USA | Law (1) | ‘Differing moral-religious views of the status of the embryo explain much of the ethical, legal and policy debate about embryo technology. A fertilized egg or early embryo has genetic identity and the potential to initiate a pregnancy, but it is a collection of cells that has not yet differentiated into the placental layer that must emerge before the embryo proper is formed. Only after implantation at 10-14 days post-insemination will the primitive streak, the rudimentary form out of which the neuromuscular system develops, appear.’ | Undefined | B3  C2 |
| Robertson, J.A. (1988) [463] | 1. USA | Law (1) | ‘Yet one might reasonably conclude that the offspring’s interest in personal and genetic identity outweighs the privacy interests of donors and recipients. Whether restrictions on donor anonymity would dry up the donor pool or merely alter its characteristics must await further experience. The needs of offspring to know about their origin may justly take priority over donor and recipient wishes for privacy, even if it alters the pool of egg donors and prevents the birth of some children who would otherwise have been born.’ | Undefined | A1  D1 |
| Robertson, J.A. (1998) [464] | 1. USA | Law (1) | ‘Partial egg donation does even less violence to notions of female kinship and genetic identity than does full egg donation. Although the donor provides an entire egg in oocyte transfers, only the cytoplasm of the donor egg is used in the recipient’s reproduction. The donated cytoplasm enables the recipient to have healthy offspring, but the entire nuclear DNA-over 99 percent of total DNA—comes from the recipient, who then gestates and rears. If genetic identity is tied to nuclear DNA, donation of mtDNA in the cytoplasm alone would not make the oocyte donor the genetic mother of the resulting child, as is the case for the donor in full egg donation. Thus no significant question of rearing rights or duties or genetic maternity should arise. Although the egg donor has passed her mtDNA on to another generation, the donor herself appears not to have genetically reproduced, as we commonly understand that concept, and would have no plausible claim to override her predonation agreement to relinquish all rearing rights and duties in children resulting from the donation to the recipient and her partner.’ | Undefined | E2  E3  A1 |
| Robertson, J. A. (1998) [465] | 1. USA | Law (1) | ‘Significant kinship issues arise only when an unrelated third party, one of the partners, or a parent of one of the partners provides the DNA. The first scenario is typical of embryo donation-an accepted practice. The second is more novel, but responsible parents probably could deal with the special challenges that intergenerational genetic identity might pose. The most troublesome case-cloning and rearing one’s own genetic parent-is likely to be both so rare and so divorced from the usual reproductive context that it might not be an exercise of procreative liberty at all.’ | Undefined | A2i  C3 |
| Rockandel, K. (2013) [466] | 1. USA | Law (1) | ‘Your identity has value, and sometimes that value belongs to you. States may protect, for example, the commercial value of your name, voice, signature, photograph, and likeness. A handful of states have also considered protecting the value of personal DNA. Extending the same protections that we enjoy in our physical characteristics to our genetic makeup would appear to be a logical step because our genetic material is ‘uniquely linked to our identity as a person.’ Indeed, ‘[e]very feature of our physical being is coded for by DNA, and many scientists would argue that virtually every aspect of humanity is similarly encoded.’ In other words, the physical characteristics to which we claim a property right often manifest our underlying genetic identity.’ | Undefined | D1  D3 |
| Rojas, H. (2004) [467] | 6. Chile | Law (1) | ‘Article 1 of the Bill points out that an individual’s life, physical and psychological integrity, as well as their dignity and genetic identity should be protected, especially in cases like scientific research, including its applications.’  ‘Genetic identity can only be understood as a key element of our privacy  and dignity as human beings. If we do not do so and prevent discriminatory actions committed by employers, insurance companies, and others, the social effects could be disastrous. The Chilean legal response to these issues has been slow.’ | Undefined | D1 |
| Rolston III, H. (2006) [468] | 1. USA | Philosophy (3) | ‘Ask the question in terms of genetic identity. Where is a gene? The cybernetic answer differs from the molecular answer. Genome scientists, as we noted, report that they have identified the same gene in organisms from yeast to humans or that a gene has been conserved since Cambrian times. But if such similar DNA sequences have been repeatedly located, what constitutes their common identity? The ‘‘where’’ question transforms from one of physical location to informational location.’  ‘Any particular gene-token is quite mortal. All that can survive in any long term sense is the gene-type. A few genetic tokens are passed intergenerationally, one copy for each ancestor-descendant crossing. As the zygote develops, that one gene token thereafter makes myriads of other gene tokens. If there is any genetic identity preserved, it is an identity of replicas.’ | Undefined | A2ii  A3i |
| Ronce, O. et al. (2000) [469] | 2. France | Evolutionary sciences (4) | ‘Consider, for instance, a situation where there are exactly four young adults and one old adult per sub-population. The probability of identity between two juveniles born to an old adult in the same sub-population is then necessarily one. The average genetic identity between two such offspring is well below this value when the local age structure fluctuates.’ | Undefined | A3ii |
| Rosner, F. (1983) [470] | 1. USA | Medicine (4) | ‘Jewish sources do not discuss testicular transplants, but similar principles  would probably apply. (…) There may be a distinction between the transplantation of a sex organ and the transplantation of any other organ. The rule that a transplanted organ becomes an integral part of the body of the recipient may not apply to the implantation of a zygote or embryo into a host mother’s womb or to the transplantation of ovary or testicle. Sperm and egg retain the full genetic identity of their donors. The process of fertilization, whether in vivo or in vitro, does not alter the genetic paternity or maternity of the eventual fetus. An ‘incubator mother’ may not have maternal status.’ | Undefined | A1 |
| Rosner, F. (1992) [471] | 1. USA | Medicine (4) | ‘Jewish sources do not discuss testicular transplants, but similar principles would probably apply. (…) The rule that a transplanted organ becomes an integral part of the body of the recipient may not apply to the implantation of a zygote or embryo into a host mother’s womb or to the transplantation of ovary or testicle. Sperm and egg retain the full genetic identity of their donors. The process of fertilization whether in vivo or in vitro does not alter the genetic paternity or maternity of the eventual fetus. An ‘incubator mother’ may not have maternal status.’ | Undefined | A1 |
| Rothenberg, K.H. (1997) [472] | 1. USA | Law (1) | ‘Genetic accountability and genetic identity are now expanding beyond prenatal testing. Jewish women may perceive a ‘social obligation to do anything they [can] to advance’’ research on BRCA1 testing. Accordingly, at least one researcher has warned that ‘those obtaining consent for Jewish women [for BRCA1 testing] should be aware of the ‘slippery slope’ from perceived social responsibility to coercion.’ Furthermore, Jewish women feel particularly responsible for seeking information about genetic predisposition to breast cancer, in part for the ‘sake of one’s children.’ As noted earlier, much of the attention in research, the commercial market place, and the media over the last year has been on the ‘Jewish genes’ for breast cancer. Newspaper headlines sum it all up: ‘Doctors Launch New Jewish Cancer Test’ and ‘Doc Wants New Study of Jewish Cancer Gene.’’ For Jews, a genetic identity to familial cancer is being legitimized by our drive for the genetic ‘quick fix.’’ | Undefined | B2 |
| Rothenberg, K.H. (1999) [473] | 1. USA | Law (1) | ‘While these scenarios may be fascinating, in the end they do not enrich our moral understanding of cloning for two reasons. First, they grow out of a reductionist ‘genetic myopia.’ These scenarios assume that the genetic identity of a person constitutes the complete make up of that person, and that any person created by cloning would be identical in every respect to the adult that had been cloned. Clearly, we know this is not true. Genetically identical twins are distinct persons with individual personalities and individual legal rights. The same would be true of any person cloned. | Undefined | B5  C3  A2i  B1  B6 |
| Rothstein, M.A. & Carnahan, S. (2001) [474] | 1. USA | Law (1) | ‘Courts typically interpret the Fourth Amendment to require a warrant where bodily intrusion is involved. One court held a warrant was required to swab the inside of a suspect’s mouth to obtain a saliva sample. The court reasoned that one’s saliva contains a significant amount of private genetic identity information, and saliva is not an item generally exposed to the public, even though the act of expectorating is somewhat commonplace.’ | Undefined | D1 |
| Roze, D. & Rousset, F. (2003) [475] | 2. France | Evolutionary sciences (4) | ‘We propose here a simple method to construct diffusion approximations in structured populations; it relies on general expressions for the expectation and variance in allele frequency change over one generation, in terms of partial derivatives of a ‘fitness function’ and probabilities of genetic identity evaluated in a neutral model.’ | Undefined | A3ii |
| Ruru, J. (2005) [476] | 4. New Zealand | Law (1) | ‘The former simply involves conscious individual choice(s) to allow another couple to regard the child as their own. For example, in the assisted human reproduction scenario, several competing rights and responsibilities arise between the:   - child’s right to know his or her genetic identity;’ | Undefined | A1 |
| Sabatello, M. & Appelbaum, P.S. (2016) [477] | 1. USA | Bioethics (3) | ‘As sociological and anthropological scholars have observed, since the inception of the Human Genome Project, both medical practice and popular perception are increasingly ‘geneticized.’ Advances in genetic testing have led to the much-debated notion of ‘genetic identity’ as an individual and collective attribute, and to the emergence of voluntary networks of individuals with genetic conditions (and their families) as key partners in scientific endeavors and policy-making. Although these patient- and family-based associations originated to provide members with education and support, they began to engage with lobbying for additional funding for research about their conditions and with efforts to find treatments and cures. The combined effect of these processes transformed patients’ empowerment and collective mobilization into a ‘genetic citizenship’ that encompasses rights and obligations. Although informed consent and free choice remain the cornerstones of the decision-making process, individuals increasingly have been expected to engage in testing and adopt self-surveillance methods if they are at-risk for a genetic condition.’ | Undefined | B2  D1  B5 |
| Sappideen, C. (1983) [478] | 1. Australia | Law (1) | ‘Who then is the mother — the genetic mother who supplied ova used in the in vitro fertillisation procedure or the surrogate as gestational mother? The legislative and judicial response to the question will depend on an evaluation of the significance of genetic identity as part of the process of motherhood. A trend reducing the significance of genetic identity has been discerned in A.I.D. decisions in the United States and in child placement cases. It has also been pointed out that genetic identity does not guarantee that parents will act responsibly, nor has genetic identity been regarded as critical in all societies. This is also reflected in a movement towards genetic responsibility where there is a substantial risk of severe genetic defect; advocates argue that there is no inviolate right for all to reproduce when the resulting child has serious genetic defects with high cost to the community. In an era when the ‘nature-nurture’ debate (whether intelligence is genetically ordained or nurtured by environment) continues to rage, the importance of the uterine environment to the subsequent development of the child is largely unknown.’  ‘Even if the child knows his genetic identity he might still have a sense of abandonment by the surrogate and this is in turn may cause psychological difficulties. The child might feel a need to seek out the surrogate in the same way adopted children seek out biological parents.’ | Undefined | A1  B1  B2  B6  D1 |
| Sass, H.M. (1996) [479] | 2. Germany | Philosophy, bioetics (3) | ‘Years ago, the European Community appropriately included the right to a non-manipulated genetic identity in the list of civil rights. That position should be universally supported by all nations and cultures. But if and when reliable methods become available through genetic modification to ‘heal’ some of the severest forms of human genetic disorders in germ-line cells, then the benefits and risks, medical and moral, will have to be compared to the benefits and risks of indirect ‘prevention’ through pre-implantation diagnosis or abortion, or to the benefits and risks of knowingly giving birth to severely handicapped offspring without any further moral ado.’ | Undefined | C1  E5  C3 |
| Saunders, L.R. et al. (2000) [480] | 1. USA | Microbiology & immunology (4) | ‘The NFAR gene (nuclear factor associated with dsRNA) encodes a putative transcription-associated factor that we have shown is a substrate for the interferon-inducible, dsRNA-dependent protein kinase, PKR. However, our protein expression analysis has revealed that NFAR exists as two major protein species of 90 kDa (NFAR-1) and 110 kDa (NFAR-2) in the cell. To resolve the genetic identity of NFAR-1 and -2, we carried out sequence analysis of genomic and cDNA NFAR clones and determined that the coding region of this gene spans 16.2 kb and comprises 21 exons.’ | Undefined | F1 |
| Savell, K. (2006) [481] | 4. Australia | Law (1) | ‘Lord Hope adverted to the practices of reproductive technologies, which serve ‘to remind us that an embryo is in reality a separate organism from the mother from the moment of conception.’ Lord Mustill also mphasized the importance of genetic identity: There was, of course, an intimate bond between the foetus and the mother, created by the total dependence of the foetus on the protective physical environment furnished by the mother, and on the supply of the mother through the physical linkage between them of the nutrients. Oxygen and other substances essential to foetal life and development. The emotional bond between the mother and her unborn child was also of a very special kind. But the relationship was one of bond not identity. The mother and the foetus were two distinct organisms living symbiotically, not a single organism with two aspects.’ | Undefined | C2 |
| Sawyer, S. & Felsenstein, J. (1981) [482] | 1. USA | Mathematics (5) | ‘A probability model of a population undergoing migration, mutation, and mating in a geographic continuum R is constructed, and an integrodifferential equation is derived for the probability of genetic identity.’ | Undefined | A3ii |
| Scarnecchia, D.B. (2013) [483] | 1. USA | Theology/ bioethics (3) | ‘The best case scenario of homologous embryo transfer occurs when a married couple repents of their sin of IVF and makes amends by attempting to implant and carry to term in her womb their frozen embryos. The law should allow them to do so. Her husband would prime her immune system, so as to enhance the likelihood of a successful implantation, through morally licit acts of marital intercourse. Their embryo once it implants, will further their one-flesh union through normal fetal mircochimerism. The maternal-fetal cells that pass from her into her child during pregnancy and that may continue to pass into it, should she breastfeed, will not complicate her child’s genetic identity. The frozen embryo that enters its genetic mother’s womb under these best of circumstances has its right to genetic, gestational, and social parentage vindicated. This child does not trespass upon the dignity of marriage, a child who in its flesh furthers the one-flesh union of its fallen but repentant procreators.’ | Undefined | C2  B6 |
| Scherr, A.E. (2013) [484] | 1. USA | Law (1) | ‘The construction of identity is a complex, layered phenomenon that resists essentialist simplicity. Anthropologists have long debated identity essentialism and have brought that discussion to the world of genetics. Since the earliest days of the new genetic research, biologists have struggled with the concepts of genetic determinism and essentialism. Richard Lewontin and others have written at length and compellingly about the dangers of drawing too much meaning from one’s genes to the exclusion of other fundamental factors at play in genetic expression like environment and the host organism. GATTACA, a recent movie, captures one dystopic version of a society overly obsessed with deterministic essentialism of genetic identity.’ | Undefined | B5  B6 |
| Schiff, A.R. (1994) [485] | 1. USA | Law (1) | ‘However, these similarities should not obscure one critically important distinction between sperm donation and blood donation: while blood is life-sustaining, sperm is life-creating. Blood supports fife, but sperm is used to create a new and unique genetic identity, a human being. Given the life-generating quality of sperm donation, how should society view this activity?’ | Undefined | C2 |
| Schiff, A.R. (1995) [486] | 1. USA | Law (1) | ‘While powerful arguments exist in favor of recognizing gestation as the determinant of legal motherhood in egg donation, there is also a strong case for viewing genetics as the paramount criterion. The genetic identity of an individual may have a profound effect upon a person’s physical characteristics, predisposition to certain diseases, and, according to some, even upon temperament, intelligence, and behavior. Although the debate over the relative contributions made by ‘nature’ and ‘nurture’ continues, the relevance of the genetic component cannot be discounted. As one anthropologist has commented: [T]wo individuals who differ genetically in metabolic characteristics controlling behavioral tendencies will, if raised in identical environments with identical resources and training, grow up to act and think and feel differently... There is in each of us a residue of characteristics of heart and mind that we brought with us when we entered the womb, a mere few days from conception. The denial of this, as liberal as it usually sounds, is really a denial of individuality in the most fundamental sense, and is every bit as dangerous as the most rigid forms of genetic determinism.’ | Undefined | A1  B1  B6  B2  B5 |
| Schiff, A.R. (1997) [487] | 1. USA | Law (1) | ‘Views may vary, however, as to whether the state’s interest in protecting an embryo is greater or less when the embryo is in vitro rather than in vivo. On the one hand, it may be argued that the state’s interest in protecting extracorporeal embryos is relatively weak, because the embryo is at such an early developmental stage. In contrast, an embryo in the uterus embarks upon a developmental process which, if successful, culminates in a live birth. The state’s interest in the growing fetus becomes compelling once the fetus has passed certain developmental milestones. While an extracorporeal embryo does constitute a unique genetic identity, its development is so preliminary that the state’s interest may be, as the court in Davis observed, ‘at best slight.’’ | Undefined | C2 |
| Schroedel, J.R. et al (2000) [488] | 1. USA | Politics and Policy (2) | ‘Fetal rights advocates usually begin the debate with a moral argument about the need to protect unborn life. The humanity of the fetus is axiomatic, but some argue ‘scientifically’ that the fetus is a ‘tiny person’ with the requisite forty-six chromosomes for a unique genetic identity from conception. A range of public policy and legal justifications for state intervention on behalf of the fetus follow. Implicit is the underlying policy goal: to prevent the widespread killing of fetuses (i.e., abortion). | Undefined | C2 |
| Schultz, K. (2012) [489] | 4. Australia | Law (1) | ‘By contrast, ontological objections direct that arguments for protecting future generations founder on the contingency of their identity and makeup. These ontological objections centre on issues of non-identity, and can be illustrated by two primary complaints. First, future generations (as opposed to future individuals) cannot be moral entities as they lack moral agency — no moral agency can be imputed to a generation, as opposed to an individual. Second, even future individuals are not moral entities, on the basis of their contingency- they suffer from Parfit’s non-identity problem, or (applying this) from Gaba and D’Amato’s paradox of future individuals. For Parfit, policy selection brutally affects the identity of future individuals- different individuals will be conceived, and so different genetic identities will result.’ | Undefined | C3  A3i |
| Schumacher II, R.W. (1999) [490] | 1. USA | Law (1) | ‘Although a relatively new analysis, saliva sampling is favorably comparable with the testing of blood and urine. First, the procedure involves an intrusion reaching ‘beyond the physical characteristics exposed to the public and into the security of the person.’’ Second, a saliva sample can, like blood and urine, provide significant amounts of genetic identity information. Over the last decade, courts utilized these factors in asserting that an oral swabbing procedure, like the one suggested by Safir, implicates the Fourth Amendment.’ | Undefined | D1 |
| Schwartz, P.M. & Solove, D.J. (2014) [491] | 1. USA | Law (1) | ‘The Proposed Regulation provides additional examples of the kinds of linkages that tie information, whether directly or indirectly, to a person. The new examples refer to ‘location data,’ ‘online identifier[s],’ and ‘genetic’ identity. The impact of these additional categories is to modernize and expand the sweep of the 1995 Directive.’ | Undefined | D1 |
| Schwartz-Marin, E. & Restrepo, E. (2013) [492] | 2. UK | Anthropology (2) | ‘Biocoloniality recovers how elements of coloniality are constitutive of the scientific making of populations, producing ‘genetic identities’ (especially those linked to existing discourses of race and nation) which are understood as being in need of protection and/or preservation from capitalist expropriation or the unruly circulation of biocapital (Sunder Rajan, 2006). In the present article, we argue that, despite its apparent emancipatory promise, the creation of ‘genetic identities’ and the legal schemes designed to protect them – crafted at the crossroads of science and politics – necessarily reintroduces and reinforces racialised modern dualisms, and with them elements of coloniality.’ | Undefined | A3i  D1 |
| Scola, A. (2011) [493] | 1. USA | Law (1) | ‘Judge Sweet’s trepidations mirror the public’s concerns about the gene-patent debate. The plaintiffs’ legal challenges to the BRCA patents raise a difficult legal and ethical dilemma: should information about an individual’s personal genetic identity be protectable as intellectual property for the purpose of promoting scientific innovation? On the one hand, resolving this thorny question in favor of the plaintiffs could affect the future of biomedical research. Conversely, a resolution favoring the defendants affects both personal healthcare and autonomy.’ | Undefined | D3 |
| Segal, N.L. (1984) [494] | 1. USA | Psychology (2) | ‘Behavioral analyses using nonhuman subjects have demonstrated that social-interactional processes and outcomes may be largely fashioned by the relative genetic identity or nonidentity of the participants. Comparisons of social relationships between monozygotic (MZ) and dizygotic (DZ) cotwins enable a test of this concept at the human level. The cooperative and competitive behaviors of forty-seven IQ-concordant twin pairs between six and eleven years of age were observed during the completion of joint ‘projects’ or tasks. MZ partners provided striking evidence of greater cooperation, relative to DZ partners. These results are discussed in light of social-genetic and kinship-genetic explanations of behavior.’ | Undefined | A2i  A2ii  B1  B6 |
| Segal N.L. (2012) [495] [495] | 1. USA | Psychology (2) | ‘Monozygotic cotwins are ideal organ donors for one another due to their genetic identity.’ | Undefined | A2i |
| Segal, N.L. & Marelich, W.D. (2011) [496] | 1. USA | Psychology (2) | ‘Twin-family designs yield an array of genetically related individuals, conducive to testing evolutionary based hypotheses regarding social closeness. Monozygotic twins’ genetic identity makes them the ‘‘genetic parents’ of their nieces/nephews. However, dizygotic twins retain customary aunt/uncle relationships with their co-twin’s children. A 2007 study found that MZ twin aunts/uncles expressed greater social closeness toward nieces/nephews than DZ twin aunts/uncles, consistent with predictions from inclusive fitness theory.’ | Undefined | A2i  B1  A2ii |
| Segal, N.L. et al (2007) [497] | 1. USA | Psychology (2) | ‘Twins’ marriages to non-twins yield genetically and socially informative kinships. Monozygotic (MZ) twins’ genetic identity makes them ‘genetic parents’ of their nieces/ nephews, and their nieces/nephews their ‘genetic children’. The present study is the first to apply twin-family models to study social relatedness. Analyses of twin families (MZ: N¼248; DZ: N¼75) tested evolutionary-based concepts concerning social closeness, perceived similarity and caretaking. Hypotheses based on Hamilton’s inclusive fitness theory were supported: MZ twin aunts/uncles expressed greater social closeness towards their nieces/nephews than DZ twin aunts/uncles; and female twins from same-sex pairs expressed greater closeness towards their nieces/nephews than male twins from same-sex pairs.’ | Undefined | A2i  A2ii  B1 |
| Seiden, S.C. & Morin, K. (2002) [498] | 1. USA | Medicine (4) | ‘In Schmerber v. California, the U.S. Supreme Court did find that an involuntary blood draw to assess blood-alcohol concentration was allowable without a warrant because in the time required to obtain the warrant, the evidence would have been destroyed due to metabolism of the alcohol. However, because suspects cannot destroy their own genetic identity, there would always be time for a court to evaluate whether there is sufficient evidence to issue a warrant to obtain a genetic sample. Once probable cause could be shown, it is likely that a prosecutorial request for a suspect’s DNA would be upheld. While the Supreme Court has found that forcing a suspect to have his stomach pumped to obtain evidence was unconstitutional because of its invasive nature, obtaining a DNA sample can be as minimally invasive as a cheek swab.’ | Undefined | D1 |
| Seng, E. (2003) [499] | 1. USA | Law (1) | ‘Reproductive cloning could cause alterations of the normal genetic relations in families. The cloned individual could contain solely the genetic identity of one parent with no connection to the other parent. Due to these skewed genetic relations, a cloned child may have difficulties gaining independence from his or her ‘twin’ parent. In addition to this potential imbalance within the family, reproductive cloning could lead to a greater genomic uniformity in society. This uniformity could have adverse effects to humankind’s genetic ability to allow for natural selection to operate and remove mutations and the selection of future advantages for the species.’ | Undefined | C3  C1  A1 |
| Sevini, F. et al. (2013) [500] | 3. Italy | Medicine (4) | ‘Summarizing, Wichi ́and Criollos still preserve their genetic identity and peculiar characterization as a population, despite their sharing of the environment, of some parajes and even households.’ | Undefined | A3i |
| Shadravan, F. (2013)  [501] | 1. USA | Genetics (4) | ‘Gender plays a pivotal role in the human genetic identity and is also manifested in many genetic disorders particularly mental retardation. In this study its effect on copy number variation (CNV), known to cause genetic disorders was explored.’ | Undefined | B2 |
| Shalev, C. (1998) [502] | 5. Israel | Ethics (3) | ‘In any event, the outcome of these two rules of genetic relation is a reproduction of the male model of parenthood (genetic) and a depreciation of what is unique to the female model of parenthood (gestation). The core genetic relation is the married man’s, the genetic continuity of the married woman is secondary if it does not facilitate the husband’s, and the genetic identity of the carrying mother is insignificant, aside from the requirement that she be Jewish. Having no genetic function, and agreeing to the severance of any legal relation to the child, the carrying mother becomes a symbolic receptacle for nurturing the married couple’s child.’ | Undefined | A1 |
| Shannon, T.A. (1998) [503] | Unknown | Unknown | ‘This scenario raises another dimension of the cultural presentation of the  cloning debate: genetic reductionism. (…) by simply replicating my genetic code, I am thereby replicated. However, cloning does create an offspring that is genetically identical to the donor of the DNA. But what follows from that? What follows is genetic identity: the clone is genetically identical to its source. It may even look identical. The hidden premise of genetic reductionism is that all that I need to make me ‘me’ is my genetic profile.’  ‘How else could ‘they’ be replacements for ‘us’ if ‘they’ are not genetically identical to ‘us’? To reduce such beings to commodities is to do the same to ourselves. Communality of genetic identity suggests communality of dignity as well as communality of fate.’  ‘The fact that the twins share a genetic identity says nothing about their personal identity, value, or dignity.’  ‘Through our genetic identity, we are linked to a family, to a lineage, to a history, and it is through these concrete biological realities that we establish at least part of our identity which has an inescapable biological dimension.’  ‘(…) some claim that cloning violates individuality or the individual’s right to a unique genetic identity. I argue that it is important to distinguish between genetic uniqueness and individuality and that the moral priority should be placed on individuality.’ | Undefined | B5  A2i  B1  A2ii  C1  D3  C3 |
| Shannon, T.A. (1999) [504] | 1. USA | Religion, social ethics (3) | ‘The most common scenario imagined the replication of an almost infinite series of desired genotypes on the assumption that they would essentially be the same person—all Michael Jordan clones would be superior basketball players and all James Watson clones would be superior scientists. There are two major errors in these scenarios. First, the fact that two individuals share the same genetic identity does not mean they are the same person (any more than traditionally conceived identical twins are the same person). Nor does the fact that they share a genetic identity diminish or violate the dignity of either. Second, these scenarios rest on any number of varieties of genetic reductionism that identifies the self with the genome or argues that one’s genome alone sets one’s life course and all one’s choices. Such positions deny any transcendent dimension to the person, any freedom, and simply ignore the role of environment on personal development, either behaviorally or physically. While arguments will continue over the degree of interaction of all these elements, it is clear that the major error of the human cloning debate was genetic reductionism.’ | Undefined | C3  A2i  B5  B6  B1 |
| Shapiro, M.H. (1991) [505] | 1. USA | Law (1) | ‘As for autonomy, it is hard to specify the sense in which it is invaded by the restructuring of genetic identity. One would have to invoke as an autonomy baseline the nature of the person-who-would-have-been-but-for-tampering. We do not have to resolve this, however; the more understandable threat to autonomy from germ-line engineering arises, if at all, from the risk of objectification or commodification, to which I now return.’ | Undefined | C1  C3  E5  D3 |
| Shapiro, M.H. (1999) [506] | 1. USA | Law (1) | ‘As is now well known, the Dolly method of cloning-nuclear transplantation of a diploid nucleus from an adult body cell into an enucleated ovum-does not necessarily produce an exact genomic duplicate because of the presence of mitochondria in the cytoplasm of the egg. If the ovum source cloned herself, however, the genetic template-barring mishaps—would be identical. Also, if we ever develop the technology to produce a new person from an adult diploid nucleus without having to insert it in an ovum, genetic identity could be achieved’ | Undefined | A2i  E2  C3  E3 |
| Sharma, B.R. (2004) [507] | 5. India | Medicine (4) | ‘Such an analysis was needed in order to show that having a unique genetic identity, a nervous system, a human appearance, the potential to become an adult, brain activity, or the ability to feel pain, moves an embryonic entity over the line from being ‘deserving of respect’ to having moral standing such that experimentation would violate its intrinsic rights. (…) Without knowing why certain properties count, clear boundaries between acceptable and unacceptable research cannot be drawn. From a pluralistic perspective it cannot be said whether it is right to prohibit research on an embryo after the primitive streak appearance at 14 days development. Why should research on older embryos not be allowed, if it would benefit other embryos, foetuses, children and adults (Corea, 1985)?’ | Undefined | C2 |
| Shayeb, T.Y. (2016) [508] | 1. USA | Law (1) | ‘Family, twin, and adoption studies are, perhaps, the most iconic representations of the classic approach to identifying a relationship between genes and criminal/antisocial behavior. In traditional twin studies, researchers examine the phenotypes of monozygotic twins (who share an exact genetic identity) with dizygotic twins (who share an average of fifty percent of their genetic identities), against the backdrop of shared and unshared environments. In doing so, researchers have assessed the effects of genotypes versus environments on the expression of specific human behaviors.’ | Undefined | B6  B1  A2ii  A2i |
| Shayeb, T.Y. (2017) [509] | 1. USA | Law (1) | ‘This is true regardless of whether the materials were obtained from a dining room chair or a park bench, and it matters to individuals because there is a close association between genetic identity and personal identity. The key to this concern is that many individuals want to maximize their sense of freedom/autonomy by extending their control to a tangible thing that used to be a part of them. However, since the laws of most jurisdictions in the United States do not deem the samples to be property, there is no readily recognized exclusionary right to enforce in such scenarios.’  ‘Furthermore, the argument that informed consent mechanisms are sufficient necessarily presumes that de-identification is sufficient to protect the confidentiality of the source of the samples, which is not necessarily true in instances where the samples can be traced back to their origins after the fact. The safeguards created by de-identification are founded upon the notion that genetic identity can be decoupled from social identity in a manner that assuages the concerns of private individuals an assumption which may not always be correct.(…) even if the rest of the world does not know that the sample came from a given person, the details surrounding any research conducted on those samples may be sufficient for that person to identify himself or herself in later published studies through anecdotal notes, which in itself may be harmful to the individual’s sense of privacy and autonomy.’ | Undefined | D1  B1  D3  C1 |
| Sheehan, M.J. et al. (2017) [510] | 1. USA | Neurobiology (4) | ‘We develop a simple model showing that learning a kin recognition template is sufficient to increase and maintain diversity in genetic traits used for kin recognition. Thus, our results suggest that phenotypes used for recognition may be true signals of genetic identity. As such, phenotypes are expected to evolve to facilitate recognition. Increased diversity in genetically-based recognition signals is also predicted to initiate a positive feedback loop between recognition efficiency and levels of cooperation. Finally, we discuss how the genetic architecture of recognition traits may influence kin discrimination abilities. | Undefined | A2ii |
| Sheinbach, D.M. (1999) [511] | 1. USA | Law (1) | ‘An argument for awarding rights approximating those of human beings to four- to eight-celled embryos is that these embryos are already fertilized at the time that they are preserved. Therefore, scientifically, frozen embryos have a unique genetic identity and the potential for life. In contrast to this argument, the embryos, though fertilized, have not developed to the point where they can be considered autonomous when they are cryopreserved for future implantation.’ | Undefined | C2 |
| Sheldon, S. (2005) [512] | 2. UK | Law (1) | ‘The disclosure of information sought in Rose is presented as necessary not to provide a genetic father who can replace the social father, but rather to give a further source of information about one’s own genetic identity. JR tells the Court: *these genetic connections are very important to me, socially, emotionally, medically, and even spiritually (...).’* | Undefined | A1 |
| Sheperd, L. (1995) [513] | 1. USA | Law (1) | ‘I challenge the idea that we as a society should scrutinize, judge, or otherwise censure decisions by parents regarding the procreation of children with potential genetic anomalies. In this article, I argue against the familial divisiveness of what is developing as a ‘right to be born with a sound mind and body,’ a right that while offering the promise of reducing ‘birth defects,’ holds the power of legal or moral censure against parents who allow their child’s genetic identity to be determined by the random selection of genes. In its stead I propose a right to familial attachment, a right that would permit parents to conceive and bear children with their given genetic identity, different or not, without state scrutiny or intrusion.’ | Undefined | B2 |
| Shultz, M. M. (2005) [514] | 1. USA | Law (1) | ‘The Justices disputing the applicable standard in Johnson may have intuited that a broader and somewhat different array of considerations should affect the way ARTs parentage disputes are resolved. A framework is needed that places the overall situation in perspective, one that looks not just at individual genetic identity but also at the nature of the claims and the relationships of the claimants.’ | Undefined | A1 |
| Siegel, A.M. (1994) [515] | Unknown | Law (1) | ‘As Patricia A. Martin and Martin L. Lagod note in their highly informative article titled, The Human Preembryo, the Progenitors, and the State: Toward a Theory of Status, Rights and Research Policy, ‘[t]he preembryo may have a unique genetic identity but it lacks the more developed ‘cluster of features’ which we associate with persons.’ This also holds true for any interest asserted by a clinic or other medical entity.’ | Undefined | C2 |
| Silva-Alves, H. et al. (2011) [516] | 6. Brazil | Genetics (4) | ‘Despite the geographical coexistence throughout their dispersion, each of these two Amerindian groups retains a high degree of genetic identity, probably maintained by cultural and social isolation.’ | Undefined | A3i |
| Silvers, A. & Stein, M.A. (2002) [517] | 1. USA | Philosophy (3) | ‘In sum, we do not object as strongly as Wolf to ‘seeing people as their genes because we think it possible for formal justice to acknowledge differences in genetic identity without using ‘genetic notions to privilege some individuals and subordinate others. ‘‘ ‘We therefore propose extending genetic discrimination protection to the general population by prohibiting discrimination towards individuals ‘on the basis of their genetic identity.’ Such a proscription, with language borrowed from Title VII of the Civil Rights Act of 1964 –the central protection against race or sex discrimination- would tailor genetic antidiscrimination protection to those instances when employers utilize genetic information as the grounds for inequitably reducing opportunities because of stereotypic beliefs about the significance of the individuals' genetic identity.’  ‘According to this approach to equality, characteristics of the members of one genetic classification may not be made into a standard or norm for other classes. Consequently, on this approach no particular genetic identity is privileged.’ | Undefined | B5  D1 |
| Silvers, A. & Stein, M.A. (2003) [518] | 1. USA | Philosophy (3) | ‘Just as, in principle, everybody can be identified in terms of race and sex, everybody also has a genetic identity. We take a person’s genetic identity to be constructed in terms of inheritable species-typical biological characteristics, and inheritable anomalous biological characteristics. We speak of ‘inheritable’ rather than ‘inherited’ characteristics advisedly in order to include the first generation of a mutation that might be inherited by future offspring. There are three reasons why ‘genetic identity’ should be understood somewhat broadly here. First, in the future, biologists may discover additional mechanisms of biological inheritance that are not properly ‘genetic’ but affect the inheritable constituents of people’s identity. Second, multifactorial diseases for which there is a genetic disposition should be considered to affect an individual’s genetic profile, even though nongenetic factors also come into play. Third, discrimination protection should be available in cases of adverse actions prompted by mistaken beliefs that a characteristic is genetically based, not just in cases occasioned by accurate understanding of the mechanism of biological inheritance.’  ‘Historically, certain genetic identities have been characterized as burdensome to individuals and to society as a whole. Such attributions very often emerge from admixtures of little fact and much fiction. Thus, courts should carefully scrutinize genetic categorization to protect groups that historically have been constructed as minorities subject to unequal treatment and denied opportunity on the basis of stereotypical assumptions about inheritable defects.’ | Defined | A2ii  D1  B2 |
| Singh, G. et al. (2017) [519] | 5. India | Anthropology (2) | ‘To determine the genetic affinities among five studied population groups, NJ tree was constructed (Fig. 2). The Banias and the Khatris are genetically closest to each other whereas the Jat Sikhs showed more genetic affinity with Brahmins. However, Scheduled Castes formed a separate cluster and genetically very distant from all other four groups. In the MDS analysis plot (Fig. 3), all the five studied populations are relatively close to one another and formed a compact cluster clearly separated from other populations, suggesting the genetic identity of the Punjab as a whole.’ | Undefined | A3i |
| Smith, K.H. (1999) [520] | 1. USA | Law (1) | ‘At least with respect to the cloning of human beings, while Nature converges, Nurture diverges. From the moment the organism's growth begins, differences in environmental factors will cause phenotypic divergence. (…) Further, even copies which received relatively similar intrauterine treatment may be subjected to different environmental conditions (nutrition, exercise, rest, etc.) after birth. Thus, initially identical genetic identity may nonetheless result in different phenotypic development.’ | Undefined | B6  A2i  C3 |
| Smith, L. (2010) [521] | 2. UK | Law (1) | Other arguments were framed in terms of the ethics of concealing a child's genetic identity: 'I would also like all references in the Bill that seek to create a legal fiction around parenthood to be deleted. As the Joint Committee rightly said, to deny to a child that he or she had a real biological father would be nothing short of the state colluding in a deception …’ (…) The child's right to know the identity of his/her genetic parents might well be an important issue, but whether and how this should be recognised in law is a separate issue from that dealt with in section 14(2)(b).’ | Undefined | A1 |
| Smith, L. (2013) [522] | 2. UK | Law (1) | ‘Additionally, intense social and legal emphasis on the importance of fathers and of genetic identity currently creates a climate in which it is likely that the appeal of known donors to lesbian parents will continue to have considerable traction.’ It is even possible that known donors will look more appealing now that the law provides the security of parental status for two female parents.’ | Undefined | A1 |
| Smolensky, K.R. (2008) [523] | 1. USA | Law (1) | ‘While current tort doctrine likely prohibits parental tort liability for many preimplantation genetic interventions, it does not prohibit liability in all instances. Children born as a result of direct preimplantation genetic interventions, such as genetic additions, deletions, or modifications that alter a unique set of DNA, have a legally cognizable injury if the child's genetic identity is modified in a way that limits the child's right to an open future. In these situations, concerns about parental tort immunity, procreative liberty, bodily integrity, parental decision making under the Fourteenth Amendment and the Non-Identity Problem are inapplicable. Therefore, a born-alive child harmed by direct genetic interventions should be able to sue his parents successfully for battery where the parents intentionally engage in a process that is substantially certain to make a harmful or offensive contact with the embryo, and to cause legal harm to the later-born child. | Undefined | C3  C1 |
| Smolin, D.M. (1990) [524] | 1. USA | Law (1) | ‘Tribe considers and largely rejects two arguments against personhood. First, he discusses the views of Dr. Charles Gardner, who argues that genetic identity or uniqueness, though formed at fertilization, is not equivalent to personhood because it is a mixture of chance and planning (genetics) that produces our uniqueness. (…) Gardner's statement that the development of the embryo is a combination of genetics and future events, or chance, is apparently simply an acknowledgement that each organism interacts with environment over time, and is thereby changed. Indeed, since this principle is true for both embryo and infant, Gardner's argument seems to support the pro-life position that both are equally individual human organisms.’ | Undefined | C2  B6 |
| Solis, L. (2015) [525] | 1. USA | Law (1) | ‘According to one scholar: … *It is one matter for children not to know their genetic identities as a result of unintended circumstances. It is quite another matter to deliberately destroy children's links to their biological parents.*  The right to know one's biological parents should not be decided by someone else on one's behalf. One of the most fundamental human rights of all is a child's right with respect to his biological parents, and that right must be recognized.’ | Undefined | A1 |
| Somverville, M. (2010) [526] | 1. Canada | Law (1) | ‘It is one matter for children not to know their genetic identities as a result of unintended circumstances. It is quite another matter to deliberately destroy children's links to their biological parents, and especially for society to be complicit in this destruction.’ ‘And it is not just these children who have this right, but their descendants as well. Children deprived of knowledge of their genetic identities-and their descendants-are harmed physically and psychologically.’ | Undefined | A1 |
| Sperling, S. (2007) [527] | 1. USA | Law (1) | ‘In their vote, the citizens unanimously urged caution in all forms of genetic testing. They asked the state to strictly regulate private genetic testing services and to educate the public about the risks and benefits of new technologies. This education would enable citizens to make autonomous decisions about whether to undergo tests, or, in another Kantian echo, to make them miindig. They rejected mandatory screening by the state, and by employers and insurance companies, because such sensitive data are too vulnerable to misuse. No one should have the power to reduce human worth to a statistical representation, they said, or to select between acceptable and unacceptable genetic identities.’ | Undefined | D1 |
| Spiecker, I. et al. (2016) [528] | 2. Germany | Law (1) | ‘The DPD of the EU defines personal data as 'any information relating to an identified or identifiable natural person; an identifiable person is one who can be identified, directly or indirectly, in particular by reference to an identification number or to one or more factors specific to his physical, physiological, economic, cultural or social identity', Article 2 (a). The definition under the GDPR remains largely unchanged but contains some clarifications, because it explicitly includes location data, online identifiers and genetic identity as further potential identifiers.’ | Undefined | D1 |
| Spieth, P.T. (1974) [529] | 1. USA | Genetics (4) | ‘A brief analysis is presented for the effects of gene flow upon genetic differentiation within and between populations generated by mutation and drift. Previous results obtained with the ‘island’ model are developed into a form that lends itself to biological interpretation. Attention is focused upon the effective local population size and the ratio of the genetic identity of two genes in different populations to that of two genes in the same population. The biological significance of this ratio, which is independent of population size, is discussed. Similarities between the results of this model and those of the ‘stepping-stone’ model are noted.’ | Undefined | A3ii |
| Spoerl, J.S. (2000) [530] | 1. UK | Philosophy (3) | ‘A child comes to exist (at the earliest) at conception, when a sperm and egg  cell unite to form a new, growing organism linked by genetic identity and continuous development to the mature human it will one day become (barring any disruption of its development). An as-yet-unconceived child is a merely possible, not an actual, entity.’ | Undefined | C2 |
| Stanghellini, G. & Rosfort, R. (2010) [531] | 3. Italy | Biomedical sciences (4) | ‘More than other living creatures, human beings are constituted and characterized by the interplay of their genotype and phenotype. There appears to be an explanatory gap between the almost perfect genetic identity and the individual differences among humans. One reason for this gap is that a human being is a person besides a physiological organism. Personal identity is ambiguous and fragile (Olson, 1997, 2007; Baker, 2007; Frank, 2007; Ricoeur, 1950, 1960, 1990; Fuchs, 2000; Boniolo, 2005), and human vulnerability to several mental illnesses is related to this fragile identity (Kraus, 1982; Stanghellini, 2008). Evolutionary and neuroscientific explanations of human nature and mental disorders are fraught with methodological difficulties (Dupré, 2001; McDowell, 2009; Rovane, 2004; Putnam, 1994; Thornton, 2007; Fullford et al., 2006), because too little attention is paid to this complex fragility of personal identity. Similarly, philosophical accounts of personhood and personal identity only rarely take into account the subpersonal, i.e. neurobiological, underpinning of being a human person. This problematic relationship between philosophical and neuroscientific approaches to human nature is an updated version of the time-hallowed question of first-person and third-person explanations of human nature.’ | Undefined | B1  B2 |
| Stanley, B. (1999) [532] | 2. UK | History (3) | ‘ (…) in England national consciousness preceded the unification of the kingdom; in France and Scotland, the kingdom preceded the nation; the Germans and Slavs took the most dangerous route to the nation-state by founding it on a mythical genetic identity.’ | Undefined | A3i |
| Starck, C. (2006) [533] | 2. Germany | Law (1) | ‘The identity argument is countered with the assertion that genetic identity alone is insufficient to define a human being and that the embryo does not yet meet the description of a human being because it lacks required elements such as a developed brain. This argument ignores the fact that every embryo has carried a programme for such brain development since the moment of his creation. It is equally inappropriate to deny the existence of a genetic identity by pointing out the occurrence of genetically identical twins, because in such an event two people with identical genetic make-ups will need to be protected who will grow up to be separate and independent individuals in possession of their fundamental rights.  ‘The fertilized human egg (embryo) is a person and belongs to the human species as soon as the cell nuclei have merged. The embryo's genetic programme provides the potentiality for the development as a human being that occurs continuously and not in discernible stages with clearly marked cutoff lines, beginnings or ends. The identity between embryo and newborn child is a genetic identity. This is all we need to establish since the later development of the human being is subject to a wide range of influences that can - and, of course, do - lead to continuous changes in the fully-fledged identities of individuals. Since the fertilized egg represents individual human life, it is fully entitled to the constitutional protection of life and dignity.’ | Undefined | B7  C2  B6  C1  A2i |
| Stark, M. et al. (2006) [534] | 4. Australia | Medicine (4) | ‘p33ING1b mutations in melanoma are rare. We have highlighted the importance of allele-specific primer design to avoid pseudogene amplification, and also the necessity to confirm the genetic identity and species of origin of individual cell lines. Further studies are needed to clarify the possible role of p33ING1b in melanoma tumorigenesis. | Undefined | F1 |
| Steinbock, B. (2005) [535] | 1. USA | Philosophy (3) | ‘Perry-Rogers v. Fasano was a case in which medical error led to implanting the wrong embryos into a woman, causing an ‘accidental surrogacy’. (…) The Rogerses attempted to contact the Fasanos, but the Fasanos did not respond. Nor did Mrs. Fasano undergo any testing to find out the genetic identity of the babies she was carrying. However, the truth became obvious on December 29, 1998, when she gave birth to two male infants, one white and one black.’ | Undefined | A1 |
| Stenger, R.L. (2006) [536] | 1. USA | Law (1) | ‘Support for not naming an embryo a person may also be grounded in the  common intuition that the death of an embryo or fetus is different from the death of an infant. To call a bundle of dividing cells a person is rather a conclusion drawn from biological evidence of genetic identity between fertilization and birth. The problem with identifying personhood with the arrival of consciousness is that one can date consciousness at cortical EEG activity (21-22 weeks), or visual and auditory response (at 24 weeks), or cerebral functioning (28 weeks)…’ | Undefined | C2 |
| Stenzel, P. (2009) [537] | 1. USA | Law (1) | ‘A second criticism comes from adoption rights groups. They assert children have the right to know their genetic parents' identities. Groups like Bastard Nation advocate for adoptees to have unconditional access to their adoption records. These groups see the right to know one's parents' identities as both a political issue and a fundamental human right. Relinquishments under Safe Haven laws undermine a child's right to know his or her genetic identity.’ | Undefined | A1 |
| Stinson, C. (1972) [538] | 1. USA | Religion (3) | ‘Would this duplicate have an ‘individual soul’ — that is, would he or she have personal ethical, aesthetic and religious experience? (..) Each cloned individual would be a separate psychosomatic organism with a lived-experience of his or her own, and thus capable of the full range of human feelings. Though very strange to us now, the genetic identity of these clones and their mode of production would in no way affect their genuine humanity provided they were raised in a loving familial environment. In principle, their case would seem to be not much different from that of identical twins or triplets. The chances are that Ramsey's fear of ‘depersonalization’ and lack of ‘embodied personhood’ will turn out to be unjustified. | Undefined | C3  B3  A2i  B6  B1 |
| Stormann, T.M. et al. (1989) [539] | 1. USA | Moleculair biology (4) | ‘These data indicate that D2 receptors expressed in the inner retina and outer plexiform layer have genetic identity with those expressed by brain and that the human and rat D2 receptors are derived from highly related genes.’ | Undefined | F1 |
| Stringer, R. (2006) [540] | 4. New Zealand | Gender studies, political studies (2) | ‘As the following passage from Harrild indicates, the connected tissue configuration specifically serves to reject the separate entities model, and represents an effort to recognize integrity and interconnection simultaneously. As such it may be regarded as a version of Karpin’s not-one-but-not-two model:  *This reasoning for treating the fetus as separate from the mother comes down to the lack of common genetic identity. That must certainly be accepted as a matter of scientific fact. But it is also a fact that a fetus comprises human tissue which is connected to the mother while it is inside her.*’ | Undefined | C2  A2i |
| Stromberg, A. et al. (1999) [541] | 2. Sweden | (Bio) chemistry (4) | ‘A simple way to obtain only the desired fusion product is to work with individual cells. The ability to fuse together single cells in a controlled manner represents a technique by which the long-term genetic identity and behavior of a select cell can be precisely manipulated. In combination with powerful measurement and imaging techniques, the genetic and biochemical nature of single cells can be controlled and studied in detail.’ | Undefined | F1 |
| Stubbs, J.K. (2005) [542] | 1. USA | Law (1) | ‘*The categories we acknowledge as races are marked by any number of differences, but the biological differences between them are minimal, reinforced by social and cultural differences*. In short, there is an essential genetic identity at the core of each human being which reflects our overwhelming similarities, and distinguishes us (human earthlings) from other earthlings.’ | Defined | A2ii  B7  A3i |
| Styner, M. et al. (2005) [543] | 1. USA | Medicine (4) | ‘The analysis includes ventricle shape comparison between pairs of co-twins to examine shape similarity in relation to genetic identity.’ | Undefined | A2i |
| Suter, S.M. (2009) [544] | 1. USA | Law (1) | ‘Finding a heretofore unknown genetically related half sibling filled a personal gap that each had felt due to being cut off from half of their genetic identity.’  ‘While there is a danger in believing that our genetic identity is our complete identity, and while our society may increasingly overstate the value of genetic information in self-definition, we should not reject the value and interest in genetic identity. It is not merely a social construct that should be discouraged; it is a way to connect to one's ‘biological past.’ An interest in one's biological parents is not always based solely on a desire for medically relevant information. The desire to know one's genetic heritage is part of a complex discovery of identity, understood in relation to the many who shape us: intimates, family, and community. The information provides additional background to [the children's] full identities -genetic, emotional, and even cultural. Our environment and social relationships are clearly central to self-definition, but so is our genetic information. Both pieces of the nature/nurture puzzle are important in self-understanding and self-definition, especially if we understand the self in relational terms.’ | Undefined | A1  B1  B5  B6 |
| Szostak, D.C. (2010) [545] | 1. USA | Law (1) | ‘Control over biological materials must be in the hands of researchers and  medical professionals. Those who come down on the side of property rights in body parts will argue that people ‘have greater rights in their likenesses, and to privacy of their medical records than over the commercialization of their genetic identities,’ which may seem unsettling. Similarly, they may claim that while ‘we speak often about autonomy and liberty, there is no legal guarantee of integrity over either our bodies ... or over our genetic identities.’ However, people in the scientific community know how best to use these resources, and regulations exist that prevent their misuse.’ | Undefined | D3  C1  D1 |
| Szyf, M. et al. (2009) [546] | 1. Canada | Pharmacology (4) | ‘We will discuss here the current links between the regulation of the DNA methylation machinery and DNA damage responses and propose a unifying hypothesis for the mechanisms developed to protect our epigenetic and genetic identity of a cell. These have implications on or understanding how environmental agents exert their genomic damage, as well as on chemotherapeutics and toxicology. DNA should be understood in a wider context of damage to the genome as well as to the epigenome.’ | Undefined | F1 |
| Tabarssi, M. (2000) [547] | 1. USA | Religion (3) | ‘Many medieval Western theologians made the ridiculous assertion that Muhammad (P.B.H.) had invented polygamy when in fact, the practice had predated Islam (…). Early societies, being constantly engaged in hunting and war, became depleted of males. The resulting excess of women compelled a choice between polygamy and the barren celibacy of a minority of women. Social divisions within a group of wives were also created in order to preserve some sense of genetic identity.’ | Undefined | B1  A2ii |
| Tafur-Dominguez, V. (2000) [548] | 6. Colombia | Law (1) | ‘Products that are not subject to patent, in addition to the pharmaceutical products on the WHO's list of essential medicines, include inventions that threaten the public order or morals, the health of man and animal, and environmental and plant conservation. This category also encompasses animal breeds and species along with the biological procedures for obtaining them, and inventions concerning the component elements of the human body and genetic identity.’ | Undefined | D3 |
| Takenaka, K. et al. (2007) [549] | 1. Canada | Molecular Biology (4) | ‘Graft failure in the transplantation of hematopoietic stem cells occurs despite donor-host genetic identity of human leukocyte antigens, suggesting that additional factors modulate engraftment.’ | Undefined | A2i  F1 |
| TallBear, K. (2007) [550] | 1. USA | American Indian studies (2) | *‘Our genes allow us to chart the ancient human migrations from Africa across the continents. (…) The key to this puzzle is acquiring genetic samples from the world's remaining indigenous peoples whose ethnic and genetic identities are isolated. But such distinct peoples, languages, and cultures are quickly vanishing into a 21st century global melting pot.’*  ‘Native origin stories and oral histories are key for understanding who our ancestors were and how we got to where we are today. Some of these are relatively recent stories like the Dakota Conflict and its aftermath. Why genetics and genetic identities should trump those formations is not obvious.’ | Undefined | A3i |
| Taylor, P.D. et al. (2007) [551] | 1. Canada | Mathematics (5) | ‘The methods of inclusive fitness provide a powerful analysis of the action of selection on social behaviour. The key component of this analysis is the concept of relatedness R. In infinite populations, a standard method of calculating relatedness coefficients is through coefficients of consanguinity using the notion of genetic identity by descent. In this paper, we show that this approach can also be made to work in finite populations and we assume here that the population has a homogeneous structure, such as an island model.’ | Undefined | A3ii |
| Teixeira, J.C. et al. (2011) [552] | 3. Portugal | Molecular immunology (4) | ‘In the present work, we have sampled 56 unrelated individuals from the Braganc¸a Jewish community aiming to characterize their maternal lineage. A 3348 bp mtDNA fragment was amplified and sequenced using mitochondrial-specific primers in order to obtain the entire control region. Haplogroup classification was performed according to current nomenclature. High frequencies were found for haplogroups H, HV0, T2, U2 and N1, indicating some degree of European admixture along with a remarkable signature of a Near East ancestry. These data confirm that the Crypto-Jews from Braganca were able to maintain not only their cultural identity but also some ancestral genetic identity, showing a significant population substructure within Portugal, with forensic relevance.’ | Undefined | E3  A3i  B1 |
| Ter Linde, J.J. & Samson, M.  (2004) [553] | 2. Netherlands | Medicine (4) | ‘The concept that genetic variation underlies inter-individual differences in drug response and contributes to the risk of developing common, complex disorders is expanding rapidly. Consequently the interest in genetic translational research has increased. Polymorphic DNA markers, either microsatellites or single nucleotide polymorphisms (SNPs), are used to assess genetic identities and track genetic differences between individuals.’ | Undefined | B2  E1 |
| Teugels, E. & de Brakeleer, S. (2017) [554] | 2. Belgium | Medicine (4) | ‘While environmental factors can greatly increase cancer risk, it is clear that an individual’s genetic constitution has strong impact on tumor formation. (…) An additional advantage of this approach is that cancer risk assessment will not strictly rely on the individual’s genetic identity, but will also consider other factors (e.g., environmental and age) that can affect genomic integrity.’ | Undefined | B2  B6 |
| Thomas, C. (2017) [555] | 1. USA | Law (1) | ‘The potentiality of In Vitro Gametogenesis (IVG) is realized when this technique is combined with iPSC technology and thus create gametes with the genetic identity of any individual. Because iPSCs can be created from most mature somatic cell types, medical doctors and lab technicians can derive pluripotent cells from any human being. An extremely simple and practical technique to acquire adult cells is to extract skin cells from an individual by a ‘skin punch biopsy.’ Doctors or technicians can convert the skin cells obtained from the biopsy into iPSCs by modulating the gene expression. Further differentiation of the iPSCs into mature gametes would yield sperm and ova with genetic information of the skin-punched individual.’ | Undefined | C3 |
| Thornton, D.J. et al. (1996) [556] | 2. UK | Biochemistry (4) | ‘Here we extend our studies on respiratory mucins by using ion-exchange chromatography and agarose gel electrophoresis to further analyse the different populations previously described [4]. We identify one of them as a MUC5AC mucin while the genetic identity of the other(s) remains to be established. The level of the MUC5AC mucin varies greatly between respiratory samples but the molecule is in all cases similar with regard to charge density and electrophoretic mobility.’ | Undefined | F1 |
| Thorpe, J.P. (1989) [557] | 2. UK | Biology (4) | ‘To convert overall comparative data between species or populations to a single figure it is conventional to calculate one of several published statistics usually known as genetic similarity or genetic identity (measures of similarity) or genetic distance (measures of dissimilarity) (Neil 1981, 1987; Thorpe, 1982). Of these available measures by far the most widely used are the genetic identity, I, and genetic distance, D of Nei (1972).’ | Defined | A3ii |
| Tillman, J.J. (2008) [558] | 1. USA | Religion (3) | ‘In the case of kinship altruism, the actor is protecting his or her genetic identity as represented in the survival of offspring.’ | Undefined | A2ii  B4i |
| Tobin, B. (2014) [559] | 2. Ireland | Law (1) | ‘Part 4 of the revised General Scheme is a welcome revision for donor- conceived children born into Irish families, as it endeavours to vindicate their (arguably constitutional) right to knowledge of their genetic identity.’ | Undefined | A1 |
| Tofanelli, S. et al. (2005) [560] | 3. Italy | Ecology and evolution (4) | ‘However, the overall genetic identity has been preserved as our sample plotted tightly close to Moroccan populations on multidimensional genetic spaces.’  ‘We aimed at estimating (…); its effects on genetic identity, the ethnic-specific profile shaped upon Y-chromosome genes.’ | Defined | A3i  E4 |
| Tomiuk, J. et al. (1998) [561] | 2. Denmark | Evolutionary Biology (4) | ‘Different genetic identity or distance measures are compared that consider allelic variation within and between populations. Particularily we analyse those suggested by Nei… (…) The simulations focus on the influence of non-equilibrium conditions on the stability of these measures. The degree of homozygosity of an ancestral population before it splits into two sister populations is most important for the stability of the different estimates of genetic identity.’ | Undefined | A3ii |
| Torres-Rodrigues, M. et al. (2006) [562] | 6. Mexico | Genetics (4) | ‘The pentanucleotide STR (TAAAA)n DXYS156 offers advantages for genetic identity testing. In addition to establish the gender, DXYS156 expands the DNA profile and is able to indicate the possible geographic origin of the individual. We analyzed DXYS156 in 757 individuals of both sexes from Mexican populations. ‘ | Undefined | A3i  D1 |
| Troiano, S. (2013) [563] | 3. Italy | Law (1) | ‘When the knowledge of maternal origins is necessary to assess the child's genetic identity and thus protect his or her health, there should, indeed, be free access to this information at any time. Nevertheless, the information obtained should not be used to retrieve the mother's identity (…)’ | Undefined | A1  D1 |
| Trujillo, A.C.L. (2006) [564] | 3. Vatican | Religion (3) | ‘We might even say that this is something genetically determined since the embryo proceeds from the contribution of the father and the mother. The XX and XY chromosomes (corresponding to the female and male genetic identity) are a gift of inheritance.’ | Undefined | A2ii  B4 |
| Tsosie, R. (1999) [565] | 1. USA | Law (1) | ‘Indigenous peoples have expressed indignation at the inference that scientific documentation of their genetic identity can substitute for actual protection of their distinctive cultural and political identity.’  ‘… and second, that Kennewick Man's culture is not ascertainable without establishing his genetic identity. In other words, the scientists assert that any contemporary cultural claim must rest on biological proof of a racial connection between the ancient skeleton and the modem group.’ | Undefined | A3i  B1 |
| Tsosie. R. (2005) [566] | 1. USA | Law (1) | ‘Current research dealing with population genomics and the origins of human populations raises several challenges to Native American identity based on a blend of scientific and legal attacks. This research places a heightened emphasis upon ‘genetic identity’ in accordance with contemporary scientific analysis, but in reality, this research constitutes a twenty-first century manifestation of a very old phenomenon in American social politics: the construction of race.’  ‘Although it is no longer considered ‘politically correct’ to assert that there is a biological basis for ‘race,’ the scientific construction of race in the twenty-first century continues through efforts to document the genetic identity of distinct groups, a process often referred to as ‘population genomics.’ Much of this research purports to reveal the susceptibility of certain ‘groups,’ such as African-Americans or Native Americans, to certain diseases, such as sickle cell anemia or diabetes.’ | Undefined | A3i  B1  B2 |
| Tsosie, R. (2012) [567] | 1. USA | Law (1) | ‘As such, this ancient individual belongs to ‘science,’ which is the body of knowledge that can tell us the truth as a matter of genetic identity about who Kennewick Man really was and cast some light on the contentious issue of the ‘peopling of the Americas.’ (…) The testimony of the tribal claimants is entirely disregarded as ‘mythology’ and ‘religious ideology,’ while the scientific data represented by genetic testing is understood to have the capacity to tell us the ‘truth’ about human origins and identity.’ | Undefined | A3i  B1 |
| Tsosie, R. (2016) [568] | 1. USA | Law (1) | ‘According to population geneticists, the transborder population within the Southwest Borderlands region shares a genetic heritage. But, the genetic identity (ancestry) of these individuals is irrelevant within the cultural politics of the Borderlands, which treats indigenous peoples separately with regard to race and political status.’ | Defined | A3i B1 |
| Tupasela, A. & Tamminen, S. (2015) [569] | 2. Denmark | Sociology (2) | ‘The question of where species and populations come from and the linking of genetic traits to geographical locations have, however, resurfaced as both scientific and political sites of interest more recently through the study of population genetics in both human and non- human genetics (Cavalli-Sforza et al. 1994; Dutton 2008; Tamminen 2010; Whitmarsh and Jones 2010). These processes can be seen as attempts to stabilize and naturalize the genetic identity of various populations within specific geographical locations by laying political claim and sovereignty over them (Hinterberger and Porter 2015).’ | Undefined | A3i |
| Turkmendag, I. et al. (2008) [570] | 2. UK | Sociology (2) | ‘(…); and third, that family relationships (particularly father–child) would be damaged if the child’s real genetic identity were revealed (Gottlieb et al, 2000; Lalos et al, 2007).’ | Undefined | A1 |
| Turkmendag, I. (2012) [571] | 2. UK | Sociology (2) | ‘The promotion and protection of children’s right to know their genetic identity, drawing on the principle of best interests and also perhaps on human rights, is now embedded in the donor conception regulations of a number of countries. Sweden was the first country to remove donor anonymity in 1985, and similar laws have since been passed in a number of countries including Austria, Germany, Switzerland, New Zealand, the Australian states of Victoria and Western Australia, the United Kingdom, The Netherlands, and Norway. One might argue that the donor conception amendments in Turkey seem to reflect a European ethos in that they are concerned with children knowing their origins.’ | Undefined | A1 |
| Tuya, C. et al (2006) [572] | 2. UK | Medicine (4) | ‘Twins can be used to investigate the biological basis for observed associations between birth weight and later disease risk, as they experience in utero growth restriction compared with singletons, which can differ in magnitude within twin pairs despite partial or total genetic identity.’ | Undefined | A2i  A2ii |
| Urban, E. (2013) [573] | 1. USA | Religion (3) | ‘This argument is based on other Qur’anic pronouncements about kinship and marriage, for instance the saying Q. 25:54, *He has made for him relation by descent and relation by marriage – for your Lord is Omnipotent.* That is, blood relationship is part of the glory and mercy of God’s creation; genetic identity is a God-given principle, not a man-made one.’  ‘In particular, verses 5-6 create a complicated relationship between genetic identity and religious identity, and they seem to affirm an Islamic social structure based not just on faith but also on blood relationships. This complex connection between genetic identity and religious identity would persist into the early Islamic period, as the class known as the *mawali* continued to navigate their simultaneous identities as Muslims and foreigners, as insiders and outsiders.’ | Undefined | B3 |
| Vaisman, N. (2014) [574] | 2. UK | Law (1) | ‘In Evelyn’s case, the Court sided with the plaintiff and did not require her to undergo a blood test to verify her genetic identity, arguing that if an adult is not interested in learning her `true identity’, the Argentine legal system cannot force her to do so.’  ‘Their use of the term `true identity’ is revealing of the significance genetic patrimony has for their understanding of the subject. In other words, the Court views the individual’s genetic identity and his identity in the social world as the same.’ | Undefined | B1  A1  B5 |
| Valongo, A. (2014) [575] | 3. Italy | Law (1) | ‘As a consequence, the embryo should be treated not as a tool to satisfy the needs and interests of others, as something to be disposed of by third parties, but rather as an individual with rights, such as the right to remain alive and follow a natural evolution towards birth, the right to life, the right to health and the respect of genetic identity and integrity.’ | Undefined | C2 |
| Van Baak,T.E. et al. (2018) [576] | 1. USA | Medicine (4) | ‘Despite their seemingly unsupportive findings in mono- chorionic vs. dichorionic twins, Kaminsky et al. proposed that in addition to their genetic identity, ‘epigenetic similarity at the time of blastocyst splitting may also contribute to the phenotypic similarities in MZ co-twins,’ exactly as our findings suggest.’ | Undefined | A2i  B6 |
| Van Hove, J.L. et al (2005) [577] | 2. Belgium | Pediatrics (4) | ‘The clinical presentation is consistent with Clericuzio type poikiloderma with neutropenia. Literature review identified several additional probable patients. Genetic linkage analysis excluded the locus of the RECQL4 gene, mutations in which have been described in some patients with the Rothmund–Thomson poikiloderma syndrome. This report confirms the clinical and genetic identity of the Clericuzio type of poikiloderma with neutropenia syndrome.’ | Undefined | F1 |
| Van Wichelen, S. (2016) [578] | 4. Australia | Sociology (2) | ‘Another argument proffered in the discourse on parentage is that a child’s genetic identity forms part of a child’s history. There may be medical advantages in the children knowing their parentage.’ | Undefined | A1 |
| Van Wietmarschen, H. et al. (2006) [579] | 2. Netherlands | Integrative Health (4) | ‘If we define identity as concerned with the binding together of people, either by individual self-affiliation or as a result of categorization by others, as members of a particular group (Elwert 1997:727), then a genetic identity may be defined as the binding together of people on the basis of a particular genetic characteristic they share. This gives birth to the hypothesis that new social- cultural realities may emerge when people organize themselves around specific genetic knowledge (Koot 2003:7). The question is how ‘genetic tests’ and the production of ‘genetic knowledge’ is related to the development of ‘genetic identities’ and the corresponding new socio-cultural realities (Waldschmidt 2005; Stiker 1999).’  ‘New genetic identities can be based on genetic information that is directly related to an actual experience, such as a disease that is present in the body. However, genetic identities can also be based on potentialities. People who experience their bodies as perfectly healthy can stumble upon genetic information that tells them otherwise. Genetic tests can reveal risks and potential diseases that contradict the way people have been experiencing their own bodies up to that time (Horstman 1999; Zwieten 1999).’ | Defined | B1  B2  A2ii |
| Villiers, J.D. (2010) [580] | 1. USA | Law (1) | ‘Other countries currently use DNA testing in family reunification cases. France statutorily implemented the use of DNA testing in 2007, and Switzerland has used DNA testing since 2004. Further, several other European countries use such tests in family reunification cases. Even if the current non-mandatory testing policy remains the same in the United States, genetic identity will likely be viewed as paramount, a reflection of the applicant’s real identity. Moreover, relationships forged by genetic connections may be seen as superior to those established by other unscientific means.’ | Undefined | A1  B1 |
| Vinck, S.C. (2003) [581] | 1. USA | Law (1) | ‘Another argument for a human cloning ban is that ‘an impact on internal autonomy will have a direct and proportionate impact on external autonomy and the exercise of one’s civil liberties.’ This is plausible, since a clone’s knowledge of his genetic past would lead him or her to a sense of entrapment. This internal barrier would eventually become an external hindrance; the sense of powerlessness would deter a cloned person from taking advantage of the full scope of social, economic, and political privileges of a free society.  Thirteenth amendment.’’ | Undefined | C1  C3 |
| Visscher, P.M. (2009) [582] | 4. Australia | Genetics (4) | However, if the actual proportion of genes that account for quantitative trait variation in the genome varies between pairs of relatives with the same values of a and d, then the phenotypic covariance will vary accordingly. Hence, among all pairs of relatives with the same expected genetic identity, the pairs that share more alleles at trait loci IBD are expected to be phenotypically more similar. | Undefined | A3ii |
| Volpi, L. et al. (2010) [583] | 3. Italy | Biology and genetics (4) | ‘Next-generation sequencing is a straightforward tool for the identification of disease genes in extended genomic regions. Autozygosity mapping was performed on a five-generation inbred Italian family with three siblings affected with Clericuzio-type poikiloderma with neutropenia … (…) Two distinct deleterious mutations (c.502A>Gand c.666_676þ1del12) identified in an unrelated PN patient confirmed that the G16orf57 gene is responsible for PN. The function of the predicted G16orf57 gene is unknown, but its product has been shown to be interconnected to RECQL4 protein via SMAD4 proteins. The unravelled clinical and genetic identity of PN allows patients to undergo genetic testing and follow-up.’ | Undefined | F1 |
| Wade, K. (2017) [584] | 2. UK | Law (1) | ‘ It is clear that there is a growing recognition of the importance of telling children about their origins at an early age to ensure that they develop an integrated and narrative sense of self. It has been noted that donor-conceived individuals have consistently, although not universally, reported the need to know their genetic origins, and studies indicate that non- disclosure can lead to psychological damage, low self-esteem and issues relating to trust, for example. The importance of knowing one’s genetic identity was mphasized in Rose v Secretary of State for Health and Human Fertilisation and Embryology Authority. In this case, it was held that Article 8 of the European Convention included a right of access to information about biological parents in the context of donor conception, which in turn led to legislative change eliminating donor anonymity.’ | Undefined | A1 |
| Wagner, J.K. (2009) [585] | 1. USA | Law (1) | ‘When individuals recognize the naïveté of genetic determinism and begin to understand both the complexities of gene-gene and gene-environment interactions and the distinctions between various types of genetic information or tests, the expectation of genetic privacy will necessarily diminish-at least for some loci. While there is unquestionably a reasonable expectation of privacy in one’s medically relevant genetic information, the reasonableness of an expectation of genetic privacy solely in identification markers is far from certain. There is a compelling argument that increasing our knowledge of genetic identities, specifically via a national database, ‘will promote ‘racial justice,’’ rather than promote racial profiling or discrimination on a genetic level. Just as highlighting every sentence on a page is the equivalent of highlighting nothing, so too would be a national database.’ | Undefined | D1  B5  B6 |
| Wallbank, J. (2002) [586] | 2. UK | Law (1) | ‘I have argued that this occurs at the expense of due consideration of the welfare of the child in one very significant way, i.e. the interests of children in having access to knowledge of their genetic identity or mode of conception and birth, including knowledge of the surrogate.’ | Undefined | A1 |
| Walsh, J. (1994) [587] | 1. USA | Law (1) | ‘Gene therapy may be the most controversial of the three because of the fear that human gene therapy may be misused or have permanent effects upon the germ line. However, the fear that gene therapy may have some impact on the genetic identity of the human species must be weighed against the benefit of providing treatment for common and catastrophic diseases’ | Undefined | C3  B7 |
| Walther, S. (1993) [588] | 2. Germany | Law (1) | ‘ Again, the Court held that human life in its genetic identity and uniqueness exists at the conclusion of nidation.' From this time on, the fetus develops ‘as a human being’, and all human life enjoys human dignity.’ | Undefined | C2 |
| Wang, Y. et al (2012) [589] | 5. China | Stem cell research (4) | ‘We describe the derivation and characterization of three novel human embryonic stem (hES) cell lines (YT1, YT2, YT3). (…) Pluripotency was confirmed by in vitro and in vivo differentiation, and genetic identity was demonstrated by DNA fingerprinting. Our results indicate that higher concentrations of bFGF at the early culture stage support efficient the hES cell derivation. | Undefined | F1 |
| Wardle, L. D. (2007) [590] | 1. USA | Law (1) | ‘Similar problems exist in a frequently cited study that purported to show a correlation between homosexuals and genetic identity. Professors Michael Bailey and Richard Pillard compared male identical twins, fraternal twins, non-twin brothers, and adopted brothers for sexual orientation. (…) Because the most closely related siblings had the highest concordance rate, they interpreted their results to support their thesis that homosexual behavior has a genetic basis.’ | Undefined | A2ii  B1 |
| Waring, Y.E. (2005) [591] | 1. USA | Law (1) | ‘The DNA chemical molecule is found in all life forms, contains the complete genetic information unique to that form, and can hold this information for decades -thus, making it possible to test and utilize evidence in cases that have been left unresolved for years. Even more phenomenal, DNA ‘profiling’ has made it possible to indict and charge the genetic identity of the accused without knowledge or custody of the person.’ | Undefined | D1  D2 |
| Warren, M. (2009) [592] | 4. Australia | Law (1) | ‘The techniques and equipment used by the initial forensic pathologist, such as the ortho-tolidine test, to identify the presence but not the genetic identity of the blood, have continued to be developed and improved which in turn has increased the reliability of forensic medicine as an investigative tool.’ | Undefined | D1 |
| Webster, W.R. Jr. (2000) [593] | 1. USA | Law (1) | ‘The concept of genetic identity presents new constitutional questions never imagined before Watson and Crick discovered the double-helix. These issues concern privacy and whether it can be protected in the age of The Human Genome Project.’ | Undefined | D1 |
| Weeden, J.L. (2006) [594] | 1. USA | Law (1) | ‘This note uses the phrase ‘*genetic identity’* in a broad sense as connected to an individual’s persona. A Dictionary of Genetics provides a more technical definition of ‘genetic identity’ as ‘a measure of the proportion of genes that are identical in two populations.’’  ‘If a person does not own his genetic material, he can neither own nor protect his genetic identity, thus leaving it in peril indefinitely. If no one owns something, then, by legal definition, no one can be guilty of stealing it. How can we best protect our genetic identity? The only way to protect genetic identity from theft is to create a legal property right in the genetic information that secures this identity.’  ‘The power of DNA evidence in criminal investigations may place many of our most precious liberties in jeopardy. There is great peril of community discrimination and stigma regarding a person’s genetic identity.’  ‘Expansion of a privacy right in genetic identity or persona is a way to protect an individual’s genetic information.’ | Undefined | B1  D1  A2i  A3i  D3 |
| Wei, C.C. et al (1999) [595] | 5. Taiwan | Medicine (4) | ‘The genetic identity analysis and the genetic distance analysis reached the same conclusions, viz., that the Ami and the Paiwan tribes were genetically close to each other, that the Atayal tribe was relatively unique compared with other tribes, and that the Saisiat tribe was relatively close to the Han-Taiwanese.’ | Undefined | A2ii  A3i |
| Wei, A.H. et al. (2013) [596] | 5. China | Molecular Biology (4) | ‘More candidate OCA genes in humans are likely to be identified as suggested by additional mouse OCA genes (Bennett and Lamoreux, 2003; Li et al., 2006). In addition, unknown loci are implicated in human OCA patients. Recently, a region on 4q24 has been identified as an additional locus (OCA5) for nonsyndromic OCA (Kausar et al., 2012). However, its genetic identity has not been characterized. It is plausible to predict that unknown OCA genes will be uncovered.’ | Undefined | F1 |
| Weiner, K. (2011) [597] | 2. UK | Medical sociology (2) | ‘Overall, the paper raises questions about people’s preparedness to assume genetic identities and forms of responsibilities. I have suggested that this may depend, to some extent, on the specific characteristics of the condition in question, including the types of diagnostic technologies employed, the availability of prophylactic therapies, the prevalence of the condition, and the nature of lay models of aetiology and types of clinical discourse and practice with which it is associated.’ | Undefined | B2 |
| Welstead, M. (2003) [598] | 2. UK | Law (1) | ‘Acceptance is growing that truth about genetic identity is not only important but also difficult to suppress. It is not merely a question of sentimentality but also one of practical importance involving issues of genetic inheritance. However, proof of genetic paternity should remain a separate issue from the grant of paternal rights particularly where the child has established a strong relationship with an alternative father figure.’ | Undefined | A1  D1 |
| Wevers, K. (2010) [599] | 1. USA | Law (1) | ‘A provider of PGD services is causally connected to the birth of a particular child in the sense that the provider selects and implants that child's genetic identity.’ | Undefined | C3 |
| Wiegers, W. (2010) [600] | 1. Canada | Law (1) | ‘Henry argues that if support from non-biological fathers is denied, the mother and child (or the state) can still seek support from the biological father, and that the child's best interests are in any case served by knowing his or her ‘real’ father. Support can also be reduced by contributions on the part of the genetic father. However, neither this fact nor the benefits of knowing genetic identity preclude recognition of a de facto father. Henry's position implicitly assumes that a child can have only one father, and understates the importance of relational  bonds.’ | Undefined | A1 |
| Will, J.F (2003) [601] | 1. USA | Law (1) | ‘Unlike fingerprints used for identification purposes, however, an individual's DNA contains a myriad of information about health and genetic identity that deserves increased protection.’ | Undefined | D1  B2 |
| Will, J.F. (2013) [602] | 1. USA | Law (1) | ‘In addition, for those who find meaning in the concept of individual genetic identity (numeric or otherwise) as it relates to defining members of the Homo sapiens species, further discussion could be had regarding genomic alignment, cell differentiation, and/or the process of twinning.’ | Undefined | B7 |
| Wilson, M.L. (2009) [603] | 1. USA | Law (1) | ‘Another consideration is whether the individual being tested, or the parent of the child who is tested, is capable of living with the knowledge that he, or his child, is plagued with a genetic identity that will likely lead to disease. Parents who are carriers of genetic diseases may feel desperate and guilty for passing on a disease to their children. ‘Studies have shown that knowing that one is at risk for genetic conditions or even learning that one does not have [a defective gene] strongly affects self-perception and life experiences.’’ | Undefined | B2 |
| Wood, P.G. (1999) [604] | 2. UK | Law (1) | ‘When the genome - the complete genetic identity of any individual - is cloned (or copied) a genetically identical individual is created.’ | Defined | E6  A2i  C3 |
| Wright, R.G. (2000) [605] | 6. Chile | Law (1) | ‘Would anyone say that whatever the positive moral value of blood or organ donation, against that must be balanced not only the minimal loss in genetic diversity, but the loss by both donor and recipient of their basic human dignity, given their now shared genetic identity?’  ‘In fact, human cloning and its associated technical machinations should typically, if quite inadvertently, operate to clarify the nature of human dignity and to heighten our awareness of and appreciation for human dignity. Just as intense heat may serve to purify a metal, so human cloning may clarify what is really essential to human dignity, and what things--including a unique genetic identity-are not. Once the dross of genetic uniqueness and of difference in appearance is largely, if not entirely, burned away, we are left with something more nearly approaching the mystery of human dignity itself.’ | Undefined | C3  A2i |
| Wu, D. et al (2009) [606] | 1. USA | Medicine (4) | ‘Recent studies have shown that pluripotential bone marrow and mesenchymal stem cells readily traverse to and from transplanted organs, suggesting that the mechanisms of donor-recipient integration in the post-transplant state are dynamic and complex. Current methods used to assign genetic identity in situ in these situations are typically limited to gender-mismatched cases, in which the Y chromosome is analyzed by fluorescence in situ hybridization (FISH) in a female genetic background.’ | Undefined | F1 |
| Wynn, L. (2014) [607] | 1. USA | Law (1) | ‘With further advancements in biotechnology and genetic engineering, a significant concern globally is the right to individual identity in human rights law when an individual desires to voluntarily modify his or her genetic makeup. These advancements have required the international legal community to consider the concept of genetic identity within the encompassing view of the right to identity. ‘Genetic attributes are seen as only a part (albeit important) of someone's identity, not equating to the whole of the identity itself.’ Because genes are shared, to a certain extent, by every being in the world, genetic engineering and modification raises issues not only with the ‘interests of the individual human person, but also the interests of groups and future generations.’’ | Undefined | C1  C3  B1  A2ii |
| Yoshino, K. (1998) [608] | 1. USA | Law (1) | ‘Ely's position is supported by the fact that groups sometimes defend themselves by proving immutability rather than disavowing it. The rising fascination with genetic identity may be partially explained by an interest in this strategy. Stigmatized traits that have been attributed to genetics include ‘mental illness, homosexuality, aggressive personality, dangerousness, … exhibitionism, the tendency to commit arson,... [and] shyness.’’ | Undefined | B1  B2 |
| Yoshizawa, A. et al (2006) [609] | 5. Japan | Medicine (4) | ‘A possible explanation for the early re-infection and progression of acute hepatitis was the genetic identity between donor and recipient.’ | Undefined | A2i |
| Zafran, R. (2008) [610] | 5. Israel | Law (1) | ‘From a broader perspective, it appears that the Genetic Model is based on the exaggerated importance that Western culture ascribes to biological origins and genetic identity. It invokes the myth of blood relation—’blood is thicker than water’—and considers relation by blood (that is, bio-genetic kinship) to be superior to any other.’ | Undefined | B5  A2ii  B1 |
| Zanghellini, A. (2009) [611] | 4. Australia | Law (1) | ‘Even leaving this aside, the argument is based merely on anecdotal evidence; it obscures how, regardless of biological connection, children’s identities are negotiated and solidified through meaningful interpersonal relationships in many adoptive, same-sex and non-white families; and it fails to interrogate the social and cultural practices responsible for mythologising biological connection, and of which the difficulties experienced by people who perceive a loss of genetic identity are probably an artefact.’ | Undefined | A1  B1 |
| Zeiler, K. (2007) [612] | 2. Sweden | Medical Humanities (3) | ‘Futhermore, genomic identity needs to be distinguished from genetic identity. In order for someone A to be genomically identical, the total genetic information in her or his cells needs to be the same at time t and time t’. The total genetic information in human cells comprises both the complex nuclear genome and the simple mitochondrial genome. If A is genetically identical, A is the same as regards a certain gene or certain genes (and not necessarily the whole genome) at time t and t’. | Defined | A2i  E2  E3  E6 |
| Zhao, J. (1996) [613] | 1. USA | Medicine (4) | ‘A hypothesis, called genomic individuality, is proposed, simply saying that every individual somatic genome, perhaps with rare exceptions, has its own unique or individual 'genetic identity' or 'fingerprint', which is characterized by its distinctive sequences or patterns of deoxyribonucleic acid molecules, or both.’  ‘On one hand, most, if not all, the somatic genomes of an organism are identical to each other in the great majority of the genomic characters that cast, as a whole, the genetic identity of an organism, but on the other hand, all of them are individually different from each another in some aspects that create a wide variety of genomic variability within that organism.’ | Undefined | A2ii  E6  E1 |
| Zhou, J. et al (2013) [614] | 5. China | Medicine (4) | ‘Cryptorchidism is a common congenital birth defect in human beings with the possible complication of infertility. (…) These cells could be differentiated into cells of all 3 germ layers in teratomas and in vitro, including into the VASA-positive germ cell lineage. Both parental urine cells and the reprogrammed cells possessed the normal karyotype and the same short tandem repeat loci, indicating that these 2 cell population share the same genetic identity.’ | Undefined | F1 |
| Zoloth, L. (2008) [615] | 1. USA | Medical Humanities (3) | ‘Jews confront a particular history in which Jewish genetics was the basis for the abuse of state authority. This link between inheritable genetic characteristics and social behavior begins in medieval ideology and blood libels and culminates in the Holocaust (…). The question of genetic identity was linked to the premise noted above, that Jews are both a religious grouping and an ethnic group with a shared genetic fate. Ashkenazi Jews, for example, have been noted as the critical population study group for an increasing number of disorders and diseases, including Tay Sachs, BRCA 1 and 2, Canavan, and depression. Such findings have potentiated a widespread popular anxiety about marking and discriminatory practices.’ | Undefined | A2ii  A3i  B1  B3  B2  D1 |
| Zoloth, L. et al. (2003) [616] | 1. USA | Medical Humanities (3) | ‘Further, traditional rules of conversion place obligations on both the community and the new member of the community, which transcend genetic ties and endorse the new, non-genetic identity as the actual one. What will this new determinant of Jewish identity mean for such a socio-legal system? The African Jewish stories raise fulcrum issues for the Jewish community in particular, and for the wider community as a whole, about narrative and description and obligation. The Lemba are only the beginning of this new way of knowing, for as our ability to make visible the invisible inner world and hence the ‘real self’ accelerates, it affects the way we see – and see morally – as well.’ | Undefined | B1  A3i |

References

1. Abbing HDCR. New Developments in International Health Law News and Views. Eur J Health L. 1998;5:155-70. PubMed PMID: rayyan-15370389.

2. Abrams K, Garrett BL. DNA and Distrust. Notre Dame L Rev. 2015;91(22):757-814. PubMed PMID: rayyan-15371881.

3. Abrams K, Piacenti RK. Immigration's Family Values. Va L Rev. 2014;100(4):629-710. PubMed PMID: rayyan-15371736.

4. Abuissa H, O'Keefe JH, Jr., Cordain L. Realigning our 21st century diet and lifestyle with our hunter-gatherer genetic identity. Directions in Psychiatry. 2005;25:SR1-SR10. PubMed PMID: 2006-02976-001.

5. Adams KE. Ethical Considerations of Applications of Preimplantation Genetic Diagnosis in the United States Genetics. Med & L. 2003;22:489-94. PubMed PMID: rayyan-15371498.

6. Aglaguel A, Abdelghaffar H, Ailal F, Habti N, Hesse S, Kohistani N, et al. Poikiloderma with Neutropenia in Morocco: a Report of Four Cases. J Clin Immunol. 2017;37(4):357-62. PubMed PMID: 28353165.

7. Ahmadi M, Ahmadi L. European Patent Law Framework regarding Nanotechnology Applications in Stem Cells International. Nanotech L & Bus. 2013;10:65-104. PubMed PMID: rayyan-15369940.

8. Ajunwa I. Genetic Testing Meets Big Data: Tort and Contract Law Issues Symposium: Torts and Civil Rights Law: Migration and Conflict. Ohio St LJ. 2014;75(6):1225-62. PubMed PMID: rayyan-15372159.

9. Alexander FS. Three Fallacies of Contemporary Jurisprudence. Loy L A L Rev. 1985;19:1-36. PubMed PMID: rayyan-15369495.

10. Alfano JA. Look What Katz Leaves Out: Why DNA Collection Challenges the Scope of the Fourth Amendment Notes. Hofstra L Rev. 2005;33:1017-48. PubMed PMID: rayyan-15372075.

11. Aloni E. Cloning and the LGBTI Family: Cautious Optimism Symposium: From Page to Practice: Broadening the Lens for Reproductive and Sexual Rights. NYU Rev L & Soc Change. 2011;35:1-80. PubMed PMID: rayyan-15369522.

12. Alpa G. The Protection of Privacy in Italian Law: Case Law in a Codified Legal System. Tul Eur & Civ LF. 1997;12:1-24. PubMed PMID: rayyan-15369486.

13. Alpert RT. What is a Jew?: The meaning of genetic disease for Jewish identity. The Reconstructionist. 2007;71(2):69-84. PubMed PMID: ATLA0001638364.

14. Alsgaard H. Decoupling Marriage & Procreation: A Feminist Argument for Same-Sex Marriage Recent Developments. Berkeley J Gender L & Just. 2012;27:307-38. PubMed PMID: rayyan-15370982.

15. Alvare HM. The Case for Regulating Collaborative Reproduction: A Children's Rights Perspective. Harv J on Legis. 2003;40:1-64. PubMed PMID: rayyan-15369513.

16. Amer MS. Breaking the Mold: Human Embryo Cloning and Its Implications for a Right to Individuality Comment. UCLA L Rev. 1995;43:1659-88. PubMed PMID: rayyan-15372261.

17. Anca MH, Gazit E, Loewenthal R, Ostrovsky O, Frydman M, Giladi N. Different phenotypic expression in monozygotic twins with Huntington disease. Am J Med Genet A. 2004;124(1):89-91. PubMed PMID: 14679593.

18. Anderlik MR, Rothstein MA. DNA-Based Identity Testing and the Future of the Family: A Research Agenda The Genetics Revolution: Conflicts, Challenges and Conundra. Am JL & Med. 2002;28:215-32. PubMed PMID: rayyan-15370641.

19. Anderson ML. Are You My Mommy - A Call for Regulation of Embryo Donation Comment. Cap U L Rev. 2006;35:589-626. PubMed PMID: rayyan-15371679.

20. Andrews LB. Is There a Right to Clone Constitutional Challenges to Bans on Human Cloning Symposium: Privacy, Property and Family in the Age of Genetic Testing. Harv J L & Tech. 1998;11:643-82. PubMed PMID: rayyan-15371754.

21. Andrieu J, Burke L, Chiang MHPD, Hong E. Defendant Dolly's Motion for Summary Judgment: Memorandum of Points and Authorities in Support Thereof Symposium: At the Crossroads of Law & Technology: Third Annual Conference. Loy L A L Rev. 2001;35:999-1030. PubMed PMID: rayyan-15372066.

22. Annas GJ. The Legacy of the Nuremberg Doctors' Trial to American Bioethics and Human Rights. Minn JL Sci & Tech. 2008;10(1):19-70. PubMed PMID: rayyan-15369673.

23. Archard D. Do Parents Own Their Children. Int'l J Child Rts. 1993;1:293-302. PubMed PMID: rayyan-15370937.

24. Armstrong D, Michie S, Marteau T. Revealed identity: a study of the process of genetic counselling. Soc Sci Med. 1998;47(11):1653-8. PubMed PMID: 9877335.

25. Attanasio JB. The Genetic Revolution: What Lawyers Don't Know Review Essay. NYU L Rev. 1988;63:662-716. PubMed PMID: rayyan-15371780.

26. Atwill N. Human Cloning: French Legislation and European Initiatives. Int'l J Legal Info. 2000;28:500-11. PubMed PMID: rayyan-15371515.

27. Atzmon G, Hao L, Pe'er I, Velez C, Pearlman A, Palamara PF, et al. Abraham's children in the genome era: major Jewish diaspora populations comprise distinct genetic clusters with shared Middle Eastern Ancestry. Am J Hum Genet. 2010;86(6):850-9. PubMed PMID: 20560205.

28. Bady P, Diserens AC, Castella V, Kalt S, Heinimann K, Hamou MF, et al. DNA fingerprinting of glioma cell lines and considerations on similarity measurements. Neuro Oncol. 2012;14(6):701-11. PubMed PMID: 22570425.

29. Bahri R, Esteban E, Halima AB, Moral P, Chaabani H. Distinctive genetic signatures of Alu/STR compound systems revealed by analyses of Mediterranean and Middle East populations. Anthropological science. 2014;122(2):81-8. PubMed PMID: 1586105964; 4603642.

30. Baker KK. The DNA Default and Its Discontents: Establishing Modern Parenthood. BU L Rev. 2016;96:2037-92. PubMed PMID: rayyan-15372305.

31. Baker TS. After Warnock. Law & Just - Christian L Rev. 1987;94:92-8. PubMed PMID: rayyan-15370076.

32. Bangert BC. Abortion: its social and ethical issues: an invitation to responsibility and moral discourse. Foundations. 1979;22(3):198-217. PubMed PMID: ATLA0001429588.

33. Barasoain M, Barrenetxea G, Ortiz-Lastra E, Gonzalez J, Huerta I, Telez M, et al. Single nucleotide polymorphism and FMR1 CGG repeat instability in two Basque valleys. Ann Hum Genet. 2011;76(2):110-20. PubMed PMID: 22211843.

34. Barnes EB. Gattaca and A.I.: artificial intelligence: views of salvation in an age of genetic engineering. Review & Expositor. 2002;99(1):59-70. PubMed PMID: ATLA0001401130.

35. Barnett DL. In Vitro Fertilization: Third Party Motherhood and the Changing Definition of Legal Parent Comments. Pac L J. 1985;17:231-60. PubMed PMID: rayyan-15370704.

36. Baron PD. In the Name of the Father: The Paternal Function, Sexuality, Law and Citizenship. Victoria U Wellington L Rev. 2006;37:307-34. PubMed PMID: rayyan-15370981.

37. Basset UC. What Is a Family - Exploring the Juridical Ground of Familism Today. Int'l J Jurisprudence Fam. 2012;3:301-28. PubMed PMID: rayyan-15370964.

38. Basu D, Salgado CM, Bauer BS, Johnson D, Rundell V, Nikiforova M, et al. Nevospheres from neurocutaneous melanocytosis cells show reduced viability when treated with specific inhibitors of NRAS signaling pathway. Neuro Oncol. 2016;18(4):528-37. PubMed PMID: 26354928.

39. Beadle L. Selling the Stem Cell Short - An Assessment of the Patentability of the Results of Human Stem Cell Research in New Zealand Canterbury Law Review Prize 2003. Canterbury L Rev. 2004;10:1-35. PubMed PMID: rayyan-15369494.

40. Becker SW. Erring on the Side of Justice: A Call for an End to Prosecutorial Arrogance in Opposing DNA Testing for Evidence Untested at Trial - Lessons of Innocence and Humility from the Case of Dean Cage Criminal Justice Series. DePaul J Soc Just. 2009;2:191-216. PubMed PMID: rayyan-15370536.

41. Beh HG, Diamond M. An Emerging Ethical and Medical Dilemna: Should Physicians Perform Sex Assignment Surgery on Infants with Ambiguous Genitalia. Mich J Gender & L. 2000;7:1-64. PubMed PMID: rayyan-15369514.

42. Bell D. Human Cloning and International Human Rights Law. Sydney L Rev. 1999;21:202-30. PubMed PMID: rayyan-15370578.

43. Bennett B. Genetics and the Transformation of the Personal. Monash U L Rev. 2009;35:296-314. PubMed PMID: rayyan-15370949.

44. Bently L, Sherman B. The Ehtics of Patenting: Towards a Transgenic Patent System. Med L Rev. 1995;3:275-91. PubMed PMID: rayyan-15370868.

45. Berg KD, Murphy KM. Floaters in surgical pathology tissues genetic identity testing potential and pitfalls. Pathology Case Reviews. 2003;8(3):103-10. PubMed PMID: rayyan-15370132.

46. Bergeron JHL, JuliaInsani, RobertNikolopoulou, Charikleia. European Law Foreign Law Year in Review: 2001: Introduction. Int'l L. 2002;36:855-78. PubMed PMID: rayyan-15371968.

47. Black J. Regulation as Facilitation: Negotiating the Genetic Revolution Human Genetics and the Law: Regulating a Revolution. Mod L Rev. 1998;61:621-60. PubMed PMID: rayyan-15371724.

48. Blake VK. Ovaries, Testicles, and Uteruses, Oh My - Regulating Reproductive Tissue Transplants. Wm & Mary J Women & L. 2013;19:353-94. PubMed PMID: rayyan-15371142.

49. Blyth E. The United Kingdom's Human Fertillisation and Embryology Act 1990 and the Welfare of the Child: A Critique. Int'l J Child Rts. 1995;3:417-38. PubMed PMID: rayyan-15371332.

50. Blyth E. Parental orders and identity registration: One country three systems. Journal of Social Welfare and Family Law. 2010;32(4):345-52. PubMed PMID: rayyan-15371117.

51. Blyth E, Farrand A. Anonymity in Donor-Assisted Conception and the UN Convention on the Rights of the Child. Int'l J Child Rts. 2004;12:89-104. PubMed PMID: rayyan-15370061.

52. Blyth E, Frith L. The UK's Gamete Donor Crisis - A Critical Analysis. Critical Soc Pol'y. 2008;28:74-95. PubMed PMID: rayyan-15369985.

53. Bordet S, Feldman S, Knoppers BM. Legal Aspects of Animal-Human Combinations in Canada. McGill Health L Publ'n. 2007;1:83-100. PubMed PMID: rayyan-15370030.

54. Borecki IB, Blangero J, Rice T, Perusse L, Bouchard C, Rao DC. Evidence for at least two major loci influencing human fatness. Am J Hum Genet. 1998;63(3):831-8. PubMed PMID: 9718336.

55. Borowski CM. Human Cloning Research in Japan: A Study in Science, Culture, Morality, and Patent Law Comment. Ind Int'l & Comp L Rev. 1999;9:505-36. PubMed PMID: rayyan-15371529.

56. Bozzato G. The early human embryo is scarred by terms of ambiguous meaning. Linacre Quarterly. 2010;77(4):445-67. PubMed PMID: rayyan-15371401.

57. Bradley GV. A Case for Proposition 209 Symposium on Race and the Law. Notre Dame JL Ethics & Pub Pol'y. 1997;11:97-120. PubMed PMID: rayyan-15370106.

58. Brannigan MC. Surrogate Motherhood: The Ethics of Using Human Beings. Cross Currents. 1989;39(1):121. PubMed PMID: ATLA0000581541.

59. Brodeala E. The Legal Status of Assisted Human Reproduction in Romania. A Brief Discussion on Surrogacy Section I: Articles. Rom J Comp L. 2016;7:56-74. PubMed PMID: rayyan-15369887.

60. Brodie DW. The New Biology and the Prenatal Child. J Fam L. 1970;9:391-407. PubMed PMID: rayyan-15371262.

61. Brown ME, Rondon E, Rajesh D, Mack A, Lewis R, Feng XZ, et al. Derivation of Induced Pluripotent Stem Cells from Human Peripheral Blood T Lymphocytes. Plos One. 2010;5(6):9. PubMed PMID: WOS:000279369900021.

62. Brownsword R, Somsen H. Law, Innovation and Technology: Before We Fast Forward - A Forum for Debate. Law Innovation & Tech. 2009;1:1-74. PubMed PMID: rayyan-15369521.

63. Brumnik R, Podbregar I. Biometric Technology and Human Rights Monographic Study. US-China Law Review. 2010;7:1-11. PubMed PMID: rayyan-15369460.

64. Brunk CG. [Reply to A Voth, 'Christian principles in medical/ethical dilemmas', 6:29-44 Wint 1988]. The Conrad Grebel Review. 1988;6(2):167-73. PubMed PMID: ATLA0000806540.

65. Buckle S, Dawson K, Singer P. Syngamy Debate: When Precisely Does a Human Life Begin, The International Review Essay. L Med & Health Care. 1989;17:174-81. PubMed PMID: rayyan-15370471.

66. Bugert P, Rink G, Kemp K, Kluter H. Blood Group ABO Genotyping in Paternity Testing. Transfus Med Hemother. 2012;39(3):182-6. PubMed PMID: 22851933.

67. Cahill LS. No Human Cloning: A Social Ethics Perspective Symposium on Human Cloning: Legal, Social, and Moral Perspectives for the Twenty-First Century. Hofstra L Rev. 1999;27:487-502. PubMed PMID: rayyan-15371492.

68. Cahn N. The New Kinship. Geo LJ. 2012;100:367-430. PubMed PMID: rayyan-15371189.

69. Cahn N, Singer J. Adoption, Identity, and the Constitution: The Case for Opening Closed Records Symposium: Existing and Emerging Constitutional Rights of Children: Commentary. U Pa J Const L. 1999;2:150-94. PubMed PMID: rayyan-15370358.

70. Callahan SC. New Genetic Choices. The Living Pulpit. 1995;4(3):20-1. PubMed PMID: ATLA0000919724.

71. Callus T. Tempered Hope - A Qualified Right to Know One's Genetic Origin: Odievre v France Cases. Mod L Rev. 2004;67:658-69. PubMed PMID: rayyan-15371774.

72. Campbell RB. On the robustness of regular systems of inbreeding. Math Biosci. 1991;104(1):1-19. PubMed PMID: 1804450.

73. Campbell RB. The effect of inbreeding constraints and offspring distribution on time to the most recent common ancestor. Journal of Theoretical Biology. 2015;382:74-80. PubMed PMID: rayyan-15369983.

74. Campiglio C. Human Genetics, Reproductive Technology and Fundamental Rights. Italian YB Int'l L. 2004;14:83-120. PubMed PMID: rayyan-15370031.

75. Canellopoulou-Bottis M. The Implementation of the European Directive 95/46/EC in Greece and Medical/Genetic Data The Protection of Individuals with Regard to the Processing of Personal Data with Special Regard to Medical Data. Eur J Health L. 2002;9:207-18. PubMed PMID: rayyan-15370601.

76. Caplan AL. What If Anything Is Wrong with Cloning a Human Being International Arbitrage of Controversial Medical Technologies. Case W Res J Int'l L. 2003;35:369-84. PubMed PMID: rayyan-15371193.

77. Capps WH. Keeping pace with the code of codes. The Christian Century. 1993;110(11):373-5. PubMed PMID: ATLA0000862111.

78. Carbone J, Gottheim P. Markets, Subsidies, Regulation, and Trust: Building Ethical Understandings into the Market for Fertility Services Symposium: Creating Life - Examining the Legal, Ethical, and Medical Issues of Assisted Reproductive Technologies. J Gender Race & Just. 2005;9:509-48. PubMed PMID: rayyan-15371538.

79. Carmel JB, Willis DE. Neural circuits catch fire. Neurotherapeutics. 2016;13(2):261-3. PubMed PMID: 2016-17591-001.

80. Carpenter BC. Sex Post Facto: Advising Clients regarding Posthumous Conception. ACTEC LJ. 2012;38:187-228. PubMed PMID: rayyan-15370522.

81. Casey RP. A new American compact: caring about women, caring for the unborn. First Things. 1992;27:43-6. PubMed PMID: ATLA0000856917.

82. Caterina JR. Glorious Bastards: The Legal and Civil Birthright of Adoptees to Access Their Medical Records in Search of Genetic Identity Note. Syracuse L Rev. 2010;61:145-72. PubMed PMID: rayyan-15370330.

83. Chatzinikolaou NSM. From ethics of dilemmas to theology of transcendence. St Vladimir's Theological Quarterly. 2010;54(2):165-88. PubMed PMID: ATLA0001799529.

84. Chen IC, H, ez C, Xu X, Cooney A, Wang Y, et al. Dynamic Variations in Genetic Integrity Accompany Changes in Cell Fate. Stem Cells Dev. 2016;25(22):1698-708. PubMed PMID: 27627671.

85. Cho YK. Genetically encoded tools: bridging the gap between neuronal identity and function. ACS Chem Neurosci. 2015;6(1):14-5. PubMed PMID: 25574970.

86. Choi J. California and the Future of Partial Match DNA Investigations Note. Hastings Const LQ. 2011;39:713-38. PubMed PMID: rayyan-15371842.

87. Chorpening J. Genetic Disability: A Modest Proposal to Modify the ADA to Protect against Some Forms of Genetic Discrimination Comment. NC L Rev. 2004;82:1441-81. PubMed PMID: rayyan-15372221.

88. Christgen M, Bruchhardt H, Hadamitzky C, Rudolph C, Steinemann D, Gadzicki D, et al. Comprehensive genetic and functional characterization of IPH-926: a novel CDH1-null tumour cell line from human lobular breast cancer. J Pathol. 2008;217(5):620-32. PubMed PMID: 19191266.

89. Chua H. Designer Babies and the Law: A Legal Analysis of Human Germline Editing in Light of the UK's Human Rights Obligations. King's Student L Rev. 2017;8:68-87. PubMed PMID: rayyan-15369955.

90. Chumakov PM. Versatile functions of p53 protein in multicellular organisms. Biochemistry (Mosc). 2007;72(13):1399-421. PubMed PMID: 18282133.

91. Cicero C. The Italian Reform of the Law on Filiation and Constitutional Legality Essays. Italian LJ. 2016;2:237-52. PubMed PMID: rayyan-15370723.

92. Ciciarello M, Mangiacasale R, Lavia P. Spatial control of mitosis by the GTPase Ran. Cell Mol Life Sci. 2007;64(15):1891-914. PubMed PMID: 17483873.

93. Clark B. A Balancing Act: The Rights of Donor-Conceived Children to Know Their Biological Origins. Ga J Int'l & Comp L. 2011;40:619-62. PubMed PMID: rayyan-15371719.

94. Clayton EW. Ten Fingers, Ten Toes: Newborn Screening for Untreatable Disorders Proceedings: Newborn Screening for Nontreatable Disorders. Health Matrix. 2009;19:199-204. PubMed PMID: rayyan-15370566.

95. Cleal B, Gamble N. Mitochondrial Donation in the United Kingdom. SciTech Law. 2015;12:18-21. PubMed PMID: rayyan-15369665.

96. Cobbe N. Cross-species chimeras: exploring a possible Christian perspective. Zygon. 2007;42(3):599-628. PubMed PMID: ATLA0001602149.

97. Cockfield A. Surveillance as Law Special Issue: The Laws of Technology and the Technology of Law. Griffith L Rev. 2011;20:795-816. PubMed PMID: rayyan-15371914.

98. Cockfield AJ. The State of Privacy Laws and Privacy-Encroaching Technologies after September 11: A Two-Year Report Card on the Canadian Government. U Ottawa L & Tech J. 2004;1:325-44. PubMed PMID: rayyan-15371057.

99. Cockfield AJ. Who Watches the Watchers - A Law and Technology Perspective on Government and Private Sector Surveillance. Queen's LJ. 2003;29:364-407. PubMed PMID: rayyan-15371175.

100. Cohen DM. Cloning and the Constitution, Cloning and the Constitution, Cloning and the Constitution, Cloning and. Nova L Rev. 2001;26:511-44. PubMed PMID: rayyan-15371546.

101. Cohen IG. Regulating Reproduction: The Problem with Best Interests. Minn L Rev. 2011;96:423-519. PubMed PMID: rayyan-15371346.

102. Cohen IG. Beyond Best Interests. Minn L Rev. 2012;96:1187-274. PubMed PMID: rayyan-15372143.

103. Cohen IG, Coan TG. Can You Buy Sperm Donor Identification; An Experiment. J Empirical Legal Stud. 2013;10:715-40. PubMed PMID: rayyan-15371845.

104. Cohen J. Federal Issues in Trade Secret Law. J High Tech L. 2003;2:1-44. PubMed PMID: rayyan-15369503.

105. Collins E. Do You Know Where Your DNA Is - The Need for DNA Legislation in Ohio Note. JL & Health. 2013;26:348-74. PubMed PMID: rayyan-15371128.

106. Conley JM, Makowski R. Back to the Future: Rethinking the Product of Nature Doctrine as a Barrier to Biotechnology Patents (Part I). J Pat & Trademark Off Soc'y. 2003;85:301-34. PubMed PMID: rayyan-15370965.

107. Conley JM, Makowski R. Back to the Future: Rethinking the Product of Nature Doctrine as a Barrier to Biotechnology Patents (Part II). J Pat & Trademark Off Soc'y. 2003;85:371-98. PubMed PMID: rayyan-15371203.

108. Conley JM, Makowski R. Rethinking the Product of Nature Doctrine as a Barrier to Biotechnology Patents in the United States - and Perhaps Europe as Well. Info & Comm Tech L. 2004;13:3-40. PubMed PMID: rayyan-15369542.

109. Connolly U. Paternity Fraud and the Tort of Deceit. Q Rev Tort L. 2008;3:24-30. PubMed PMID: rayyan-15369706.

110. Constand S. Patently a Problem - Recent Developments in Human Gene Patenting and Their Wider Ethical and Practical Implications. QUT L Rev. 2013;13:100-25. PubMed PMID: rayyan-15370116.

111. Crespi GS. Would It Be Unethical to Dump Radioactive Wastes in the Ocean - The Surprising Implications of the Person-Altering Consequences of Policies. Ecology L Currents. 2008;35:43-52. PubMed PMID: rayyan-15369822.

112. Crespi GS. Incorporating Endogenous Preferences in Cost-Benefit Analysis. Penn St Envtl L Rev. 2009;17:157-90. PubMed PMID: rayyan-15370398.

113. Crespi GS. How Recognizing the Endogeneity of Identity Renders the Discounting Debate Largely Irrelevant. J Land Resources & Envtl L. 2010;30:75-94. PubMed PMID: rayyan-15369995.

114. Crespi GS. The Endogeneity Problem in Cost-Benefit Analysis. Geo JL & Pub Pol'y. 2010;8:91-146. PubMed PMID: rayyan-15370074.

115. Cronin AJ, Douglas JF. Non-Standard Kidneys for Transplants: Clinical Margins, Medical Morality, and the Law. Med L Rev. 2013;21:448-73. PubMed PMID: rayyan-15371407.

116. Culty M. Gonocytes, the forgotten cells of the germ cell lineage. Birth Defects Res C Embryo Today. 2009;87(1):1-26. PubMed PMID: 19306346.

117. Da Costa Francez PA, Rodrigues EM, de Velasco AM, dos Santos SE. Insertion-deletion polymorphisms--utilization on forensic analysis. Int J Legal Med. 2012;126(4):491-6. PubMed PMID: 21647760.

118. Daar JF. The Future of Human Cloning: Prescient Lessons from Medical Ethics Past Symposium on Cloning. S Cal Interdisc L J. 1998;8:167-84. PubMed PMID: rayyan-15370443.

119. Dal Cin P, Van den Berghe H. Ten years of the cytogenetics of soft tissue tumors. Cancer Genet Cytogenet. 1997;95(1):59-66. PubMed PMID: 9140454.

120. Das B, Tao SZ, Mushnitsky R, Norin AJ. Genetic identity and differential expression of p38.5 (Haymaker) in human malignant and nonmalignant cells. Int J Cancer. 2001;94(6):800-6. PubMed PMID: 11745481.

121. Davies JR, Svitacheva N, Lannefors L, Kornfalt R, Carlstedt I. Identification of MUC5B, MUC5AC and small amounts of MUC2 mucins in cystic fibrosis airway secretions. Biochem J. 1999;344:321-30. PubMed PMID: 10567212.

122. de Andrade NN. Human Genetic Manipulation and the Right to Identity: The Contradictions of Human Rights Law in Regulating the Human Genome. SCRIPTed. 2010;7:429-52. PubMed PMID: rayyan-15371362.

123. De Meeus T. Revisiting FIS, FST, Wahlund effects and null alleles. J Hered. 2018. PubMed PMID: 29165594.

124. DeBre KD. Patents on People and the U.S. Constitution: Creating Slaves or Enslaving Science Note. Hastings Const LQ. 1989;16:221-60. PubMed PMID: rayyan-15370664.

125. Deckers J. Why Eberl is wrong. Reflections on the beginning of personhood. Bioethics. 2007;21(5):270-82. PubMed PMID: 2007-06810-004.

126. DeCoursey TE, Morgan D, Cherny VV. The gp91phox component of NADPH oxidase is not a voltage-gated proton channel. Journal of General Physiology. 2002;120(6):773-9. PubMed PMID: rayyan-15371894.

127. Deech R. Family Law and Genetics Human Genetics and the Law: Regulating a Revolution. Mod L Rev. 1998;61:697-715. PubMed PMID: rayyan-15371818.

128. Deech RL. Clones, Ethics and Infertility or Sex, Sheep and Statutes Symposium: Privacy and Autonomy in Health Care. Quinnipiac Health L J. 1999;2:117-34. PubMed PMID: rayyan-15370206.

129. den Dunnen JT. The DNA Bank: High-Security Bank Accounts to Protect and Share Your Genetic Identity. Hum Mutat. 2015;36(7):657-9. PubMed PMID: 25952467.

130. Deng L, Hoh BP, Lu D, Saw WY, Twee-Hee Ong R, Kasturiratne A, et al. Dissecting the genetic structure and admixture of four geographical Malay populations. Sci Rep. 2015;5:14375. PubMed PMID: 26395220.

131. Dennison M. Revealing Your Sources: The Case for Non-Anonymous Gamete Donation. JL & Health. 2008;21:1-28. PubMed PMID: rayyan-15369488.

132. Destro RA. Quality-of-Life Ethics and Constitutional Jurisprudence: The Demise of Natural Protection for the Disabled and Incompetent. J Contemp Health L & Pol'y. 1986;2:71-130. PubMed PMID: rayyan-15369973.

133. Deutsch J. Finders-Keepers: A Bright-Line Rule Awarding Custody to Gestational Mothers in Cases of Fertility Clinic Error Notes. Cardozo JL & Gender. 2005;12:367-90. PubMed PMID: rayyan-15371187.

134. Dickens BM. The Ectogenetic Human Being:A Problem Child of Our Time. U W Ontario L Rev. 1979;18:241-68. PubMed PMID: rayyan-15370743.

135. Dickman DG. Social Values in a Brave New World: Toward a Public Policy Regarding Embryo Status and in Vitro Fertilization Comment. St Louis U LJ. 1985;29:817-52. PubMed PMID: rayyan-15371935.

136. Dieterlen F, Lucotte G. Haplotype XV of the Y-chromosome is the main haplotype in West-Europe. Biomed Pharmacother. 2005;59(5):269-72. PubMed PMID: 15890489.

137. DiFonzo JH. The Crimes of Crime Labs Ideas & Essays. Hofstra L Rev. 2005;34:1-12. PubMed PMID: rayyan-15369465.

138. DiFonzo JH, Stern RC. Devil in a White Coat: The Temptation of Forensic Evidence in the Age of CSI Symposium: The CSI Effect: The True Effect of Crime Scene Television on the Justice System. New Eng L Rev. 2007;41:503-32. PubMed PMID: rayyan-15371521.

139. Dimond R, Bartlett A, Lewis J. What binds biosociality? The collective effervescence of the parent-led conference. Soc Sci Med. 2014;126:1-8. PubMed PMID: 25497725.

140. Dolgin JL. Biological Evaluations: Blood, Genes, and Family. Akron L Rev. 2008;41:347-98. PubMed PMID: rayyan-15371125.

141. Dove ES. Back to Blood: The Sociopolitics and Law of Compulsory DNa Testing of Refugees Trends and Issues in Immigration and the Law. U Mass L Rev. 2013;8:466-531. PubMed PMID: rayyan-15371442.

142. Dowd NE. From Genes, Marriage and Money to Nurture: Redefining Fatherhood Essay. Cardozo Women's LJ. 2003;10:132-45. PubMed PMID: rayyan-15370277.

143. Dowd NE. Fathers and the Supreme Court: Founding Fathers and Nuturing Fathers. Emory LJ. 2005;54:1271-334. PubMed PMID: rayyan-15372176.

144. Dowd NE. Parentage at Birth: Birthfathers and Social Fatherhood Symposium: Reforming Parentage Laws. Wm & Mary Bill Rts J. 2006;14:909-42. PubMed PMID: rayyan-15372009.

145. Dreyfuss RC, Dorothy. Jurisprudence of Genetics. Vand L Rev. 1992;45:313-48. PubMed PMID: rayyan-15371009.

146. Du Toit CW. The place of values in the science-religion dialogue: biology, human nature and the cultural environment. Journal of Theology for Southern Africa. 2002;113:75-95. PubMed PMID: ATLA0001322703.

147. Ducharme HM. Section 12: Philosophical and Conceptual Issues Conference Abstracts. JL Med & Ethics. 2001;29:34-7. PubMed PMID: rayyan-15369771.

148. Duddington J. The Legal and Ethical Aspects of Human Cloning - Part One Legal Aspects. Law & Just - Christian L Rev. 1999;140:26-42. PubMed PMID: rayyan-15369724.

149. Dunne LM. Come, Let us Return to Reason: Association of Molecular Pathology v. USPTO Case Note and Comment. DePaul J Art Tech & Intell Prop L. 2009;20:473-512. PubMed PMID: rayyan-15371466.

150. Dunstan GR. Catholics and the Warnock Enquiry. Law & Just - Christian L Rev. 1983;78:71-7. PubMed PMID: rayyan-15369967.

151. Dunstan GR. Screening for fetal and genetic abnormality: social and ethical issues. J Med Genet. 1988;25(5):290-3. PubMed PMID: 3385738.

152. Earl J, Rico D, Carrillo-de-Santa-Pau E, Rodríguez-Santiago B, Méndez-Pertuz M, Auer H, et al. Erratum: The UBC-40 Urothelial Bladder Cancer cell line index: A genomic resource for functional studies [BMC Genomics, 16 (2015), (1019)]. BMC Genomics. 2016;17(1). PubMed PMID: rayyan-15369395.

153. Ehlers VJ. The Case against Human Cloning Symposium on Human Cloning: Legal, Social, and Moral Perspectives for the Twenty-First Century. Hofstra L Rev. 1998;27:523-32. PubMed PMID: rayyan-15371563.

154. Elhauge E. I'm Not Quite Dead Yet - And Other Health Care Observations Symposium: Health Law Policy: Legal Issues in the Evolving Healthcare Market: Honoring the Work of Einer Elhauge. Tulsa L Rev. 2013;49:607-26. PubMed PMID: rayyan-15371705.

155. Elliott C, Brodwin P. Identity and genetic ancestry tracing. British Medical Journal. 2002;325(7378):1469-71. PubMed PMID: rayyan-15372224.

156. Ephross JN. In Vetro Fertilization: Perspectives on Current Issues Technote. Jurimetrics J. 1992;32:447-72. PubMed PMID: rayyan-15371406.

157. Esmaili S. Searching for a Needle in a Haystack: The Constitutionality of Police DNA Dragnets Symposium: 150th Anniversary of the Dred Scott Decisions: Student Note. Chi-Kent L Rev. 2007;82:495-524. PubMed PMID: rayyan-15371508.

158. Falliers CJ, de Cardoso RR, Bane HN, Coffey R, Middleton E, Jr. Discordant allergic manifestations in monozygotic twins: genetic identity versus clinical, physiologic, and biochemical differences. J Allergy. 1971;47(4):207-19. PubMed PMID: 4252251.

159. Fallone EA. Funding Stem Cell Research: The Convergence of Science, Religion & Politics in the Formation of Public Health Policy. Marq Elder's Adviser. 2010;12:247-94. PubMed PMID: rayyan-15370764.

160. Fergus VL. An Interpretation of Ohio Law on Maternal Status in Gestational Surrogacy Disputes: Belsito v. Clark, 644 N.E.2d 760 (Ohio C.P. Summit County 1994) Note. U Dayton L Rev. 1995;21:229-48. PubMed PMID: rayyan-15370694.

161. Ferragut JF, Marques SL, Ramon C, Castro JA, Amorim A, Alvarez L, et al. Founding mothers of Chueta population. Forensic Science International: Genetics Supplement Series. 2015;5:e492-e4. PubMed PMID: rayyan-15372477.

162. Ferre F. Holmes Rolston III, Genes, Genesis and God: Values and Their Origins in Natural and Human History. International Journal for Philosophy of Religion. 2000;47(3):179-82. PubMed PMID: rayyan-15370485.

163. Ferreira N. A Theory of Discrimination Law Book Review. Int'l J Discrimination & L. 2016;16:247-50. PubMed PMID: rayyan-15370760.

164. Feuer J. Relatively Speaking: Halachic and Legal Issues of Gamete Donation Assisted Reproduction. Med & L. 2011;30:239-66. PubMed PMID: rayyan-15370733.

165. Forman DL. Unwed Fathers and Adoption: A Theoretical Analysis in Context. Tex L Rev. 1994;72:967-1046. PubMed PMID: rayyan-15372047.

166. Fraga MG, Gagliardi FL, Szöcs AH, Alcázar DH, Solimine JH, Echenique CG, et al. Mitochondrial DNA control region database in Banco Nacional de Datos Genéticos, Argentina. Forensic Science International: Genetics Supplement Series. 2009;2(1):324-6. PubMed PMID: rayyan-15371052.

167. Franciosi LM, Attilio. The Protection of Genetic Identity Civil Law Workshop - Robert A. Pascal Series - Revisiting the Distinction between Persons and Things. J Civ L Stud. 2008;1:139-88. PubMed PMID: rayyan-15370308.

168. Frankel A. Cloning Human Beings and the Consumer of the Future: A Worthwhile Endeavor or a Nightmare come True. Loy Consumer L Rev. 2000;13:149-87. PubMed PMID: rayyan-15370354.

169. Franklin S. Re-thinking nature-culture: Anthropology and the new genetics. Anthropological Theory. 2003;3(1):65-85. PubMed PMID: rayyan-15369938.

170. Frazer KA, Ballinger DG, Cox DR, Hinds DA, Stuve LL, Gibbs RA, et al. A second generation human haplotype map of over 3.1 million SNPs. Nature. 2007;449(7164):851-61. PubMed PMID: 17943122.

171. Fredlake CP, Hert DG, Mardis ER, Barron AE. What is the future of electrophoresis in large-scale genomics sequencing? Electrophoresis. 2006;27(19):3689-702. PubMed PMID: rayyan-15372377.

172. Frith L. Beneath the rhetoric: the role of rights in the practice of non-anonymous gamete donation. Bioethics. 2001;15(5):473-84. PubMed PMID: 12058771.

173. Gabel JD. Probable Cause from Probable Bonds: A Genetic Tattle Tale Based on Familial DNA. Hastings Women's LJ. 2010;21:3-58. PubMed PMID: rayyan-15369543.

174. Gagnon A, Beise J, Vaupel JW. Genome-wide identity-by-descent sharing among CEPH siblings. Genet Epidemiol. 2005;29(3):215-24. PubMed PMID: 16121355.

175. Gamble J. Developing an in vitro assay for detection and characterization of functional connectivity within transplantation candidate embryonic stem cell-derived V2a interneuron networks. US DB - psyh DP - EBSCOhost: ProQuest Information & Learning; 2017.

176. Gandi H. Why No Backlash: Advances in Forensic Technology and the Criminalized Fourth Amendment Notes and Comments. Whittier L Rev. 2014;36:533-68. PubMed PMID: rayyan-15371586.

177. Gardner-Hopkins JDK. Unemployable Genes: Genetic Discrimination in the Workplace. Auckland U L Rev. 2000;9:435-69. PubMed PMID: rayyan-15371380.

178. Gargiulo PN, Giuseppe Diplomatic and Parliamentary Practice Italian YB Int'l L. 2000;10:317-50. PubMed PMID: rayyan-15371026.

179. Garstka K. From Cyberpunk to Regulation. J Intell Prop Info Tech & Elec Com L. 2017;8:293-303. PubMed PMID: rayyan-15370938.

180. Geisler NL. [When did I begin? by N Ford, 1988, review article]. Journal of the Evangelical Theological Society. 1990;33(4):509-12. PubMed PMID: ATLA0000835062.

181. Gerards JHJ, Heleen L. Regulation of Genetic and Other Health Information in a Comparative Perspective. Eur J Health L. 2006;13:339-98. PubMed PMID: rayyan-15371105.

182. Ghiggeri GM, Dagnino M, Parodi S, Zennaro C, Amoroso A, Pugliese F, et al. Discordant evolution of nephrotic syndrome in mono- and dizygotic twins. Pediatr Nephrol. 2006;21(3):419-22. PubMed PMID: 16382323.

183. Gianoli E, Valladares F. Studying phenotypic plasticity: The advantages of a broad approach. Biological Journal of the Linnean Society. 2012;105(1):1-7. PubMed PMID: rayyan-15369438.

184. Giddings KJ. The Current Status of the Right of Adult Adoptees to Know the Identity of Their Natural Parents Note. Wash U L Q. 1980;58:677-704. PubMed PMID: rayyan-15371796.

185. Godfrey JP. The Pope and the ontogeny of persons. Nature. 1995;373(6510):100. PubMed PMID: rayyan-15370115.

186. Goodwin JAM-VDW, Lirieka. The Use of DNA Evidence in South Africa: Powerful Tool or Prone to Pitfalls. S African LJ. 1997;114:151-73. PubMed PMID: rayyan-15370367.

187. Gorney R. The New Biology and the Future of Man. UCLA L Rev. 1967;15:273-356. PubMed PMID: rayyan-15370864.

188. Goswami S. Caught in the Middle: Reducing the Uncertainty Created by the FDA and the Patent System for Genetic Diagnostic Test Makers. NYU J Intell Prop & Ent L. 2012;1:33-78. PubMed PMID: rayyan-15369769.

189. Graham KA, Trent JM, Osborne CK, McGrath CM, Minden MD, Buick RN. The use of restriction fragment polymorphisms to identify the cell line MCF-7. Breast Cancer Res Treat. 1986;8(1):29-34. PubMed PMID: 3790748.

190. Graham KT. Same-Sex Couples: Their Rights as Parents, and Their Children's Rights as Children Symposium Article. Santa Clara L Rev. 2008;48:999-1038. PubMed PMID: rayyan-15372067.

191. Greely HT. Neuroethics and ELSI: Similarities and Differences Essay. Minn JL Sci & Tech. 2006;7:599-638. PubMed PMID: rayyan-15371691.
[truncated: 76,728 more chars]
